# Microbial Sharing Between Siblings Supports Metabolic Functions Protective Against Allergy

**DOI:** 10.1111/all.70033

**Published:** 2025-08-29

**Authors:** Hannah Devotta, Aonghus Lavelle, Katri Korpela, Sadhbh Hurley, Emer Shannon, Nonhlanhla Lunjani, Anoop Ambikan, Ujjwal Neogi, Carina Venter, Jens Walter, Jonathan Hourihane, Liam O'Mahony

**Affiliations:** ^1^ APC Microbiome Ireland, University College Cork Cork Ireland; ^2^ School of Microbiology University College Cork Cork Ireland; ^3^ Department of Anatomy and Neuroscience University College Cork Cork Ireland; ^4^ Human Microbiome Research Program, Faculty of Medicine University of Helsinki Helsinki Finland; ^5^ Department of Paediatrics, Cork University Hospital University College Cork Cork Ireland; ^6^ University Hospital Limerick Limerick Ireland; ^7^ The Systems Virology Lab, Division of Clinical Microbiology, Department of Laboratory Medicine Karolinska Institute, ANA Futura, Campus Flemingsberg Stockholm Sweden; ^8^ Section of Allergy & Immunology, Department of Pediatrics, Children's Hospital Colorado, University of Colorado School of Medicine Aurora Colorado USA; ^9^ Department of Medicine University College Cork Cork Ireland; ^10^ Paediatrics and Child Health Royal College of Surgeons in Ireland Dublin Ireland; ^11^ Children's Health Ireland Dublin Ireland

**Keywords:** environment, food allergy, hygiene hypothesis, microbiome

To the Editor,

1

The association between siblings and protection from atopic disorders was first described by Strachan in 1989 [1], a finding that formed the basis of the “hygiene hypothesis”. Multiple studies have since supported the association between birth order and allergic sensitization, potentially mediated via microbial exposures [2]. Microbe‐host interactions during early life help establish long‐term patterns of immune reactivity that influence the risk of immune‐mediated diseases such as allergies. We hypothesized that sibling‐associated changes in infant microbiome composition and functional potential may enhance immune regulatory programs that protect against allergies. To test this hypothesis, we compared metagenomic sequencing data from infants with or without siblings born during strict pandemic‐enforced social distancing measures (CORAL study) [3] and identified the sibling‐associated microbes and gene families that correlated with protection from food allergen sensitization.

Birth mode, type of feeding, home location or type of dwelling, pet ownership, childcare arrangements, smoking in the home or use of antibiotics for infants with (*n* = 187) or without (*n* = 164) siblings were similar for both groups (Table S1). Of the infants with older siblings, 130 had one sibling, 41 had two siblings, and 16 had three siblings. Alpha diversity was not significantly different between infants with or without siblings (Figure S1), but beta diversity was significantly different at both 6 (*p* = 0.002) and 12 (*p* = 0.001) months of age (Figure 1a,b), associated with significant changes in relative abundance of specific taxa that remained significant following adjustments for breastfeeding, birth mode, and external environmental factors (Figure 1c,d and Table S2). The number of siblings did not significantly affect these associations. Non‐spore‐forming taxa such as *Bifidobacterium* species were enriched in infants with siblings, suggesting that living with older siblings overcomes the spatial and temporal barriers usually associated with the transfer of non‐spore‐forming microbes.

**FIGURE 1:**
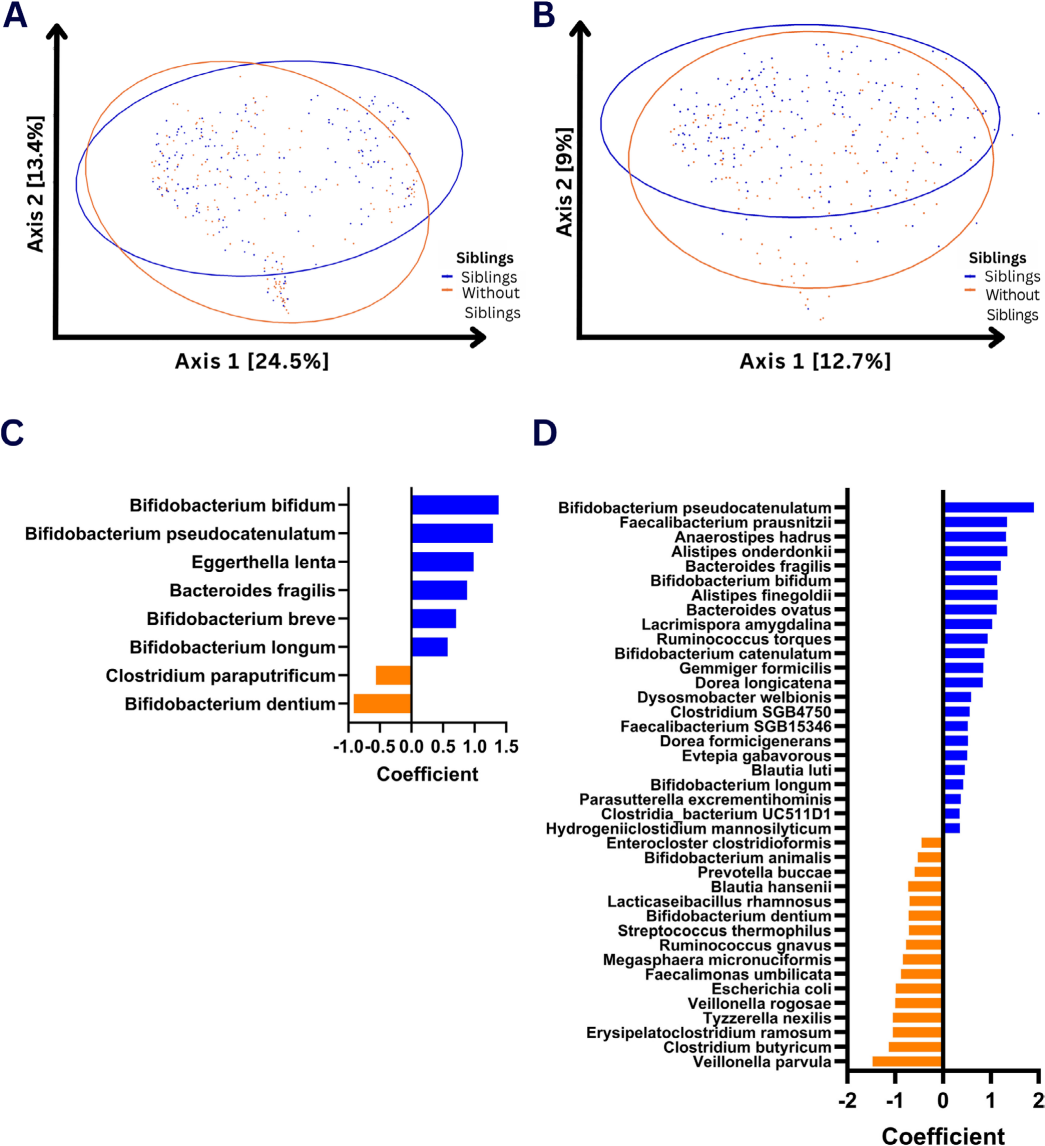
Changes in microbial taxa associated with siblings. Principal component plots for sibling effects on microbial beta diversity at 6 (A) and 12 (B) months of age. Individual FDR adjusted significant taxa differences for 6 (C) and 12 (D) months of age.

The overall relative abundance of gene families was not significantly different at 6 months of age (*p* = 0.108) but was significantly different at 12 months of age (*p* = 0.006) for infants with siblings that remained significant (*p* = 0.007) following adjustments for breastfeeding, birth mode, and external environmental factors (Figure S2). Of the 4018 gene families identified in more than 20% of 6‐month‐old infants, 534 were significantly associated with having siblings, and 5 gene families remained significant following adjustment (Table S3). Gene Set Enrichment Analysis (GSEA) identified one pathway that was significantly different between infants with or without siblings at 6 months (Figure S3). At 12 months of age, 1237 gene families were significantly associated with siblings following adjustment (Table S4), with highly significant GSEA enrichments observed (Figure 2a,b). Significant enrichments were also observed in the previously described gut‐brain modules (GBMs) [4]. At 6 months of age, infants with siblings displayed enrichment in 4 modules, but these did not remain statistically significant following FDR adjustment (Figure 2c and Table S5). At 12 months of age, 16 modules remained significantly different following FDR adjustment (Figure 2d and Table S6). Modules relating to short‐chain fatty acid (SCFA) metabolism and tryptophan metabolism were influenced by the presence of siblings, with an overall predicted increase in potential microbial metabolic output. Both SCFAs and tryptophan‐derived indoles are well described as immunoregulatory metabolites [5, 6].

**FIGURE 2:**
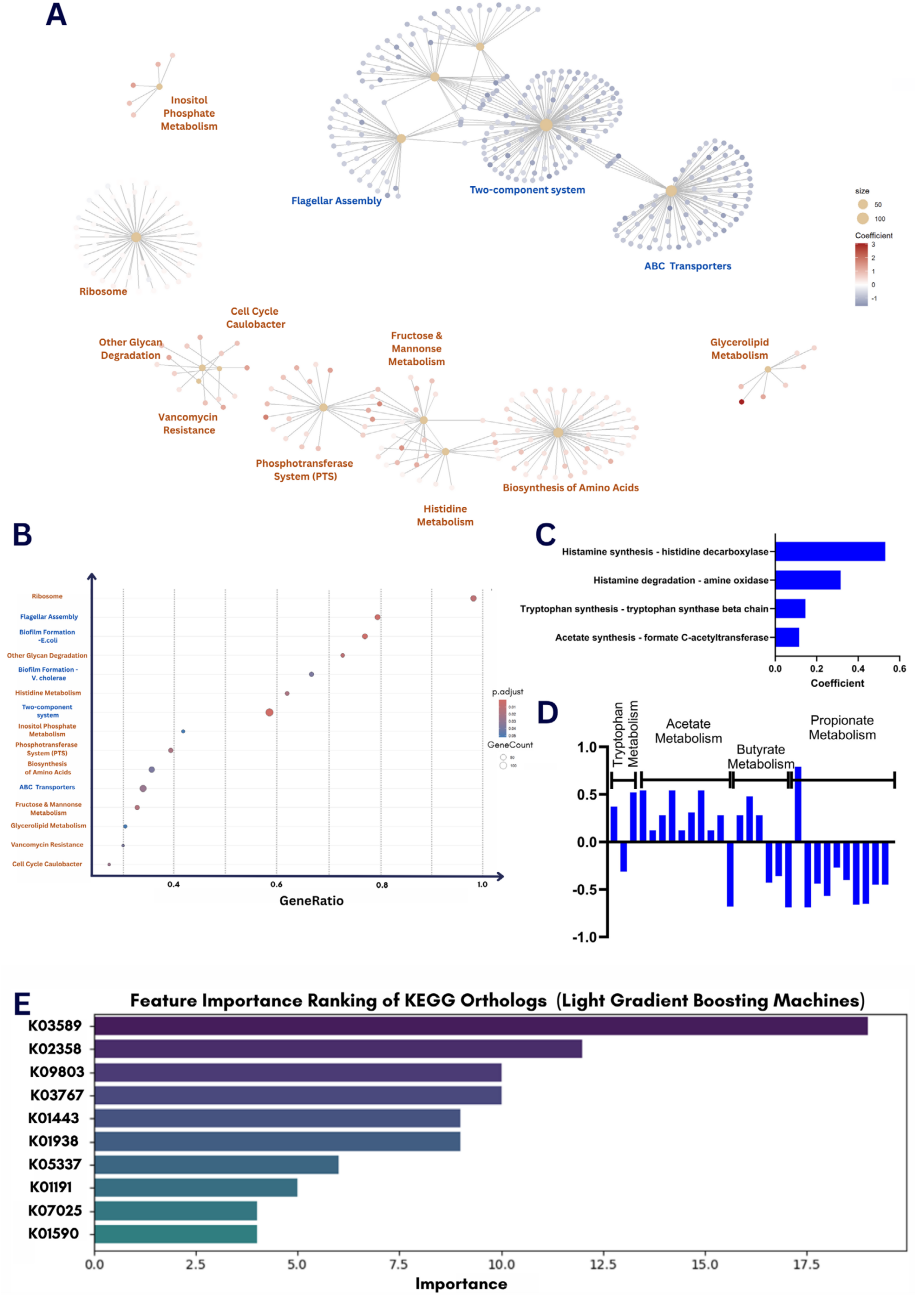
Changes in microbial genes associated with siblings. GSEA plots for significant pathway changes at 12 months of age (A, B). Changes in GBM modules associated with siblings at 6 (C) and 12 (D) months of age. (E) The top 10 features ranked by their contribution to the model's predictive performance, displayed as a horizontal bar chart.

Food allergen sensitization at 12 months of age measured using skin prick tests (SPT) was lower in infants with siblings (4.8% vs. 10.3%; *p* = 0.051), while IgE‐mediated food allergy also tended to be less frequent in infants with siblings (2.7% vs. 6.4%; *p* = 0.080). This trend was maintained out to 2 years of age (SPT: 2.5% vs. 6.8%; food allergy: 0.6% vs. 3.0%). Machine learning methods were used to evaluate if the sibling associated significant differences in 6‐month gene families (*n* = 534; raw *p* < 0.05) were predictive of food sensitization at 12 months of age (Table S7). Among the models evaluated, LightGBM demonstrated high predictive accuracy in identifying food sensitization (ROC 0.96). The top 10 predictive gene families are illustrated in Figure 2e. K01191 (alpha mannosidase important for utilization of dietary and host glycans) and K09803 (unknown function) were frequently among the top 10 features for multiple models (especially logistic regression variants) and should be examined in future studies for effects on immune processes related to allergy development.

Humans have evolved in an environmental and social context that enabled reliable transmission and dispersal of symbionts, accompanied by appropriate nutritional support. While exposure to biodiverse environments is important, human‐adapted symbionts might only be acquired from contact with other humans. Our analysis supports the relevance of close interactions between siblings in shaping the early gut microbiome composition and metabolic functions, with potential important effects on immune development. Compared to pre‐pandemic cohorts, the effect of siblings on gut microbiome development may be more clearly identifiable in this unique CORAL infant birth cohort recruited during pandemic‐enforced social distancing measures, as CORAL infants had reduced exposure to humans outside the home, reduced use of antibiotics, avoided infections, were more frequently breastfed, and had a later acquisition of environmentally transmitted bacteria.

## Author Contributions

Project conceptualization: J.H., L.O., and J.W.; project funding J.H., J.W., and L.O.; study recruitment and allergy clinical assessments: S.H. and J.H.; sample processing and data curation: H.D., A.L., K.K., S.H., N.L., and C.V.; data analysis: H.D., A.L., K.K., N.L., A.A., U.N., C.V., J.W., and L.O.; manuscript writing: H.D., K.K., J.W., J.H., and L.O. All authors contributed to reviewing the manuscript, and all authors agreed to the final version for submission.

## Conflicts of Interest

Carina Venter reports grants from Reckitt and Global parents for eczema research, and contributed to the speakers' bureau for Reckitt, Nestle Nutrition Institute, Danone, and Abbott Nutrition. Liam O'Mahony reports grants from Chiesi, Reckitt, and Fonterra, and participation in the speaker bureau for Nestle, Yakult, Reckitt, and Abbott. Jonathan Hourihane is a board member of the Clemens Von Pirquet Foundation and receives research funding and speaker fees from DBV Technologies, as well as research funding from the City of Dublin Skin and Cancer Hospital Charity and Kenvue. Ujjwal Neogi received travel support from Olink Ab, Sweden. The other authors declare no conflicts of interest.

## Supporting information


**Appendix S1:** all70033‐sup‐0001‐AppendixS1.docx.

## Data Availability

The data that support the findings of this study are openly available in Bioproject at https://www.ncbi.nlm.nih.gov/bioproject?cmd=search, reference number PRJNA1274946.
