# Supplementary material for: Microbial Sharing Between Siblings Supports Metabolic Functions Protective Against Allergy
Source: Allergy. 2025 Aug 29;80(10):2934–7. doi: 10.1111/all.70033 (PMC12486347; doi:10.1111/all.70033)
Supplement: Supplementary file 1 — Appendix S1: all70033‐sup‐0001‐AppendixS1.docx. [file ALL-80-2934-s001.docx]

**Microbial Sharing Between Siblings Supports Metabolic Functions Protective Against Allergy**

Hannah Devotta, Aonghus Lavelle, Katri Korpela, Sadhbh Hurley, Emer Shannon, Nonhlanhla Lunjani, Anoop Ambikan, Ujjwal Neogi, Carina Venter, Jens Walter, Jonathan Hourihane, Liam O’Mahony

**Supplementary Methods.**

*Study Outline*

The CORAL study is a longitudinal prospective observational study of term Irish infants born during the first 3 months of the COVID-19 pandemic, March–May 2020. Exclusion criteria were pre‐birth PCR‐proven SARS‐CoV‐2 infection in a parent or co‐dwelling person, intravenous antibiotics in the neonatal period, multiple births or major congenital anomaly. A total of 351 infants were recruited postnatally to the CORAL Study. Babies had a clinical review along with lateral flow COVID-19 antibody screening at 6 and 12 months of age. Detailed epidemiological information was collected at recruitment and at each 6-month review.

At recruitment and at each 6-month review, families completed an online questionnaire which collected epidemiological information under three main categories. Firstly, the infant’s home environment including household number, presence of siblings, urban or rural location, type of dwelling, childcare outside the home, household pets, and maternal and household smoking status. Secondly, infant health and healthcare utilization including infant weaning, infant illnesses, antibiotic use, SARS-CoV-2 contacts, testing and positivity, infant hospitalization, regular medication use and vaccination status. Lastly information was collected regarding symptoms and signs of allergic conditions including suspected adverse food reactions.

At 12-month and 24-month clinical review, Skin prick testing (SPT) was completed to the 3 most common food allergens (peanut, egg and milk) in all infants. SPT was conducted in line with local and international recommendations and was performed to additional foods only as clinically indicated by history. Sensitization was defined as a positive wheal >3mm. Food allergy at 12 months was defined as sensitization plus a history of immediate reaction or positive oral food challenge (OFC).

Ethical permission was granted by the National COVID-19 Ethics Committee (20-NREC-COV-067). Parent/guardian gave informed consent to participate in the study before taking part.

*Microbiota sampling and sequencing*

Fecal samples were collected at home using a stool sampling kit with preservative (Norgen Biotek), at 6 and 12 months of age. Stabilized samples were returned via post and stored at -80 C until analysis. Microbial DNA was isolated using the DNeasy PowerSoil Pro Kit (Qiagen) and whole-genome metagenomic sequencing was performed by University of Minnesota Genomics Center. All samples were sequenced in one batch. Sequencing files are available under BioProject ID PRJNA1274946 and subject metadata is available at FigShare (DOI 10.6084/m9.figshare.24922716).

*Bioinformatic processing*

Whole-genome shotgun metagenomic libraries were constructed using the Nextera XT protocol with quarter-reaction volumes, generating 704 dual-indexed libraries. Four samples failed to generate sufficient library mass and were excluded from sequencing. The remaining libraries were pooled into two separate pools of 352 samples each and sequenced on an Illumina NovaSeq 6000 platform with S4 flow cells (2 × 150 bp paired-end reads), distributing each pool across two lanes to maximize sequencing depth. Sequencing yielded over 2.25 billion pass-filter reads per lane, exceeding performance specifications. All expected barcodes were detected, except for the low-yield dropout samples. Following sequencing, reads from individual lanes were concatenated by sample identifier, resulting in a single FASTQ file per read direction for each sample. Data quality was high, with all libraries achieving mean quality scores of Q30 or greater.

Host-derived sequences were removed using KneadData with the hg37dec_v0.1 human genome reference, while Trimmomatic was applied for quality trimming, including adapter clipping, base quality filtering, head cropping, sliding window trimming, and enforcing a minimum read length. Bowtie2 was used for alignment, with parallel processing across 7 threads. Intermediate and host-mapped reads were discarded to optimize storage, and the decontaminated reads were compressed for downstream analysis.

Functional and taxonomic profiling was performed using HUMAnN3 and MetaPhlAn4, with HUMAnN3 enabling functional annotation through MetaPhlAn-based taxonomic assignment, nucleotide-level mapping with the ChocoPhlAn database, and protein-level annotation using UniRef90. The processed inputs were further analysed using supporting utilities from HUMAnN3- namely humann_regroup_table, humann_renorm_table, and humann_join_tables. 15 computational threads were used for parallel analysis, with a custom Bash pipeline to streamline quality control, functional profiling, and the data management workflow.

*Statistical analysis*

The statistical analyses were conducted in R. Principal coordinates analysis (function capscale in R package vegan) using Pearson correlation distances was used to depict the overall gut microbiota composition and its determinants. Both marginal and sequential PERMANOVA analyses were performed using the adonis2 function from the vegan package in R to adjust for confounding exposures such as breastfeeding, birth mode, or external environmental factors. The latter was represented by a composite exposure score incorporating multiple environmental and household characteristics: presence of pets, number of family members, occupation of household members (including essential worker status), daycare attendance, and type of residence (house versus apartment). The marginal PERMANOVA evaluated the association of each predictor individually with microbial taxa or gene families, whereas sequential PERMANOVA assessed the effect of each variable after adjusting for previously included terms. Gene Set Enrichment Analysis (GSEA) was performed using the clusterProfiler R package. KEGG Orthologs (KOs) were derived from the gene family output of HUMAnN3 and normalized prior to analysis. Differential abundance analysis was conducted using MaAsLin2, and genes were ranked by model coefficients. KO terms were mapped to KEGG pathways, which were used as input for pre-ranked GSEA. All pathways were assessed for enrichment, and statistical significance was determined based on adjusted p-values.

Several machine learning models were evaluated to develop a food sensitisation prediction model based on the 6-month gene family data. Logistic regression with regularisation approaches including lasso, ridge, and elastic nets were applied to manage multicollinearity and perform feature selection using L1 and L2 regularization. Tree-based ensemble methods (XGBoost, Random Forest, LightGBM) were utilised to capture non-linear associations and interactions among features, while support vector machines (SVM) addressed complex classification boundaries.

All codes used for statistical analysis are available at https://github.com/shdalgo/CORAL-Siblings.

Supplementary Figure S1. Alpha diversity at 6 and 12 months of age.


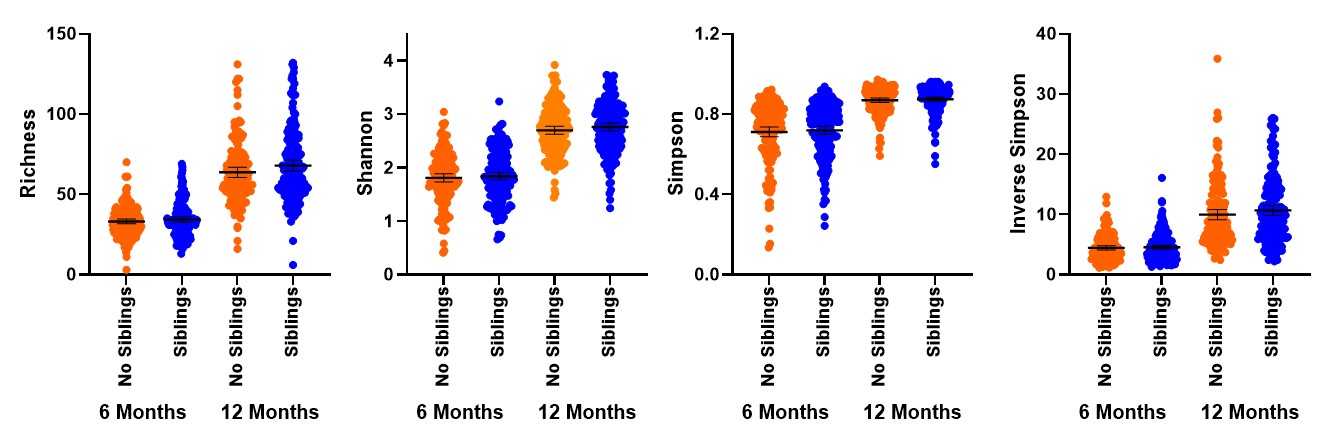


Supplementary Figure S2. Gene family differences at 6 and 12-months-of-age.

6 months - Bray Curtis, p-value = 0.1​08

 
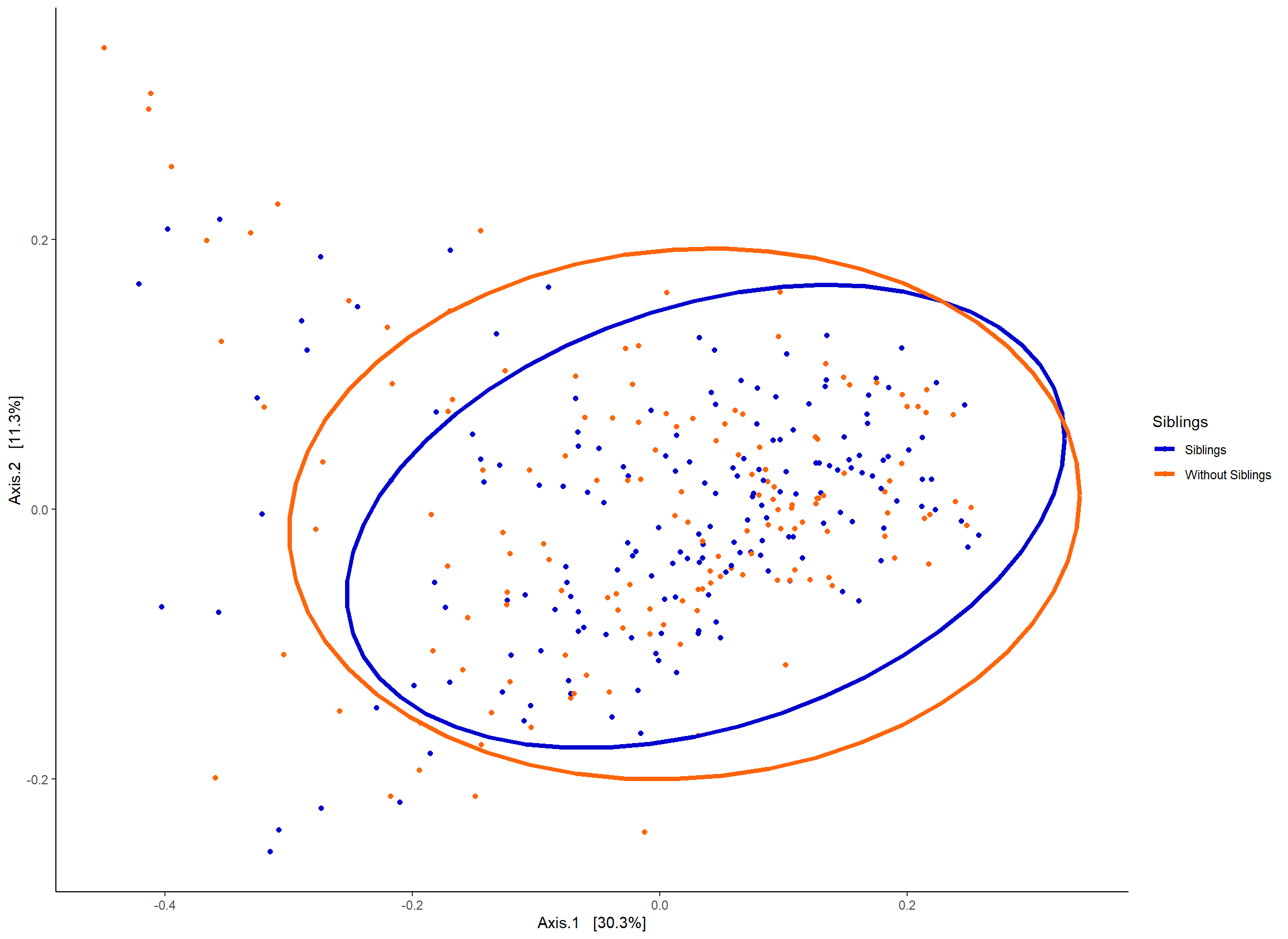


12 months - Bray Curtis, p-value = 0.006

 
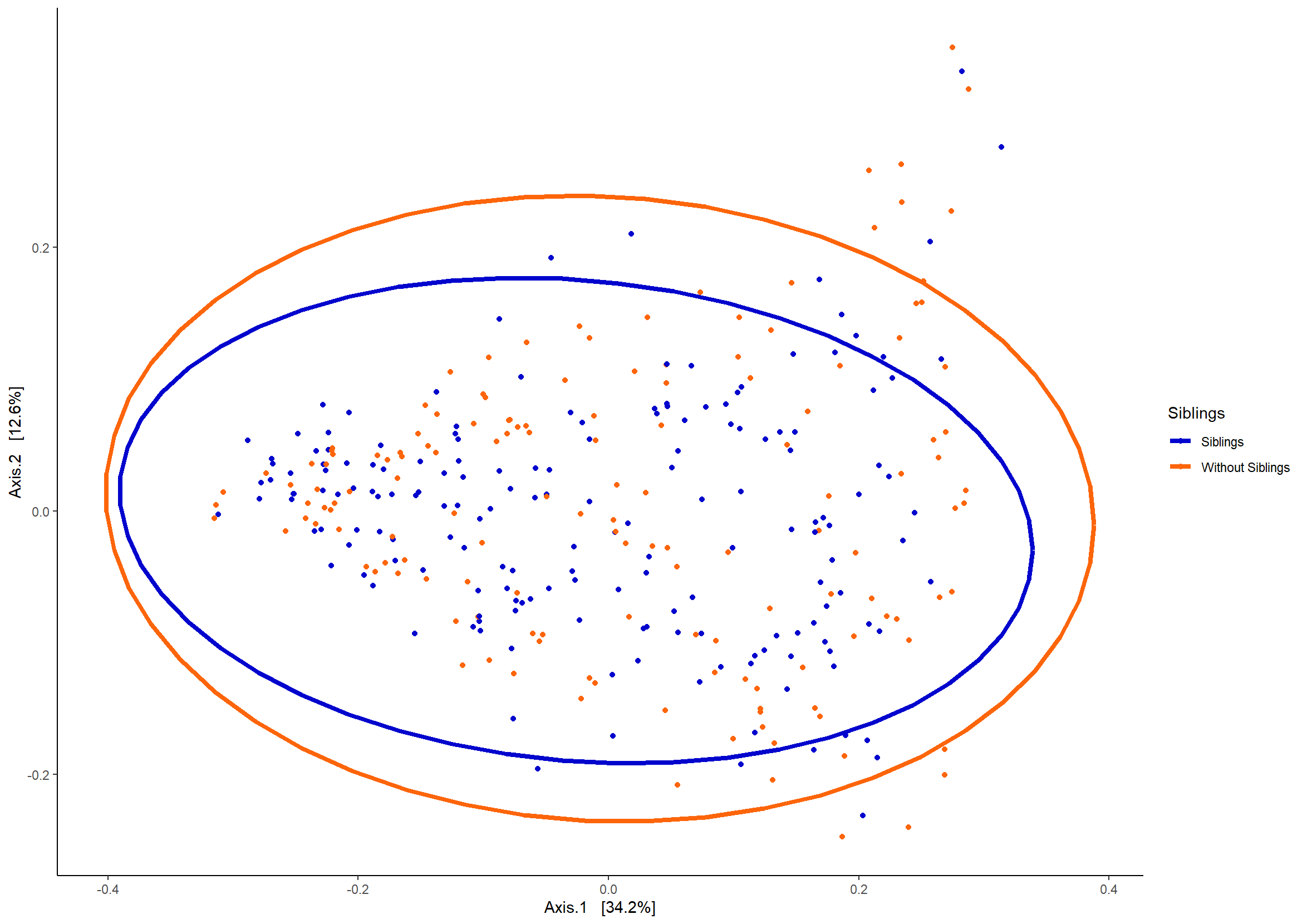


Supplementary Figure S3. GSEA differences at 6 months-of-age.


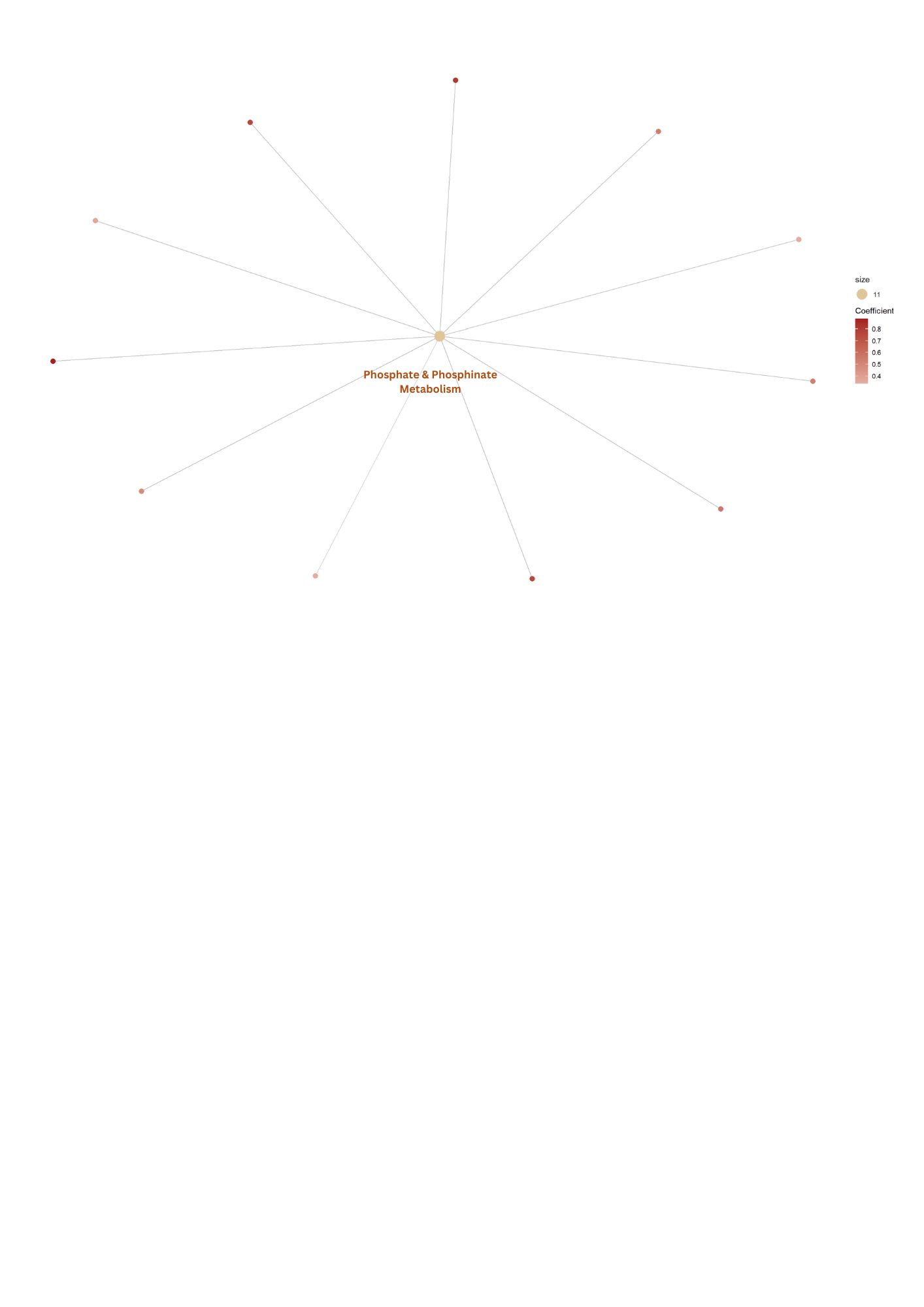


Supplementary Table S1. CORAL infant cohort metadata.

|  | **6 Months (n=351)** | |  | **12 Months (n=343)** | | |  | |
| --- | --- | --- | --- | --- | --- | --- | --- | --- |
|  | No Siblings (n=164) | Siblings (n=187) | p-value | | No Siblings (n=156) | Siblings (n=187) | p-value |  |
| **C section** | 59 – 36% | 60 – 32% | 0.44 |  | |  |  |  |
| **Male:Female**  **Antibiotics** | 92:72  9 – 6% | 99:88  11 – 6% | 0.55  0.87 | 89:67  28 – 18% | | 99:88  39 – 21% | 0.45  0.50 |  |
| **Pets** | 46 – 28% | 62 – 33% | 0.33 |  | |  |  |  |
| **Rural:Urban Home** | 20:144 | 30:157 | 0.30 |  | |  |  |  |
| **Infant in Nursery/Creche** | 0 | 0 | / | 34 – 22% | | 38 – 20% | 0.74 |  |
| **Childminder in Infant’s Home** | 8 – 5% | 10 – 5% | 0.84 | 28 -18% | | 29 – 16% | 0.55 |  |
| **Childminder in Childminder’s Home** | 0 | 0 | / | 39 – 25% | | 41 – 22% | 0.50 |  |
| **Smoking in the Home** | 11 – 7% | 14 – 7% | 0.78 |  | |  |  |  |
| **Exclusive Breast Feeding (BF)** | 64 – 39% | 81 – 43% | 0.42 | 34 – 22% | | 47 – 25% | 0.47 |  |
| **Both BF & Formula** | 24 – 15% | 27 – 14% | 0.96 | 17 – 11% | | 23 – 12% | 0.69 |  |
| **Formula** | 72 – 44% | 77 – 41% | 0.61 | 69 – 44% | | 72 – 39% | 0.28 |  |
| **Other** | 4 – 2.4% | 2 – 1.1% | 0.32 | 36 – 23% | | 45 – 24% | 0.83 |  |
|  |  |  |  |  | |  |  | |

Supplementary Table S2. Taxa differences associated with siblings at 6 and 12 months.

| **6 Months Taxa** | | **Coefficent** | | **SD** | | **P raw** | | **P FDR adjusted** | |
| --- | --- | --- | --- | --- | --- | --- | --- | --- | --- |
| Bifidobacterium_bifidum | | 1.383732 | | 0.333248 | | 4.16E-05 | | 0.001166 | |
| Eggerthella_lenta | | 0.986931 | | 0.258268 | | 0.000158 | | 0.002944 | |
| Bifidobacterium_pseudocatenulatum | | 1.291102 | | 0.34182 | | 0.000187 | | 0.003309 | |
| Bifidobacterium_dentium | | -0.91531 | | 0.297192 | | 0.002239 | | 0.02508 | |
| Bifidobacterium_breve | | 0.706955 | | 0.238562 | | 0.003256 | | 0.033909 | |
| Clostridium_paraputrificum | | -0.56175 | | 0.195316 | | 0.004279 | | 0.038038 | |
| Bifidobacterium_longum | | 0.574468 | | 0.212013 | | 0.007077 | | 0.041692 | |
| Bacteroides_fragilis | | 0.881274 | | 0.331649 | | 0.008249 | | 0.047742 | |
| **12 Months Taxa** | **Coefficent** | | **SD** | | **P raw** | | **P FDR adjusted** | |  |
| Bifidobacterium_pseudocatenulatum | 1.902984 | | 0.304867 | | 1.34E-09 | | 3.94E-07 | |  |
| Clostridium_butyricum | -1.13188 | | 0.198966 | | 2.85E-08 | | 5.97E-06 | |  |
| Alistipes_finegoldii | 1.143716 | | 0.209268 | | 9.20E-08 | | 1.69E-05 | |  |
| Veillonella_rogosae | -0.99522 | | 0.184568 | | 1.34E-07 | | 2.18E-05 | |  |
| Erysipelatoclostridium_ramosum | -1.04597 | | 0.199432 | | 2.82E-07 | | 4.14E-05 | |  |
| Veillonella_parvula | -1.47475 | | 0.300043 | | 1.41E-06 | | 0.000172 | |  |
| Evtepia_gabavorous | 0.505945 | | 0.104269 | | 1.89E-06 | | 0.000214 | |  |
| Alistipes_onderdonkii | 1.349264 | | 0.281875 | | 2.57E-06 | | 0.000252 | |  |
| Escherichia_coli | -0.98683 | | 0.208625 | | 3.35E-06 | | 0.000307 | |  |
| Ruminococcus_gnavus | -0.77262 | | 0.169811 | | 7.58E-06 | | 0.000654 | |  |
| Megasphaera_micronuciformis | -0.83571 | | 0.191788 | | 1.77E-05 | | 0.001363 | |  |
| Anaerostipes_hadrus | 1.319495 | | 0.306391 | | 2.20E-05 | | 0.001465 | |  |
| Tyzzerella_nexilis | -1.04862 | | 0.243065 | | 2.13E-05 | | 0.001465 | |  |
| Lacrimispora_amygdalina | 1.026291 | | 0.248953 | | 4.76E-05 | | 0.00291 | |  |
| Bacteroides_fragilis | 1.205065 | | 0.310053 | | 0.000123 | | 0.006229 | |  |
| Bifidobacterium_bifidum | 1.132161 | | 0.301633 | | 0.000206 | | 0.009178 | |  |
| Bacteroides_ovatus | 1.120282 | | 0.300345 | | 0.000226 | | 0.009742 | |  |
| Faecalibacterium_prausnitzii | 1.34163 | | 0.361497 | | 0.000242 | | 0.010159 | |  |
| Gemmiger_formicilis | 0.844393 | | 0.228428 | | 0.000256 | | 0.010445 | |  |
| Dorea_formicigenerans | 0.520821 | | 0.141219 | | 0.000265 | | 0.010494 | |  |
| Dorea_longicatena | 0.831356 | | 0.230497 | | 0.000359 | | 0.013842 | |  |
| Streptococcus_thermophilus | -0.71082 | | 0.20446 | | 0.000577 | | 0.018798 | |  |
| Blautia_luti | 0.455494 | | 0.132364 | | 0.000655 | | 0.020873 | |  |
| Blautia_hansenii | -0.72548 | | 0.213277 | | 0.000753 | | 0.022127 | |  |
| Hydrogeniiclostidium_mannosilyticum | 0.354155 | | 0.104884 | | 0.000823 | | 0.023205 | |  |
| Faecalimonas_umbilicata | -0.87806 | | 0.262733 | | 0.000928 | | 0.024321 | |  |
| Clostridia_bacterium_UC5_1_1D1 | 0.349298 | | 0.105669 | | 0.001053 | | 0.026424 | |  |
| Dysosmobacter_welbionis | 0.585205 | | 0.178594 | | 0.001163 | | 0.027975 | |  |
| Bifidobacterium_animalis | -0.52817 | | 0.161765 | | 0.001211 | | 0.028193 | |  |
| Prevotella_buccae | -0.59211 | | 0.181293 | | 0.001207 | | 0.028193 | |  |
| Clostridium_SGB4750 | 0.555408 | | 0.180643 | | 0.002286 | | 0.031916 | |  |
| Faecalibacterium_SGB15346 | 0.519255 | | 0.167305 | | 0.002079 | | 0.031916 | |  |
| Lacticaseibacillus_rhamnosus | -0.69517 | | 0.22448 | | 0.002126 | | 0.031916 | |  |
| Bifidobacterium_dentium | -0.71802 | | 0.232472 | | 0.002184 | | 0.031916 | |  |
| Bifidobacterium_catenulatum | 0.866908 | | 0.28794 | | 0.00281 | | 0.037929 | |  |
| Ruminococcus_torques | 0.933874 | | 0.313447 | | 0.003105 | | 0.042362 | |  |
| Enterocloster_clostridioformis | -0.44673 | | 0.150797 | | 0.003277 | | 0.044621 | |  |
| Parasutterella_excrementihominis | 0.370974 | | 0.132121 | | 0.005288 | | 0.046805 | |  |
| Bifidobacterium_longum | 0.424768 | | 0.152268 | | 0.005588 | | 0.049981 | |  |

Supplementary Table S3. Gene family differences associated with siblings at 6 months.

| **KEGG** | Coefficent | SD | P raw | P FDR adjusted |
| --- | --- | --- | --- | --- |
| **K07272** | 2.59 | 0.53 | 1.75E-06 | 0.007028 |
| **K07025** | 2.00 | 0.46 | 1.74E-05 | 0.034969 |
| **K00001** | 1.56 | 0.37 | 3.17E-05 | 0.038513 |
| **K01224** | 1.50 | 0.36 | 4.79E-05 | 0.038513 |
| **K09803** | 2.44 | 0.58 | 4.21E-05 | 0.038513 |
| **K00355** | 2.07 | 0.53 | 0.000126 | 0.067082 |
| **K06956** | 1.63 | 0.42 | 0.000115 | 0.067082 |
| **K12308** | 0.82 | 0.21 | 0.000134 | 0.067082 |
| **K01191** | 1.57 | 0.41 | 0.000199 | 0.088641 |
| **K00030** | 0.93 | 0.25 | 0.00029 | 0.094565 |
| **K00880** | 1.80 | 0.51 | 0.000541 | 0.094565 |
| **K01512** | 0.96 | 0.27 | 0.000514 | 0.094565 |
| **K01902** | 0.91 | 0.26 | 0.000497 | 0.094565 |
| **K02037** | 0.60 | 0.17 | 0.000522 | 0.094565 |
| **K02291** | 0.89 | 0.25 | 0.000431 | 0.094565 |
| **K03727** | 1.51 | 0.43 | 0.00053 | 0.094565 |
| **K07062** | 1.55 | 0.43 | 0.000387 | 0.094565 |
| **K07442** | 1.50 | 0.42 | 0.000375 | 0.094565 |
| **K07458** | 1.20 | 0.33 | 0.000313 | 0.094565 |
| **K07473** | 1.11 | 0.31 | 0.000459 | 0.094565 |
| **K08177** | 1.18 | 0.32 | 0.000237 | 0.094565 |
| **K10007** | 1.55 | 0.42 | 0.000284 | 0.094565 |
| **K13059** | 1.54 | 0.43 | 0.000443 | 0.094565 |
| **K21885** | 1.68 | 0.48 | 0.000574 | 0.096048 |
| **K10117** | 0.67 | 0.19 | 0.000638 | 0.102535 |
| **K02198** | 0.87 | 0.26 | 0.00077 | 0.117789 |
| **K08744** | 1.45 | 0.43 | 0.000792 | 0.117789 |
| **K00018** | 1.52 | 0.45 | 0.000901 | 0.117852 |
| **K01129** | 0.69 | 0.20 | 0.000855 | 0.117852 |
| **K03517** | 0.81 | 0.24 | 0.000869 | 0.117852 |
| **K03688** | 0.93 | 0.28 | 0.000911 | 0.117852 |
| **K10773** | 0.49 | 0.15 | 0.000939 | 0.117852 |
| **K01779** | 1.89 | 0.57 | 0.001013 | 0.123243 |
| **K09974** | 1.13 | 0.34 | 0.001043 | 0.123243 |
| **K09704** | 1.22 | 0.37 | 0.001082 | 0.124165 |
| **K02026** | 0.58 | 0.18 | 0.001196 | 0.124832 |
| **K03789** | 1.04 | 0.31 | 0.001119 | 0.124832 |
| **K04749** | 0.74 | 0.22 | 0.001178 | 0.124832 |
| **K07009** | 0.93 | 0.28 | 0.001212 | 0.124832 |
| **K01182** | 1.48 | 0.45 | 0.001304 | 0.127818 |
| **K07816** | 1.41 | 0.43 | 0.001275 | 0.127818 |
| **K01903** | 0.72 | 0.22 | 0.001458 | 0.132165 |
| **K03502** | 0.98 | 0.30 | 0.00148 | 0.132165 |
| **K03529** | 1.11 | 0.34 | 0.001389 | 0.132165 |
| **K03655** | 0.46 | 0.14 | 0.001463 | 0.132165 |
| **K00847** | 0.74 | 0.23 | 0.001713 | 0.132338 |
| **K00881** | -1.26 | 0.40 | 0.001706 | 0.132338 |
| **K01256** | 0.94 | 0.29 | 0.001651 | 0.132338 |
| **K02469** | 0.93 | 0.29 | 0.001713 | 0.132338 |
| **K02564** | 0.43 | 0.13 | 0.001564 | 0.132338 |
| **K06024** | 0.84 | 0.26 | 0.001616 | 0.132338 |
| **K13940** | 1.81 | 0.57 | 0.001651 | 0.132338 |
| **K07474** | -1.02 | 0.32 | 0.001774 | 0.134464 |
| **K02025** | 0.50 | 0.16 | 0.001816 | 0.135104 |
| **K01243** | 0.41 | 0.13 | 0.001924 | 0.140593 |
| **K00613** | 0.91 | 0.29 | 0.002015 | 0.144596 |
| **K03738** | 0.78 | 0.25 | 0.002111 | 0.148804 |
| **K00088** | 0.61 | 0.20 | 0.002552 | 0.155799 |
| **K00185** | 0.87 | 0.29 | 0.002816 | 0.155799 |
| **K00620** | 0.75 | 0.24 | 0.002565 | 0.155799 |
| **K00989** | 0.72 | 0.24 | 0.002432 | 0.155799 |
| **K01421** | 1.14 | 0.38 | 0.002758 | 0.155799 |
| **K01733** | 0.63 | 0.21 | 0.002656 | 0.155799 |
| **K02074** | 1.69 | 0.56 | 0.002749 | 0.155799 |
| **K02501** | 0.67 | 0.22 | 0.002512 | 0.155799 |
| **K02601** | 0.36 | 0.12 | 0.002831 | 0.155799 |
| **K02790** | -0.92 | 0.30 | 0.002685 | 0.155799 |
| **K02791** | -0.92 | 0.30 | 0.002685 | 0.155799 |
| **K03101** | 0.40 | 0.13 | 0.00248 | 0.155799 |
| **K03424** | 0.40 | 0.13 | 0.002464 | 0.155799 |
| **K03503** | 1.07 | 0.35 | 0.002737 | 0.155799 |
| **K06117** | 1.66 | 0.54 | 0.002268 | 0.155799 |
| **K22397** | 1.56 | 0.51 | 0.002736 | 0.155799 |
| **K00666** | 0.93 | 0.31 | 0.003102 | 0.166158 |
| **K05350** | 1.54 | 0.52 | 0.003091 | 0.166158 |
| **K18120** | -1.11 | 0.37 | 0.003166 | 0.167374 |
| **K00180** | 0.78 | 0.26 | 0.003444 | 0.168472 |
| **K00183** | 0.89 | 0.31 | 0.004343 | 0.168472 |
| **K00982** | 0.87 | 0.30 | 0.004471 | 0.168472 |
| **K01079** | 0.99 | 0.34 | 0.004162 | 0.168472 |
| **K01354** | 1.31 | 0.46 | 0.004387 | 0.168472 |
| **K01520** | 0.37 | 0.13 | 0.004207 | 0.168472 |
| **K01590** | 1.21 | 0.42 | 0.004068 | 0.168472 |
| **K01869** | 0.28 | 0.10 | 0.005199 | 0.168472 |
| **K01990** | 0.35 | 0.12 | 0.004091 | 0.168472 |
| **K02027** | 0.66 | 0.23 | 0.004893 | 0.168472 |
| **K02282** | 0.65 | 0.23 | 0.004477 | 0.168472 |
| **K02314** | 0.89 | 0.30 | 0.003818 | 0.168472 |
| **K02654** | 1.02 | 0.35 | 0.004201 | 0.168472 |
| **K03657** | 0.34 | 0.12 | 0.003628 | 0.168472 |
| **K03740** | 0.83 | 0.29 | 0.004524 | 0.168472 |
| **K03799** | 0.42 | 0.15 | 0.004367 | 0.168472 |
| **K04567** | 0.30 | 0.11 | 0.004999 | 0.168472 |
| **K06178** | 0.62 | 0.21 | 0.003433 | 0.168472 |
| **K06191** | 1.06 | 0.38 | 0.005137 | 0.168472 |
| **K06217** | 0.38 | 0.13 | 0.004475 | 0.168472 |
| **K06330** | 1.49 | 0.52 | 0.004255 | 0.168472 |
| **K06442** | 1.39 | 0.49 | 0.00478 | 0.168472 |
| **K06902** | 1.06 | 0.38 | 0.005027 | 0.168472 |
| **K07315** | 0.68 | 0.24 | 0.004452 | 0.168472 |
| **K07447** | 0.32 | 0.11 | 0.003406 | 0.168472 |
| **K07478** | 0.38 | 0.13 | 0.004746 | 0.168472 |
| **K07574** | 0.62 | 0.22 | 0.004758 | 0.168472 |
| **K08156** | 1.46 | 0.50 | 0.003703 | 0.168472 |
| **K08296** | 0.70 | 0.25 | 0.004866 | 0.168472 |
| **K08369** | 0.83 | 0.29 | 0.004544 | 0.168472 |
| **K08659** | 0.93 | 0.32 | 0.004505 | 0.168472 |
| **K08681** | 0.81 | 0.28 | 0.003549 | 0.168472 |
| **K08972** | 0.91 | 0.32 | 0.005154 | 0.168472 |
| **K09118** | 0.90 | 0.30 | 0.003321 | 0.168472 |
| **K09903** | 0.27 | 0.09 | 0.004861 | 0.168472 |
| **K10190** | 1.56 | 0.55 | 0.004961 | 0.168472 |
| **K10805** | 0.59 | 0.21 | 0.005196 | 0.168472 |
| **K11529** | 0.93 | 0.32 | 0.004391 | 0.168472 |
| **K13527** | 0.80 | 0.28 | 0.004659 | 0.168472 |
| **K13829** | 1.09 | 0.39 | 0.005186 | 0.168472 |
| **K15921** | 1.68 | 0.57 | 0.003385 | 0.168472 |
| **K17810** | -1.16 | 0.40 | 0.004415 | 0.168472 |
| **K18334** | 1.61 | 0.56 | 0.004449 | 0.168472 |
| **K19265** | 0.68 | 0.24 | 0.004561 | 0.168472 |
| **K20859** | 0.75 | 0.26 | 0.004296 | 0.168472 |
| **K21011** | 1.47 | 0.52 | 0.004797 | 0.168472 |
| **K21993** | 0.43 | 0.15 | 0.004731 | 0.168472 |
| **K22606** | 0.89 | 0.30 | 0.003401 | 0.168472 |
| **K02503** | 0.97 | 0.34 | 0.005252 | 0.168817 |
| **K20814** | 1.58 | 0.56 | 0.005349 | 0.170581 |
| **K12111** | 1.36 | 0.49 | 0.005563 | 0.175997 |
| **K00075** | 0.45 | 0.16 | 0.00561 | 0.176115 |
| **K00526** | 0.64 | 0.23 | 0.006172 | 0.177739 |
| **K00688** | 0.55 | 0.20 | 0.005978 | 0.177739 |
| **K00951** | 0.58 | 0.21 | 0.005869 | 0.177739 |
| **K01624** | 0.59 | 0.21 | 0.005883 | 0.177739 |
| **K01870** | 0.41 | 0.15 | 0.006221 | 0.177739 |
| **K01873** | 0.33 | 0.12 | 0.006237 | 0.177739 |
| **K02652** | 0.85 | 0.31 | 0.006172 | 0.177739 |
| **K03147** | 0.45 | 0.16 | 0.006002 | 0.177739 |
| **K03767** | 0.57 | 0.20 | 0.005836 | 0.177739 |
| **K06180** | 0.51 | 0.18 | 0.006237 | 0.177739 |
| **K07133** | 0.59 | 0.21 | 0.00599 | 0.177739 |
| **K08484** | -0.75 | 0.27 | 0.006122 | 0.177739 |
| **K13570** | 1.28 | 0.46 | 0.006077 | 0.177739 |
| **K11928** | 0.52 | 0.19 | 0.006412 | 0.181443 |
| **K09761** | 0.53 | 0.19 | 0.006517 | 0.183115 |
| **K02071** | 0.34 | 0.13 | 0.006591 | 0.183422 |
| **K03530** | 0.25 | 0.09 | 0.006619 | 0.183422 |
| **K01524** | 0.57 | 0.21 | 0.00672 | 0.183669 |
| **K20116** | 0.52 | 0.19 | 0.006711 | 0.183669 |
| **K00177** | 0.86 | 0.32 | 0.006863 | 0.183832 |
| **K03686** | 0.40 | 0.15 | 0.006862 | 0.183832 |
| **K04517** | 1.08 | 0.40 | 0.006818 | 0.183832 |
| **K01923** | 0.26 | 0.10 | 0.006922 | 0.184191 |
| **K00184** | 0.97 | 0.36 | 0.007537 | 0.190971 |
| **K00674** | 0.46 | 0.17 | 0.00753 | 0.190971 |
| **K00690** | 0.78 | 0.29 | 0.007537 | 0.190971 |
| **K00835** | -0.78 | 0.29 | 0.0077 | 0.190971 |
| **K01081** | 1.34 | 0.50 | 0.007312 | 0.190971 |
| **K01151** | 0.49 | 0.18 | 0.007594 | 0.190971 |
| **K02821** | 1.05 | 0.39 | 0.007225 | 0.190971 |
| **K03322** | 0.55 | 0.20 | 0.007717 | 0.190971 |
| **K03724** | 1.13 | 0.42 | 0.007636 | 0.190971 |
| **K07460** | 0.44 | 0.16 | 0.007647 | 0.190971 |
| **K20117** | 0.54 | 0.20 | 0.007747 | 0.190971 |
| **K20118** | 0.54 | 0.20 | 0.007747 | 0.190971 |
| **K01689** | 0.23 | 0.09 | 0.007899 | 0.192364 |
| **K02316** | 0.36 | 0.14 | 0.007888 | 0.192364 |
| **K01938** | 0.45 | 0.17 | 0.007953 | 0.192499 |
| **K00053** | 0.36 | 0.13 | 0.008127 | 0.193219 |
| **K01482** | 0.70 | 0.26 | 0.008114 | 0.193219 |
| **K01736** | 0.31 | 0.12 | 0.0081 | 0.193219 |
| **K01939** | 0.26 | 0.10 | 0.008281 | 0.195328 |
| **K03110** | 0.35 | 0.13 | 0.008313 | 0.195328 |
| **K00831** | 0.33 | 0.13 | 0.008631 | 0.196058 |
| **K01443** | 0.73 | 0.28 | 0.008668 | 0.196058 |
| **K01803** | 0.18 | 0.07 | 0.008405 | 0.196058 |
| **K01929** | 0.52 | 0.20 | 0.008525 | 0.196058 |
| **K02931** | 0.24 | 0.09 | 0.008686 | 0.196058 |
| **K11263** | -1.01 | 0.38 | 0.008606 | 0.196058 |
| **K18013** | -0.64 | 0.24 | 0.00856 | 0.196058 |
| **K02038** | 0.30 | 0.11 | 0.008763 | 0.1967 |
| **K04078** | 0.26 | 0.10 | 0.009115 | 0.203466 |
| **K01278** | 0.92 | 0.35 | 0.009305 | 0.204306 |
| **K01889** | 0.26 | 0.10 | 0.009294 | 0.204306 |
| **K07177** | 1.40 | 0.53 | 0.009209 | 0.204306 |
| **K03466** | 0.37 | 0.14 | 0.009387 | 0.204309 |
| **K12291** | 1.04 | 0.40 | 0.009407 | 0.204309 |
| **K02337** | 0.47 | 0.18 | 0.009533 | 0.204924 |
| **K02945** | 0.27 | 0.10 | 0.009555 | 0.204924 |
| **K07771** | -1.00 | 0.38 | 0.009588 | 0.204924 |
| **K03647** | 0.76 | 0.29 | 0.009711 | 0.206444 |
| **K01937** | 0.25 | 0.09 | 0.009806 | 0.207367 |
| **K02341** | 0.49 | 0.19 | 0.009901 | 0.208286 |
| **K01867** | 0.33 | 0.13 | 0.010011 | 0.208415 |
| **K06925** | -0.64 | 0.25 | 0.009962 | 0.208415 |
| **K01439** | 0.57 | 0.22 | 0.010068 | 0.208516 |
| **K01739** | 0.43 | 0.17 | 0.010396 | 0.208905 |
| **K01933** | 0.30 | 0.11 | 0.010236 | 0.208905 |
| **K02863** | 0.25 | 0.10 | 0.010398 | 0.208905 |
| **K03587** | 0.89 | 0.34 | 0.010381 | 0.208905 |
| **K09013** | 0.40 | 0.16 | 0.010239 | 0.208905 |
| **K22306** | -1.28 | 0.50 | 0.010347 | 0.208905 |
| **K00560** | 0.34 | 0.13 | 0.01058 | 0.211499 |
| **K02434** | 0.48 | 0.19 | 0.010704 | 0.212922 |
| **K03625** | 0.38 | 0.15 | 0.010858 | 0.214906 |
| **K07045** | 0.95 | 0.37 | 0.010919 | 0.215069 |
| **K01693** | 0.46 | 0.18 | 0.011298 | 0.219309 |
| **K02435** | 0.40 | 0.16 | 0.011255 | 0.219309 |
| **K09703** | 0.61 | 0.24 | 0.011234 | 0.219309 |
| **K03624** | 0.37 | 0.15 | 0.011459 | 0.220306 |
| **K05351** | 1.46 | 0.57 | 0.011445 | 0.220306 |
| **K01880** | 0.36 | 0.14 | 0.011776 | 0.220821 |
| **K02055** | -0.77 | 0.30 | 0.011871 | 0.220821 |
| **K02500** | 0.22 | 0.09 | 0.011912 | 0.220821 |
| **K02860** | 0.35 | 0.14 | 0.01188 | 0.220821 |
| **K03217** | 0.38 | 0.15 | 0.011679 | 0.220821 |
| **K03593** | 0.90 | 0.36 | 0.011926 | 0.220821 |
| **K15771** | 1.35 | 0.53 | 0.011697 | 0.220821 |
| **K16329** | -0.82 | 0.32 | 0.0118 | 0.220821 |
| **K01239** | 1.45 | 0.57 | 0.012135 | 0.222728 |
| **K02988** | 0.21 | 0.08 | 0.01214 | 0.222728 |
| **K03695** | 0.33 | 0.13 | 0.012275 | 0.22402 |
| **K09138** | 0.61 | 0.24 | 0.012322 | 0.22402 |
| **K00895** | -0.41 | 0.16 | 0.012422 | 0.224823 |
| **K08289** | 0.50 | 0.20 | 0.01249 | 0.225039 |
| **K00525** | 0.48 | 0.19 | 0.012824 | 0.225108 |
| **K00767** | 0.52 | 0.21 | 0.01283 | 0.225108 |
| **K01491** | 0.28 | 0.11 | 0.012691 | 0.225108 |
| **K01955** | 0.21 | 0.08 | 0.012827 | 0.225108 |
| **K06921** | 1.46 | 0.58 | 0.012564 | 0.225108 |
| **K09954** | 1.00 | 0.40 | 0.012607 | 0.225108 |
| **K00395** | 0.59 | 0.24 | 0.013141 | 0.226829 |
| **K01494** | 0.44 | 0.18 | 0.013105 | 0.226829 |
| **K01885** | 0.30 | 0.12 | 0.013257 | 0.226829 |
| **K03703** | 0.35 | 0.14 | 0.013027 | 0.226829 |
| **K14061** | -0.87 | 0.35 | 0.013167 | 0.226829 |
| **K18955** | 0.61 | 0.24 | 0.013267 | 0.226829 |
| **K00930** | 0.31 | 0.13 | 0.013622 | 0.228384 |
| **K01626** | 0.33 | 0.13 | 0.013698 | 0.228384 |
| **K02050** | 0.90 | 0.36 | 0.013671 | 0.228384 |
| **K03149** | 0.27 | 0.11 | 0.013683 | 0.228384 |
| **K04488** | 0.29 | 0.12 | 0.013561 | 0.228384 |
| **K08281** | 0.83 | 0.33 | 0.013584 | 0.228384 |
| **K05839** | 1.01 | 0.41 | 0.01387 | 0.230288 |
| **K00287** | 0.60 | 0.24 | 0.01398 | 0.230489 |
| **K01696** | 0.36 | 0.14 | 0.013997 | 0.230489 |
| **K21600** | 1.02 | 0.41 | 0.014173 | 0.232431 |
| **K18205** | 1.17 | 0.47 | 0.014234 | 0.232485 |
| **K00821** | 0.42 | 0.17 | 0.014336 | 0.233199 |
| **K02986** | 0.14 | 0.06 | 0.014452 | 0.234144 |
| **K17363** | 0.53 | 0.21 | 0.014552 | 0.234823 |
| **K02358** | 0.13 | 0.05 | 0.014766 | 0.237323 |
| **K00215** | 0.33 | 0.13 | 0.014832 | 0.237436 |
| **K00425** | -0.61 | 0.25 | 0.015046 | 0.239439 |
| **K00700** | 0.31 | 0.13 | 0.01554 | 0.239439 |
| **K00975** | 0.29 | 0.12 | 0.015406 | 0.239439 |
| **K01714** | 0.25 | 0.10 | 0.015211 | 0.239439 |
| **K02035** | 0.44 | 0.18 | 0.015473 | 0.239439 |
| **K02948** | 0.13 | 0.05 | 0.015578 | 0.239439 |
| **K03544** | 0.24 | 0.10 | 0.015709 | 0.239439 |
| **K03687** | 0.32 | 0.13 | 0.015842 | 0.239439 |
| **K04021** | -0.94 | 0.39 | 0.015333 | 0.239439 |
| **K04077** | 0.16 | 0.07 | 0.015851 | 0.239439 |
| **K07309** | 0.85 | 0.35 | 0.015742 | 0.239439 |
| **K07652** | -0.87 | 0.36 | 0.015777 | 0.239439 |
| **K09477** | -0.78 | 0.32 | 0.015349 | 0.239439 |
| **K12941** | 1.09 | 0.45 | 0.015407 | 0.239439 |
| **K13288** | 0.50 | 0.20 | 0.015521 | 0.239439 |
| **K01802** | 0.55 | 0.23 | 0.015974 | 0.240392 |
| **K03702** | 0.24 | 0.10 | 0.016062 | 0.240805 |
| **K02574** | 0.75 | 0.31 | 0.016277 | 0.24176 |
| **K05919** | 0.74 | 0.30 | 0.016207 | 0.24176 |
| **K08679** | 0.90 | 0.37 | 0.016306 | 0.24176 |
| **K00941** | 0.52 | 0.22 | 0.016438 | 0.241804 |
| **K06864** | 0.76 | 0.31 | 0.016489 | 0.241804 |
| **K09747** | -0.45 | 0.19 | 0.016487 | 0.241804 |
| **K00099** | 0.28 | 0.12 | 0.016807 | 0.242525 |
| **K00147** | 0.28 | 0.12 | 0.017001 | 0.242525 |
| **K01834** | 0.26 | 0.11 | 0.016883 | 0.242525 |
| **K01897** | 0.58 | 0.24 | 0.016852 | 0.242525 |
| **K02036** | 0.25 | 0.11 | 0.017021 | 0.242525 |
| **K18830** | 0.93 | 0.39 | 0.016971 | 0.242525 |
| **K20107** | 0.79 | 0.33 | 0.016761 | 0.242525 |
| **K20108** | 0.79 | 0.33 | 0.016761 | 0.242525 |
| **K00059** | 0.36 | 0.15 | 0.017339 | 0.242892 |
| **K00878** | 0.48 | 0.20 | 0.017422 | 0.242892 |
| **K01159** | 0.25 | 0.10 | 0.01747 | 0.242892 |
| **K01575** | 1.17 | 0.49 | 0.017354 | 0.242892 |
| **K02114** | -0.39 | 0.16 | 0.017274 | 0.242892 |
| **K02529** | 0.30 | 0.12 | 0.017461 | 0.242892 |
| **K16214** | 0.55 | 0.23 | 0.017175 | 0.242892 |
| **K01493** | 0.97 | 0.40 | 0.017624 | 0.24334 |
| **K11358** | 0.81 | 0.34 | 0.017592 | 0.24334 |
| **K03439** | 0.43 | 0.18 | 0.0177 | 0.243555 |
| **K00259** | 0.98 | 0.41 | 0.018436 | 0.246746 |
| **K00324** | 0.38 | 0.16 | 0.019836 | 0.246746 |
| **K00558** | 0.91 | 0.38 | 0.018333 | 0.246746 |
| **K00656** | 0.27 | 0.11 | 0.019563 | 0.246746 |
| **K00927** | 0.17 | 0.07 | 0.019316 | 0.246746 |
| **K00943** | 0.27 | 0.12 | 0.019781 | 0.246746 |
| **K00949** | 1.07 | 0.45 | 0.018708 | 0.246746 |
| **K01186** | 0.88 | 0.37 | 0.018807 | 0.246746 |
| **K01361** | -0.64 | 0.27 | 0.018236 | 0.246746 |
| **K01495** | 0.45 | 0.19 | 0.018965 | 0.246746 |
| **K01580** | 0.81 | 0.35 | 0.020008 | 0.246746 |
| **K01659** | -1.02 | 0.43 | 0.018639 | 0.246746 |
| **K01687** | 0.21 | 0.09 | 0.019507 | 0.246746 |
| **K01697** | 0.66 | 0.28 | 0.019474 | 0.246746 |
| **K02440** | -0.61 | 0.26 | 0.018806 | 0.246746 |
| **K02956** | 0.18 | 0.07 | 0.018239 | 0.246746 |
| **K03589** | 0.31 | 0.13 | 0.019477 | 0.246746 |
| **K03596** | 0.16 | 0.07 | 0.01983 | 0.246746 |
| **K03684** | 0.81 | 0.34 | 0.018463 | 0.246746 |
| **K03761** | -0.84 | 0.35 | 0.019259 | 0.246746 |
| **K04656** | 0.90 | 0.38 | 0.02002 | 0.246746 |
| **K06915** | -0.74 | 0.32 | 0.019341 | 0.246746 |
| **K07011** | 0.60 | 0.25 | 0.018855 | 0.246746 |
| **K07250** | -0.95 | 0.40 | 0.019906 | 0.246746 |
| **K07462** | -0.75 | 0.32 | 0.018976 | 0.246746 |
| **K07738** | 0.50 | 0.21 | 0.018344 | 0.246746 |
| **K07774** | -0.55 | 0.23 | 0.018234 | 0.246746 |
| **K09762** | 0.39 | 0.16 | 0.018141 | 0.246746 |
| **K09793** | 0.92 | 0.39 | 0.018948 | 0.246746 |
| **K11900** | -0.35 | 0.15 | 0.019673 | 0.246746 |
| **K12574** | 0.57 | 0.24 | 0.019552 | 0.246746 |
| **K16147** | 0.89 | 0.38 | 0.019516 | 0.246746 |
| **K16213** | 0.90 | 0.38 | 0.019675 | 0.246746 |
| **K18139** | 0.99 | 0.42 | 0.019417 | 0.246746 |
| **K02437** | 0.62 | 0.27 | 0.020314 | 0.246863 |
| **K02904** | -0.28 | 0.12 | 0.020317 | 0.246863 |
| **K05337** | -0.95 | 0.41 | 0.020336 | 0.246863 |
| **K07223** | -0.82 | 0.35 | 0.020189 | 0.246863 |
| **K15581** | 0.41 | 0.18 | 0.020197 | 0.246863 |
| **K02871** | 0.18 | 0.08 | 0.020675 | 0.250212 |
| **K00817** | 0.28 | 0.12 | 0.021408 | 0.254729 |
| **K02835** | 0.19 | 0.08 | 0.021407 | 0.254729 |
| **K07175** | 0.90 | 0.39 | 0.021124 | 0.254729 |
| **K07498** | -1.04 | 0.45 | 0.021313 | 0.254729 |
| **K10979** | 0.59 | 0.25 | 0.021211 | 0.254729 |
| **K22501** | -0.62 | 0.27 | 0.021428 | 0.254729 |
| **K06408** | 0.48 | 0.21 | 0.021728 | 0.257533 |
| **K00931** | 0.26 | 0.11 | 0.021891 | 0.258376 |
| **K03076** | 0.56 | 0.24 | 0.021928 | 0.258376 |
| **K00851** | 0.82 | 0.36 | 0.022296 | 0.259242 |
| **K00858** | 0.27 | 0.12 | 0.022324 | 0.259242 |
| **K07154** | 0.76 | 0.33 | 0.02232 | 0.259242 |
| **K08483** | 0.50 | 0.22 | 0.022306 | 0.259242 |
| **K09810** | 0.90 | 0.39 | 0.022215 | 0.259242 |
| **K00948** | 0.23 | 0.10 | 0.022417 | 0.259574 |
| **K00065** | -0.70 | 0.31 | 0.022493 | 0.259708 |
| **K00795** | -0.64 | 0.28 | 0.022926 | 0.260214 |
| **K00868** | 0.35 | 0.15 | 0.022802 | 0.260214 |
| **K00990** | -0.85 | 0.37 | 0.022725 | 0.260214 |
| **K02007** | -0.75 | 0.33 | 0.022904 | 0.260214 |
| **K08969** | 1.15 | 0.50 | 0.022874 | 0.260214 |
| **K21636** | 0.34 | 0.15 | 0.022789 | 0.260214 |
| **K01814** | 0.20 | 0.09 | 0.023044 | 0.26082 |
| **K15868** | 0.64 | 0.28 | 0.023174 | 0.261554 |
| **K03581** | 0.62 | 0.27 | 0.023303 | 0.262275 |
| **K00611** | 0.34 | 0.15 | 0.024008 | 0.269456 |
| **K01653** | 0.20 | 0.09 | 0.024342 | 0.269715 |
| **K01704** | 0.23 | 0.10 | 0.024311 | 0.269715 |
| **K03313** | 0.41 | 0.18 | 0.024183 | 0.269715 |
| **K03786** | 0.97 | 0.43 | 0.024367 | 0.269715 |
| **K06894** | 0.73 | 0.32 | 0.024286 | 0.269715 |
| **K02436** | -0.71 | 0.31 | 0.024502 | 0.270464 |
| **K10441** | 0.92 | 0.41 | 0.024594 | 0.270731 |
| **K01304** | 0.63 | 0.28 | 0.024906 | 0.272515 |
| **K05845** | -0.61 | 0.27 | 0.025009 | 0.272515 |
| **K11535** | -0.67 | 0.30 | 0.024844 | 0.272515 |
| **K21089** | -0.79 | 0.35 | 0.025027 | 0.272515 |
| **K08094** | -0.89 | 0.39 | 0.025295 | 0.274685 |
| **K02853** | -0.76 | 0.34 | 0.025468 | 0.275081 |
| **K05364** | 1.19 | 0.53 | 0.025436 | 0.275081 |
| **K03043** | 0.21 | 0.09 | 0.025724 | 0.277102 |
| **K03746** | -0.67 | 0.30 | 0.025827 | 0.27747 |
| **K00687** | 0.46 | 0.21 | 0.025934 | 0.277879 |
| **K01156** | 1.02 | 0.46 | 0.026106 | 0.278971 |
| **K09772** | 0.39 | 0.17 | 0.026634 | 0.283859 |
| **K06948** | -0.81 | 0.36 | 0.026719 | 0.284016 |
| **K21556** | 0.91 | 0.41 | 0.027219 | 0.288561 |
| **K07122** | 0.79 | 0.35 | 0.027641 | 0.292272 |
| **K00013** | 0.20 | 0.09 | 0.027954 | 0.292662 |
| **K00997** | -0.47 | 0.21 | 0.027843 | 0.292662 |
| **K02077** | 0.75 | 0.34 | 0.027998 | 0.292662 |
| **K02864** | 0.25 | 0.11 | 0.028188 | 0.292662 |
| **K03070** | 0.23 | 0.11 | 0.028044 | 0.292662 |
| **K07220** | 0.45 | 0.20 | 0.028101 | 0.292662 |
| **K16235** | 0.94 | 0.43 | 0.028118 | 0.292662 |
| **K06941** | 0.25 | 0.11 | 0.028395 | 0.294053 |
| **K01104** | 0.45 | 0.21 | 0.028661 | 0.295161 |
| **K01462** | 0.23 | 0.10 | 0.028723 | 0.295161 |
| **K06287** | 0.21 | 0.09 | 0.028688 | 0.295161 |
| **K02108** | 0.38 | 0.17 | 0.028999 | 0.297239 |
| **K03218** | 0.29 | 0.13 | 0.029167 | 0.298197 |
| **K03580** | -0.66 | 0.30 | 0.029296 | 0.298759 |
| **K02119** | 0.70 | 0.32 | 0.029673 | 0.300281 |
| **K02600** | 0.30 | 0.14 | 0.029626 | 0.300281 |
| **K02892** | 0.15 | 0.07 | 0.029653 | 0.300281 |
| **K07054** | -0.71 | 0.32 | 0.029744 | 0.300281 |
| **K01892** | 0.23 | 0.10 | 0.029851 | 0.300605 |
| **K00383** | -0.53 | 0.24 | 0.030278 | 0.303756 |
| **K12952** | 0.85 | 0.39 | 0.030315 | 0.303756 |
| **K03658** | 0.80 | 0.37 | 0.030526 | 0.305109 |
| **K11991** | 0.84 | 0.39 | 0.03065 | 0.305583 |
| **K01940** | 0.25 | 0.12 | 0.030737 | 0.305701 |
| **K06167** | 0.89 | 0.41 | 0.030997 | 0.307517 |
| **K02003** | 0.23 | 0.11 | 0.031152 | 0.308297 |
| **K00254** | -0.69 | 0.32 | 0.031391 | 0.30913 |
| **K00800** | 0.26 | 0.12 | 0.032082 | 0.30913 |
| **K02371** | 0.69 | 0.32 | 0.031861 | 0.30913 |
| **K02895** | 0.20 | 0.09 | 0.031791 | 0.30913 |
| **K03531** | 0.50 | 0.23 | 0.031745 | 0.30913 |
| **K04338** | -0.40 | 0.19 | 0.03162 | 0.30913 |
| **K07741** | -1.10 | 0.51 | 0.031857 | 0.30913 |
| **K14982** | -0.69 | 0.32 | 0.032067 | 0.30913 |
| **K17828** | 0.42 | 0.20 | 0.031501 | 0.30913 |
| **K18815** | -0.58 | 0.27 | 0.032075 | 0.30913 |
| **K22486** | 0.61 | 0.28 | 0.031645 | 0.30913 |
| **K02109** | 0.22 | 0.10 | 0.032405 | 0.310883 |
| **K06166** | 0.57 | 0.26 | 0.032419 | 0.310883 |
| **K00549** | 0.30 | 0.14 | 0.032525 | 0.311155 |
| **K00128** | 0.99 | 0.46 | 0.032637 | 0.31149 |
| **K01916** | -0.51 | 0.24 | 0.032778 | 0.312092 |
| **K00123** | 0.63 | 0.29 | 0.033103 | 0.314437 |
| **K00763** | 0.65 | 0.30 | 0.033625 | 0.316763 |
| **K02356** | 0.22 | 0.10 | 0.033899 | 0.316763 |
| **K02888** | 0.21 | 0.10 | 0.033637 | 0.316763 |
| **K03438** | 0.22 | 0.10 | 0.03371 | 0.316763 |
| **K07089** | 0.56 | 0.26 | 0.033798 | 0.316763 |
| **K11533** | -0.48 | 0.22 | 0.033886 | 0.316763 |
| **K14053** | 0.82 | 0.39 | 0.033846 | 0.316763 |
| **K07979** | 0.89 | 0.42 | 0.03401 | 0.317061 |
| **K03550** | 0.28 | 0.13 | 0.034418 | 0.320118 |
| **K01847** | -0.81 | 0.38 | 0.03458 | 0.320148 |
| **K03536** | 0.22 | 0.10 | 0.034515 | 0.320148 |
| **K01785** | 0.38 | 0.18 | 0.034797 | 0.321409 |
| **K04064** | -0.74 | 0.35 | 0.035301 | 0.324957 |
| **K20038** | 0.39 | 0.19 | 0.035343 | 0.324957 |
| **K01414** | -0.57 | 0.27 | 0.035606 | 0.325891 |
| **K02761** | -0.60 | 0.28 | 0.03556 | 0.325891 |
| **K01246** | 0.52 | 0.25 | 0.036178 | 0.329624 |
| **K05835** | -0.72 | 0.34 | 0.036169 | 0.329624 |
| **K01625** | -0.49 | 0.23 | 0.036732 | 0.333178 |
| **K12373** | 1.23 | 0.59 | 0.036734 | 0.333178 |
| **K00054** | -0.62 | 0.29 | 0.036839 | 0.333281 |
| **K00266** | 0.63 | 0.30 | 0.03716 | 0.333281 |
| **K01890** | 0.29 | 0.14 | 0.037071 | 0.333281 |
| **K07724** | 0.64 | 0.31 | 0.037015 | 0.333281 |
| **K13252** | 0.93 | 0.45 | 0.037131 | 0.333281 |
| **K01883** | 0.22 | 0.10 | 0.037426 | 0.334914 |
| **K04753** | -0.54 | 0.26 | 0.037609 | 0.335807 |
| **K07668** | -0.78 | 0.37 | 0.037751 | 0.336331 |
| **K01250** | 0.91 | 0.43 | 0.038013 | 0.336421 |
| **K07454** | 1.00 | 0.48 | 0.037979 | 0.336421 |
| **K16012** | -0.67 | 0.32 | 0.037962 | 0.336421 |
| **K07243** | 1.01 | 0.48 | 0.038273 | 0.337984 |
| **K00060** | -0.54 | 0.26 | 0.038718 | 0.339589 |
| **K03558** | 0.66 | 0.32 | 0.038793 | 0.339589 |
| **K03631** | 0.75 | 0.36 | 0.038609 | 0.339589 |
| **K11618** | -0.79 | 0.38 | 0.038671 | 0.339589 |
| **K05352** | -0.50 | 0.24 | 0.039211 | 0.342495 |
| **K03551** | 0.18 | 0.09 | 0.039684 | 0.344854 |
| **K09456** | 0.75 | 0.36 | 0.039738 | 0.344854 |
| **K13918** | -0.61 | 0.29 | 0.039715 | 0.344854 |
| **K00615** | 0.21 | 0.10 | 0.040174 | 0.345132 |
| **K01727** | -0.57 | 0.28 | 0.040285 | 0.345132 |
| **K03294** | 0.43 | 0.21 | 0.040179 | 0.345132 |
| **K03524** | 0.31 | 0.15 | 0.04025 | 0.345132 |
| **K03704** | 0.16 | 0.08 | 0.039942 | 0.345132 |
| **K14088** | 0.52 | 0.25 | 0.040065 | 0.345132 |
| **K02428** | 0.27 | 0.13 | 0.040588 | 0.34698 |
| **K00134** | 0.32 | 0.15 | 0.040922 | 0.347099 |
| **K01809** | -0.63 | 0.31 | 0.040842 | 0.347099 |
| **K06987** | 0.60 | 0.29 | 0.04077 | 0.347099 |
| **K11754** | 0.53 | 0.26 | 0.040947 | 0.347099 |
| **K03086** | 0.35 | 0.17 | 0.041077 | 0.34747 |
| **K01620** | 0.63 | 0.31 | 0.041538 | 0.349894 |
| **K07033** | 0.53 | 0.26 | 0.041467 | 0.349894 |
| **K05995** | 0.76 | 0.37 | 0.041826 | 0.351228 |
| **K15587** | 0.84 | 0.41 | 0.041871 | 0.351228 |
| **K00394** | 0.46 | 0.22 | 0.042001 | 0.351582 |
| **K00820** | 0.24 | 0.12 | 0.042283 | 0.353205 |
| **K02994** | 0.19 | 0.09 | 0.042714 | 0.354716 |
| **K03040** | 0.18 | 0.09 | 0.042695 | 0.354716 |
| **K06215** | 0.37 | 0.18 | 0.042728 | 0.354716 |
| **K00841** | -0.75 | 0.37 | 0.043352 | 0.354842 |
| **K01077** | 0.61 | 0.30 | 0.04339 | 0.354842 |
| **K01924** | 0.23 | 0.11 | 0.043316 | 0.354842 |
| **K03169** | 0.74 | 0.36 | 0.04345 | 0.354842 |
| **K03762** | 0.65 | 0.32 | 0.04308 | 0.354842 |
| **K04042** | 0.23 | 0.11 | 0.043431 | 0.354842 |
| **K06400** | -0.32 | 0.16 | 0.043111 | 0.354842 |
| **K09181** | -0.60 | 0.29 | 0.043285 | 0.354842 |
| **K11740** | 0.73 | 0.36 | 0.043829 | 0.357207 |
| **K16511** | -0.56 | 0.28 | 0.044008 | 0.357946 |
| **K02117** | 0.64 | 0.32 | 0.044152 | 0.35839 |
| **K11068** | 0.49 | 0.24 | 0.044491 | 0.36041 |
| **K01496** | 0.27 | 0.13 | 0.044867 | 0.361651 |
| **K14260** | 0.66 | 0.33 | 0.044914 | 0.361651 |
| **K22226** | 0.44 | 0.22 | 0.044829 | 0.361651 |
| **K00527** | -0.61 | 0.30 | 0.045046 | 0.361992 |
| **K09765** | 0.94 | 0.46 | 0.045274 | 0.363092 |
| **K01703** | 0.18 | 0.09 | 0.045578 | 0.363811 |
| **K01810** | 0.19 | 0.09 | 0.045502 | 0.363811 |
| **K02935** | 0.20 | 0.10 | 0.045635 | 0.363811 |
| **K09971** | -0.57 | 0.28 | 0.045926 | 0.36541 |
| **K00965** | 0.47 | 0.23 | 0.04622 | 0.365482 |
| **K03564** | 0.57 | 0.29 | 0.04639 | 0.365482 |
| **K06946** | -1.01 | 0.50 | 0.04636 | 0.365482 |
| **K07386** | 0.86 | 0.43 | 0.046104 | 0.365482 |
| **K07481** | -1.04 | 0.52 | 0.046162 | 0.365482 |
| **K01523** | 0.27 | 0.13 | 0.046625 | 0.366613 |
| **K01644** | -0.47 | 0.24 | 0.047131 | 0.368567 |
| **K12369** | -0.60 | 0.30 | 0.047149 | 0.368567 |
| **K17331** | 1.13 | 0.57 | 0.047142 | 0.368567 |
| **K00198** | -0.62 | 0.31 | 0.047675 | 0.37052 |
| **K04043** | 0.14 | 0.07 | 0.047531 | 0.37052 |
| **K13963** | -0.74 | 0.37 | 0.047603 | 0.37052 |
| **K05595** | 0.83 | 0.42 | 0.048127 | 0.371873 |
| **K08222** | 0.63 | 0.32 | 0.048061 | 0.371873 |
| **K09023** | 0.75 | 0.38 | 0.048098 | 0.371873 |
| **K02933** | 0.17 | 0.09 | 0.048297 | 0.372046 |
| **K07082** | 0.26 | 0.13 | 0.048335 | 0.372046 |
| **K01866** | 0.18 | 0.09 | 0.048461 | 0.372304 |
| **K01876** | 0.23 | 0.11 | 0.049302 | 0.378047 |
| **K07307** | 0.58 | 0.30 | 0.049765 | 0.380871 |
| **K00639** | -0.65 | 0.33 | 0.050728 | 0.382467 |
| **K00832** | -0.69 | 0.35 | 0.050192 | 0.382467 |
| **K01515** | -0.39 | 0.20 | 0.050415 | 0.382467 |
| **K01649** | 0.33 | 0.17 | 0.050735 | 0.382467 |
| **K02887** | 0.15 | 0.07 | 0.050351 | 0.382467 |
| **K03168** | 0.52 | 0.26 | 0.050642 | 0.382467 |
| **K09773** | -0.60 | 0.31 | 0.050541 | 0.382467 |
| **K18479** | 0.76 | 0.39 | 0.050482 | 0.382467 |
| **K00925** | -0.32 | 0.16 | 0.05093 | 0.383188 |
|  |  |  |  |  |
|  |  |  |  |  |
|  |  |  |  |  |
|  |  |  |  |  |
|  |  |  |  |  |
|  |  |  |  |  |
|  |  |  |  |  |
|  |  |  |  |  |
|  |  |  |  |  |
|  |  |  |  |  |
|  |  |  |  |  |

Supplementary Table S4. Gene family differences associated with siblings at 12 months.

| **KEGG** | **Coefficent** | **SD** | **P raw** | **P FDR adjusted** |
| --- | --- | --- | --- | --- |
| K04032 | -1.43 | 0.26 | 1.08E-07 | 0.000131 |
| K05351 | 3.08 | 0.57 | 1.3E-07 | 0.000131 |
| K06117 | 3.05 | 0.55 | 7.35E-08 | 0.000131 |
| K16211 | 2.25 | 0.40 | 4.86E-08 | 0.000131 |
| K07405 | 1.47 | 0.29 | 7.02E-07 | 0.000568 |
| K03503 | 1.81 | 0.36 | 1.16E-06 | 0.000673 |
| K11534 | -1.22 | 0.24 | 1.05E-06 | 0.000673 |
| K10914 | -1.19 | 0.24 | 1.51E-06 | 0.000763 |
| K02414 | -1.29 | 0.26 | 1.88E-06 | 0.000789 |
| K04073 | -1.77 | 0.36 | 2.07E-06 | 0.000789 |
| K18118 | -1.51 | 0.31 | 2.15E-06 | 0.000789 |
| K01489 | -0.81 | 0.17 | 2.65E-06 | 0.000891 |
| K06442 | 1.63 | 0.34 | 2.91E-06 | 0.000891 |
| K09973 | -0.95 | 0.20 | 3.08E-06 | 0.000891 |
| K02074 | 2.54 | 0.53 | 3.43E-06 | 0.000927 |
| K16153 | 1.46 | 0.31 | 3.89E-06 | 0.000984 |
| K11784 | 1.53 | 0.33 | 6.92E-06 | 0.001648 |
| K01070 | -1.51 | 0.33 | 7.92E-06 | 0.001687 |
| K04565 | -1.26 | 0.28 | 7.7E-06 | 0.001687 |
| K01026 | 1.75 | 0.39 | 9.45E-06 | 0.001811 |
| K01849 | -1.22 | 0.27 | 1.05E-05 | 0.001811 |
| K02812 | 1.45 | 0.32 | 1.01E-05 | 0.001811 |
| K02813 | 1.71 | 0.38 | 1.07E-05 | 0.001811 |
| K03666 | -1.08 | 0.24 | 1.03E-05 | 0.001811 |
| K10003 | -1.58 | 0.36 | 1.24E-05 | 0.001999 |
| K03449 | -1.77 | 0.40 | 1.57E-05 | 0.002284 |
| K03647 | 1.48 | 0.34 | 1.52E-05 | 0.002284 |
| K04028 | -1.00 | 0.23 | 1.6E-05 | 0.002284 |
| K08156 | 2.05 | 0.47 | 1.64E-05 | 0.002284 |
| K07689 | -1.46 | 0.33 | 1.71E-05 | 0.002304 |
| K01644 | -1.43 | 0.33 | 1.88E-05 | 0.002386 |
| K03651 | -1.37 | 0.31 | 1.95E-05 | 0.002386 |
| K05685 | -1.30 | 0.30 | 1.9E-05 | 0.002386 |
| K12265 | -1.16 | 0.27 | 2.05E-05 | 0.002446 |
| K03338 | 1.33 | 0.31 | 2.25E-05 | 0.00245 |
| K07652 | -1.03 | 0.24 | 2.22E-05 | 0.00245 |
| K13637 | -1.39 | 0.32 | 2.3E-05 | 0.00245 |
| K18581 | -1.61 | 0.37 | 2.14E-05 | 0.00245 |
| K02317 | -1.45 | 0.34 | 2.38E-05 | 0.002468 |
| K00394 | 1.49 | 0.35 | 3E-05 | 0.002491 |
| K01023 | -0.94 | 0.22 | 2.64E-05 | 0.002491 |
| K01584 | -1.14 | 0.27 | 3E-05 | 0.002491 |
| K01843 | 1.08 | 0.25 | 2.56E-05 | 0.002491 |
| K03502 | 0.80 | 0.19 | 2.59E-05 | 0.002491 |
| K03735 | -1.09 | 0.26 | 3.04E-05 | 0.002491 |
| K03812 | -1.12 | 0.26 | 2.96E-05 | 0.002491 |
| K05245 | -1.48 | 0.35 | 2.74E-05 | 0.002491 |
| K06191 | 1.65 | 0.39 | 2.8E-05 | 0.002491 |
| K07406 | -1.25 | 0.29 | 2.74E-05 | 0.002491 |
| K12370 | -1.48 | 0.35 | 3.08E-05 | 0.002491 |
| K02048 | -1.57 | 0.37 | 3.19E-05 | 0.002513 |
| K15827 | -1.64 | 0.39 | 3.23E-05 | 0.002513 |
| K03413 | -0.87 | 0.21 | 3.4E-05 | 0.002597 |
| K16248 | 1.47 | 0.35 | 3.47E-05 | 0.002601 |
| K03306 | -1.00 | 0.24 | 3.63E-05 | 0.002665 |
| K04027 | -1.19 | 0.28 | 3.75E-05 | 0.002665 |
| K22906 | -1.11 | 0.27 | 3.73E-05 | 0.002665 |
| K00374 | -1.51 | 0.36 | 3.91E-05 | 0.002685 |
| K03389 | 1.34 | 0.32 | 3.91E-05 | 0.002685 |
| K04023 | -1.10 | 0.26 | 4.16E-05 | 0.002807 |
| K03660 | 1.66 | 0.40 | 4.26E-05 | 0.002828 |
| K02339 | -1.33 | 0.32 | 4.76E-05 | 0.003059 |
| K08222 | 1.69 | 0.41 | 4.72E-05 | 0.003059 |
| K01299 | 1.71 | 0.41 | 4.96E-05 | 0.003137 |
| K01207 | -1.23 | 0.30 | 5.16E-05 | 0.003213 |
| K05845 | -0.79 | 0.19 | 5.3E-05 | 0.003225 |
| K09124 | 1.76 | 0.43 | 5.45E-05 | 0.003225 |
| K13963 | -1.02 | 0.25 | 5.39E-05 | 0.003225 |
| K15832 | -1.58 | 0.39 | 5.5E-05 | 0.003225 |
| K10804 | -1.19 | 0.29 | 5.77E-05 | 0.003244 |
| K15634 | -1.58 | 0.39 | 5.73E-05 | 0.003244 |
| K21512 | -1.05 | 0.25 | 5.66E-05 | 0.003244 |
| K12113 | -1.23 | 0.30 | 5.98E-05 | 0.003317 |
| K09009 | 2.04 | 0.50 | 6.33E-05 | 0.003463 |
| K02467 | -1.18 | 0.29 | 6.52E-05 | 0.003501 |
| K21090 | -1.23 | 0.30 | 6.57E-05 | 0.003501 |
| K01848 | -1.00 | 0.25 | 6.76E-05 | 0.003549 |
| K06518 | -1.17 | 0.29 | 6.96E-05 | 0.003549 |
| K11719 | -1.28 | 0.32 | 7.02E-05 | 0.003549 |
| K19709 | -1.28 | 0.32 | 6.99E-05 | 0.003549 |
| K01449 | -0.89 | 0.22 | 7.47E-05 | 0.003642 |
| K01638 | -1.50 | 0.37 | 7.46E-05 | 0.003642 |
| K08994 | -1.05 | 0.26 | 7.35E-05 | 0.003642 |
| K00395 | 1.39 | 0.34 | 7.67E-05 | 0.003694 |
| K05895 | 1.57 | 0.39 | 7.85E-05 | 0.003736 |
| K02405 | -0.91 | 0.23 | 8.19E-05 | 0.003743 |
| K03535 | -1.18 | 0.29 | 8.06E-05 | 0.003743 |
| K03758 | -0.96 | 0.24 | 8.23E-05 | 0.003743 |
| K07771 | -1.28 | 0.32 | 8.06E-05 | 0.003743 |
| K00334 | -1.06 | 0.26 | 8.53E-05 | 0.003836 |
| K00936 | -0.90 | 0.23 | 8.85E-05 | 0.003937 |
| K08351 | -1.30 | 0.33 | 9.12E-05 | 0.003968 |
| K18234 | 1.13 | 0.28 | 9.03E-05 | 0.003968 |
| K02768 | 1.16 | 0.29 | 9.42E-05 | 0.004054 |
| K05808 | -0.79 | 0.20 | 9.52E-05 | 0.004055 |
| K03748 | -1.25 | 0.32 | 9.73E-05 | 0.00406 |
| K05967 | 1.44 | 0.36 | 9.7E-05 | 0.00406 |
| K03078 | -1.44 | 0.36 | 0.0001 | 0.004138 |
| K09893 | -1.33 | 0.34 | 0.000109 | 0.004454 |
| K10823 | -0.86 | 0.22 | 0.000115 | 0.004641 |
| K02389 | -0.84 | 0.22 | 0.000118 | 0.004672 |
| K07773 | -1.46 | 0.37 | 0.000118 | 0.004672 |
| K21993 | 1.13 | 0.29 | 0.000119 | 0.004672 |
| K00045 | 1.54 | 0.39 | 0.000121 | 0.004676 |
| K01060 | -0.96 | 0.25 | 0.000127 | 0.004676 |
| K01706 | -1.41 | 0.36 | 0.000121 | 0.004676 |
| K01779 | 1.99 | 0.51 | 0.000128 | 0.004676 |
| K02495 | -1.26 | 0.32 | 0.000129 | 0.004676 |
| K03838 | -1.31 | 0.34 | 0.000123 | 0.004676 |
| K04333 | -1.37 | 0.35 | 0.000129 | 0.004676 |
| K21885 | 1.48 | 0.38 | 0.000124 | 0.004676 |
| K22757 | 1.01 | 0.26 | 0.000128 | 0.004676 |
| K10000 | -1.36 | 0.35 | 0.000131 | 0.004696 |
| K01205 | 1.79 | 0.46 | 0.000133 | 0.004704 |
| K03855 | -1.25 | 0.32 | 0.000135 | 0.004704 |
| K18765 | -1.18 | 0.30 | 0.000135 | 0.004704 |
| K09899 | -1.16 | 0.30 | 0.000136 | 0.004705 |
| K03319 | -1.62 | 0.42 | 0.000141 | 0.00476 |
| K03435 | -1.43 | 0.37 | 0.000139 | 0.00476 |
| K13940 | 1.42 | 0.37 | 0.00014 | 0.00476 |
| K02478 | -1.18 | 0.31 | 0.000143 | 0.004769 |
| K07272 | 1.70 | 0.44 | 0.000145 | 0.004769 |
| K09020 | -1.15 | 0.30 | 0.000144 | 0.004769 |
| K04651 | -1.07 | 0.28 | 0.000151 | 0.004912 |
| K01227 | 1.32 | 0.34 | 0.00016 | 0.004947 |
| K01277 | 1.50 | 0.39 | 0.000155 | 0.004947 |
| K02598 | -1.21 | 0.32 | 0.000159 | 0.004947 |
| K03598 | -1.27 | 0.33 | 0.000158 | 0.004947 |
| K04691 | -1.35 | 0.35 | 0.000153 | 0.004947 |
| K13920 | -0.93 | 0.24 | 0.000159 | 0.004947 |
| K16203 | -0.97 | 0.25 | 0.000159 | 0.004947 |
| K10036 | -1.35 | 0.35 | 0.000164 | 0.005027 |
| K09904 | -1.28 | 0.33 | 0.000166 | 0.005062 |
| K01582 | -1.29 | 0.34 | 0.000168 | 0.005076 |
| K06193 | -1.45 | 0.38 | 0.00017 | 0.005087 |
| K05846 | -0.79 | 0.21 | 0.000175 | 0.005213 |
| K00171 | 1.33 | 0.35 | 0.000188 | 0.005388 |
| K02170 | -1.32 | 0.35 | 0.000191 | 0.005388 |
| K02484 | -1.19 | 0.31 | 0.000184 | 0.005388 |
| K02818 | -1.46 | 0.39 | 0.000187 | 0.005388 |
| K02819 | -1.46 | 0.38 | 0.000188 | 0.005388 |
| K04024 | -1.02 | 0.27 | 0.00019 | 0.005388 |
| K04074 | 1.43 | 0.38 | 0.000193 | 0.005388 |
| K07645 | -1.10 | 0.29 | 0.000192 | 0.005388 |
| K11708 | -1.46 | 0.38 | 0.000187 | 0.005388 |
| K01239 | 2.11 | 0.56 | 0.000195 | 0.005403 |
| K02526 | -1.19 | 0.31 | 0.000198 | 0.005409 |
| K04094 | -0.81 | 0.21 | 0.000197 | 0.005409 |
| K01825 | -1.27 | 0.34 | 0.000203 | 0.005475 |
| K04026 | -0.80 | 0.21 | 0.000203 | 0.005475 |
| K16322 | -1.31 | 0.35 | 0.00021 | 0.00564 |
| K06186 | -1.41 | 0.38 | 0.000216 | 0.005678 |
| K07749 | -1.16 | 0.31 | 0.000215 | 0.005678 |
| K08160 | -1.27 | 0.34 | 0.000214 | 0.005678 |
| K18778 | -1.25 | 0.33 | 0.000218 | 0.005687 |
| K13480 | -1.23 | 0.33 | 0.000222 | 0.005753 |
| K05501 | -1.43 | 0.38 | 0.000225 | 0.005799 |
| K01081 | 1.65 | 0.44 | 0.000228 | 0.005831 |
| K00042 | -1.33 | 0.36 | 0.000238 | 0.005877 |
| K01071 | 1.58 | 0.42 | 0.000235 | 0.005877 |
| K03404 | -0.91 | 0.24 | 0.000232 | 0.005877 |
| K03605 | -1.35 | 0.36 | 0.00024 | 0.005877 |
| K03828 | -1.22 | 0.33 | 0.000239 | 0.005877 |
| K04744 | -1.18 | 0.32 | 0.000234 | 0.005877 |
| K21089 | -0.93 | 0.25 | 0.000238 | 0.005877 |
| K00840 | -1.20 | 0.32 | 0.000247 | 0.005891 |
| K00932 | -1.21 | 0.33 | 0.000244 | 0.005891 |
| K00990 | -1.43 | 0.38 | 0.000246 | 0.005891 |
| K03597 | -1.28 | 0.34 | 0.000247 | 0.005891 |
| K05787 | -1.56 | 0.42 | 0.000246 | 0.005891 |
| K20885 | 1.47 | 0.40 | 0.000252 | 0.005972 |
| K20345 | 1.53 | 0.41 | 0.000256 | 0.006032 |
| K05919 | 1.19 | 0.32 | 0.000265 | 0.006125 |
| K06141 | -1.21 | 0.33 | 0.000262 | 0.006125 |
| K18139 | 1.76 | 0.47 | 0.000265 | 0.006125 |
| K04835 | -1.10 | 0.30 | 0.000268 | 0.006157 |
| K00355 | 1.49 | 0.40 | 0.000274 | 0.006166 |
| K06997 | 0.52 | 0.14 | 0.000274 | 0.006166 |
| K08680 | -1.62 | 0.44 | 0.000274 | 0.006166 |
| K11733 | -1.54 | 0.42 | 0.000271 | 0.006166 |
| K01611 | -1.53 | 0.41 | 0.000282 | 0.006297 |
| K01104 | 0.55 | 0.15 | 0.000287 | 0.006357 |
| K08990 | -1.04 | 0.28 | 0.000287 | 0.006357 |
| K04765 | -1.44 | 0.39 | 0.000294 | 0.00644 |
| K06378 | -0.82 | 0.22 | 0.000294 | 0.00644 |
| K23004 | 1.62 | 0.44 | 0.000297 | 0.006464 |
| K02043 | -1.00 | 0.27 | 0.000302 | 0.006494 |
| K03286 | -1.36 | 0.37 | 0.000301 | 0.006494 |
| K02049 | -0.69 | 0.19 | 0.000312 | 0.006575 |
| K05916 | -1.01 | 0.28 | 0.000309 | 0.006575 |
| K06891 | -1.42 | 0.39 | 0.000312 | 0.006575 |
| K07287 | -1.26 | 0.34 | 0.000313 | 0.006575 |
| K13892 | -1.32 | 0.36 | 0.000314 | 0.006575 |
| K04568 | -1.32 | 0.36 | 0.000319 | 0.006662 |
| K05275 | -1.20 | 0.33 | 0.000328 | 0.006775 |
| K07283 | -0.95 | 0.26 | 0.000328 | 0.006775 |
| K01664 | -1.25 | 0.34 | 0.000333 | 0.006835 |
| K01575 | 1.75 | 0.48 | 0.000343 | 0.006943 |
| K02380 | -0.94 | 0.26 | 0.000345 | 0.006943 |
| K04030 | -0.82 | 0.23 | 0.000342 | 0.006943 |
| K06373 | -0.85 | 0.23 | 0.000345 | 0.006943 |
| K05851 | -1.38 | 0.38 | 0.000348 | 0.006981 |
| K11615 | -0.87 | 0.24 | 0.000353 | 0.007037 |
| K09916 | -1.19 | 0.33 | 0.000358 | 0.007102 |
| K05775 | -1.37 | 0.38 | 0.000361 | 0.007129 |
| K07811 | -1.03 | 0.28 | 0.000371 | 0.007285 |
| K03796 | -1.19 | 0.33 | 0.000376 | 0.007356 |
| K11107 | -1.43 | 0.40 | 0.000378 | 0.007359 |
| K02302 | -1.36 | 0.38 | 0.000381 | 0.007365 |
| K03468 | -1.19 | 0.33 | 0.000386 | 0.007365 |
| K20881 | -1.06 | 0.29 | 0.000385 | 0.007365 |
| K22293 | 1.66 | 0.46 | 0.000384 | 0.007365 |
| K02746 | 1.81 | 0.50 | 0.000389 | 0.0074 |
| K11746 | -1.14 | 0.32 | 0.000392 | 0.007422 |
| K07006 | 1.60 | 0.45 | 0.000394 | 0.007424 |
| K00557 | -1.15 | 0.32 | 0.000401 | 0.007482 |
| K03669 | -1.19 | 0.33 | 0.000401 | 0.007482 |
| K06189 | -1.24 | 0.35 | 0.000406 | 0.007538 |
| K21511 | -0.83 | 0.23 | 0.00041 | 0.00757 |
| K02507 | -1.04 | 0.29 | 0.000414 | 0.007584 |
| K19775 | -1.23 | 0.34 | 0.000414 | 0.007584 |
| K00381 | -1.29 | 0.36 | 0.000425 | 0.007634 |
| K00799 | -1.31 | 0.37 | 0.000426 | 0.007634 |
| K02120 | -0.69 | 0.19 | 0.000423 | 0.007634 |
| K11066 | -1.07 | 0.30 | 0.000423 | 0.007634 |
| K18141 | -1.13 | 0.31 | 0.000425 | 0.007634 |
| K11732 | -1.16 | 0.33 | 0.000429 | 0.007656 |
| K00163 | -1.20 | 0.34 | 0.00044 | 0.00781 |
| K04764 | -1.27 | 0.36 | 0.000447 | 0.00786 |
| K07711 | -1.28 | 0.36 | 0.000446 | 0.00786 |
| K02775 | 1.33 | 0.37 | 0.00045 | 0.007891 |
| K07471 | -1.01 | 0.28 | 0.000455 | 0.007898 |
| K09477 | -1.04 | 0.29 | 0.000455 | 0.007898 |
| K01414 | -1.07 | 0.30 | 0.000461 | 0.007908 |
| K03326 | -1.53 | 0.43 | 0.000461 | 0.007908 |
| K15584 | 1.53 | 0.43 | 0.000461 | 0.007908 |
| K01753 | -1.21 | 0.34 | 0.000471 | 0.008047 |
| K06219 | -1.18 | 0.33 | 0.000473 | 0.008051 |
| K01661 | -0.88 | 0.25 | 0.00048 | 0.008102 |
| K02562 | -1.35 | 0.38 | 0.00048 | 0.008102 |
| K02417 | -1.36 | 0.38 | 0.00049 | 0.00811 |
| K07642 | -1.08 | 0.30 | 0.00049 | 0.00811 |
| K09159 | -1.47 | 0.41 | 0.000487 | 0.00811 |
| K10037 | -1.42 | 0.40 | 0.000486 | 0.00811 |
| K21967 | -0.93 | 0.26 | 0.000491 | 0.00811 |
| K06182 | -1.39 | 0.39 | 0.000499 | 0.008115 |
| K10541 | -0.85 | 0.24 | 0.000495 | 0.008115 |
| K14744 | -1.34 | 0.38 | 0.000499 | 0.008115 |
| K22607 | -1.23 | 0.35 | 0.000499 | 0.008115 |
| K00568 | -1.18 | 0.34 | 0.000503 | 0.008134 |
| K07701 | -1.02 | 0.29 | 0.000506 | 0.008134 |
| K12264 | -1.13 | 0.32 | 0.000505 | 0.008134 |
| K03783 | -0.85 | 0.24 | 0.000521 | 0.008223 |
| K04082 | -1.11 | 0.32 | 0.000516 | 0.008223 |
| K09516 | 1.06 | 0.30 | 0.000517 | 0.008223 |
| K13771 | -1.23 | 0.35 | 0.000527 | 0.008223 |
| K17074 | -0.80 | 0.23 | 0.000532 | 0.008223 |
| K17713 | -1.25 | 0.36 | 0.000532 | 0.008223 |
| K18326 | -1.27 | 0.36 | 0.000523 | 0.008223 |
| K18988 | -1.10 | 0.31 | 0.000529 | 0.008223 |
| K19591 | -1.14 | 0.33 | 0.000527 | 0.008223 |
| K20116 | 1.18 | 0.34 | 0.000531 | 0.008223 |
| K12142 | -0.82 | 0.23 | 0.000542 | 0.008345 |
| K00974 | -0.59 | 0.17 | 0.000545 | 0.008352 |
| K12528 | -0.88 | 0.25 | 0.000547 | 0.008352 |
| K16789 | -0.86 | 0.25 | 0.000549 | 0.008352 |
| K12529 | -1.07 | 0.31 | 0.000553 | 0.008384 |
| K08137 | -1.20 | 0.34 | 0.000559 | 0.008448 |
| K14153 | -0.57 | 0.16 | 0.000566 | 0.008514 |
| K05966 | -1.27 | 0.36 | 0.00058 | 0.008635 |
| K17076 | -0.86 | 0.25 | 0.000578 | 0.008635 |
| K21472 | -1.13 | 0.33 | 0.000579 | 0.008635 |
| K02392 | -0.80 | 0.23 | 0.000596 | 0.008765 |
| K02420 | -0.70 | 0.20 | 0.000593 | 0.008765 |
| K12943 | -1.12 | 0.32 | 0.0006 | 0.008765 |
| K16651 | -1.13 | 0.32 | 0.000595 | 0.008765 |
| K19784 | -1.12 | 0.32 | 0.0006 | 0.008765 |
| K06155 | -1.08 | 0.31 | 0.000607 | 0.008836 |
| K03599 | -1.28 | 0.37 | 0.000612 | 0.008879 |
| K03532 | -0.97 | 0.28 | 0.000616 | 0.008899 |
| K09969 | -1.12 | 0.32 | 0.000622 | 0.008951 |
| K02413 | -0.81 | 0.24 | 0.000636 | 0.009051 |
| K03776 | -0.95 | 0.27 | 0.000639 | 0.009051 |
| K07260 | 1.21 | 0.35 | 0.00064 | 0.009051 |
| K13570 | 1.22 | 0.35 | 0.000636 | 0.009051 |
| K14261 | -1.01 | 0.29 | 0.000636 | 0.009051 |
| K07274 | -1.07 | 0.31 | 0.000647 | 0.0091 |
| K15723 | -1.18 | 0.34 | 0.000648 | 0.0091 |
| K00639 | -1.34 | 0.39 | 0.00066 | 0.009245 |
| K06202 | -1.17 | 0.34 | 0.000667 | 0.009303 |
| K10111 | -1.19 | 0.34 | 0.000669 | 0.009303 |
| K03688 | 0.71 | 0.21 | 0.000672 | 0.009313 |
| K01920 | -1.26 | 0.37 | 0.00068 | 0.00939 |
| K02399 | -0.88 | 0.26 | 0.000693 | 0.009523 |
| K19304 | -1.13 | 0.33 | 0.000694 | 0.009523 |
| K08317 | -1.04 | 0.30 | 0.000697 | 0.009524 |
| K02393 | -1.24 | 0.36 | 0.000704 | 0.00954 |
| K02396 | -0.66 | 0.19 | 0.000706 | 0.00954 |
| K03558 | 1.06 | 0.31 | 0.000708 | 0.00954 |
| K07678 | -0.86 | 0.25 | 0.00071 | 0.00954 |
| K14654 | -1.04 | 0.30 | 0.000703 | 0.00954 |
| K06163 | -1.09 | 0.32 | 0.000718 | 0.009567 |
| K07224 | -1.06 | 0.31 | 0.000718 | 0.009567 |
| K12289 | -0.74 | 0.22 | 0.000719 | 0.009567 |
| K02024 | -1.34 | 0.39 | 0.000725 | 0.009572 |
| K05798 | -1.33 | 0.39 | 0.000729 | 0.009572 |
| K06073 | -1.29 | 0.38 | 0.000724 | 0.009572 |
| K13894 | -1.25 | 0.37 | 0.00073 | 0.009572 |
| K19227 | -1.10 | 0.32 | 0.000731 | 0.009572 |
| K00963 | -1.03 | 0.30 | 0.000735 | 0.009595 |
| K02446 | -1.16 | 0.34 | 0.000749 | 0.009612 |
| K03840 | -1.21 | 0.35 | 0.000749 | 0.009612 |
| K07140 | -0.91 | 0.27 | 0.000749 | 0.009612 |
| K07232 | -1.02 | 0.30 | 0.000753 | 0.009612 |
| K08989 | -1.61 | 0.47 | 0.000742 | 0.009612 |
| K12963 | -0.97 | 0.28 | 0.000752 | 0.009612 |
| K18697 | -0.98 | 0.29 | 0.000752 | 0.009612 |
| K13922 | -0.82 | 0.24 | 0.000761 | 0.009679 |
| K07235 | -1.28 | 0.38 | 0.000789 | 0.010011 |
| K00048 | -1.31 | 0.39 | 0.000796 | 0.010035 |
| K05805 | -1.21 | 0.36 | 0.000795 | 0.010035 |
| K01252 | -1.07 | 0.32 | 0.000822 | 0.010048 |
| K02106 | -0.80 | 0.24 | 0.000818 | 0.010048 |
| K03672 | -1.10 | 0.32 | 0.000823 | 0.010048 |
| K05305 | 1.03 | 0.30 | 0.000805 | 0.010048 |
| K05802 | -0.84 | 0.25 | 0.000809 | 0.010048 |
| K05804 | -1.07 | 0.32 | 0.00081 | 0.010048 |
| K08986 | -0.83 | 0.24 | 0.000803 | 0.010048 |
| K10763 | -1.31 | 0.39 | 0.000826 | 0.010048 |
| K13638 | -1.42 | 0.42 | 0.000815 | 0.010048 |
| K16138 | -1.04 | 0.31 | 0.000814 | 0.010048 |
| K19048 | -0.92 | 0.27 | 0.000827 | 0.010048 |
| K22014 | -0.78 | 0.23 | 0.000813 | 0.010048 |
| K08302 | 1.51 | 0.45 | 0.00083 | 0.010062 |
| K01720 | -1.16 | 0.34 | 0.00084 | 0.010068 |
| K03314 | -1.18 | 0.35 | 0.000835 | 0.010068 |
| K09161 | -1.14 | 0.34 | 0.000839 | 0.010068 |
| K09908 | -1.15 | 0.34 | 0.000841 | 0.010068 |
| K06214 | -1.07 | 0.32 | 0.00085 | 0.010142 |
| K05939 | -0.99 | 0.29 | 0.000864 | 0.01027 |
| K09779 | 1.32 | 0.39 | 0.000865 | 0.01027 |
| K08161 | -1.12 | 0.33 | 0.000868 | 0.010276 |
| K18831 | -1.28 | 0.38 | 0.000896 | 0.010569 |
| K01087 | -1.08 | 0.32 | 0.000908 | 0.010592 |
| K06145 | -1.02 | 0.30 | 0.000908 | 0.010592 |
| K06205 | -1.14 | 0.34 | 0.000904 | 0.010592 |
| K08279 | -1.22 | 0.36 | 0.000907 | 0.010592 |
| K19270 | -1.17 | 0.35 | 0.000914 | 0.010633 |
| K00574 | -1.24 | 0.37 | 0.000926 | 0.010718 |
| K03390 | -0.97 | 0.29 | 0.00093 | 0.010718 |
| K07038 | -1.17 | 0.35 | 0.000929 | 0.010718 |
| K03636 | -1.15 | 0.34 | 0.000934 | 0.010739 |
| K22041 | -1.06 | 0.32 | 0.000949 | 0.01088 |
| K03807 | -1.11 | 0.33 | 0.000961 | 0.010898 |
| K07473 | 0.58 | 0.17 | 0.000954 | 0.010898 |
| K10016 | -1.10 | 0.33 | 0.000958 | 0.010898 |
| K16066 | -1.22 | 0.36 | 0.000961 | 0.010898 |
| K00986 | 1.04 | 0.31 | 0.000966 | 0.0109 |
| K07243 | 1.44 | 0.43 | 0.000972 | 0.0109 |
| K11382 | -1.08 | 0.32 | 0.000969 | 0.0109 |
| K15833 | -1.03 | 0.31 | 0.00097 | 0.0109 |
| K00573 | -1.27 | 0.38 | 0.000988 | 0.010955 |
| K04770 | -0.89 | 0.27 | 0.000986 | 0.010955 |
| K12299 | -1.11 | 0.33 | 0.000987 | 0.010955 |
| K22431 | -0.82 | 0.25 | 0.000987 | 0.010955 |
| K03668 | -1.10 | 0.33 | 0.000993 | 0.010976 |
| K00691 | 0.65 | 0.19 | 0.000996 | 0.01098 |
| K12056 | -0.66 | 0.20 | 0.00101 | 0.011102 |
| K02257 | -1.10 | 0.33 | 0.001013 | 0.011106 |
| K03271 | -0.87 | 0.26 | 0.001018 | 0.011136 |
| K19165 | -1.23 | 0.37 | 0.001022 | 0.011147 |
| K03761 | -1.11 | 0.33 | 0.001027 | 0.011176 |
| K02553 | -1.04 | 0.31 | 0.001034 | 0.011208 |
| K13283 | -1.20 | 0.36 | 0.001036 | 0.011208 |
| K01141 | -1.09 | 0.33 | 0.001039 | 0.011218 |
| K00242 | -1.04 | 0.31 | 0.001053 | 0.01128 |
| K00371 | -1.27 | 0.38 | 0.001059 | 0.01128 |
| K01625 | -0.66 | 0.20 | 0.001059 | 0.01128 |
| K02410 | -0.71 | 0.21 | 0.001055 | 0.01128 |
| K13641 | -1.13 | 0.34 | 0.001059 | 0.01128 |
| K07029 | 1.23 | 0.37 | 0.001088 | 0.01153 |
| K09890 | -0.99 | 0.30 | 0.001088 | 0.01153 |
| K07261 | -0.96 | 0.29 | 0.00111 | 0.011703 |
| K08299 | -1.30 | 0.39 | 0.001108 | 0.011703 |
| K21084 | -0.98 | 0.30 | 0.001121 | 0.011784 |
| K06195 | -1.15 | 0.35 | 0.001126 | 0.011805 |
| K01682 | -1.04 | 0.32 | 0.001132 | 0.011835 |
| K06934 | 1.14 | 0.35 | 0.001135 | 0.011838 |
| K09023 | -1.02 | 0.31 | 0.001139 | 0.011855 |
| K00094 | -1.04 | 0.32 | 0.001147 | 0.011875 |
| K18220 | -0.58 | 0.18 | 0.001146 | 0.011875 |
| K00389 | -1.27 | 0.39 | 0.001167 | 0.01204 |
| K13918 | -1.04 | 0.32 | 0.001169 | 0.01204 |
| K20117 | 1.10 | 0.34 | 0.001175 | 0.012043 |
| K20118 | 1.10 | 0.34 | 0.001175 | 0.012043 |
| K07034 | -0.93 | 0.28 | 0.001178 | 0.012043 |
| K02116 | -0.98 | 0.30 | 0.001187 | 0.012096 |
| K00881 | -1.11 | 0.34 | 0.001211 | 0.012159 |
| K01061 | -1.02 | 0.31 | 0.001211 | 0.012159 |
| K08305 | -0.93 | 0.28 | 0.001209 | 0.012159 |
| K08326 | -1.08 | 0.33 | 0.001204 | 0.012159 |
| K09889 | -1.01 | 0.31 | 0.001199 | 0.012159 |
| K17938 | -0.99 | 0.30 | 0.001209 | 0.012159 |
| K01419 | -1.08 | 0.33 | 0.001226 | 0.012281 |
| K09898 | -1.22 | 0.37 | 0.001234 | 0.012333 |
| K03675 | -0.95 | 0.29 | 0.001248 | 0.012442 |
| K07245 | -0.80 | 0.25 | 0.001254 | 0.012472 |
| K06411 | -1.11 | 0.34 | 0.001266 | 0.012559 |
| K07113 | -1.04 | 0.32 | 0.001272 | 0.012591 |
| K00694 | -1.32 | 0.41 | 0.001286 | 0.012614 |
| K01034 | -0.93 | 0.29 | 0.001295 | 0.012614 |
| K03777 | -0.87 | 0.27 | 0.001288 | 0.012614 |
| K04033 | -1.24 | 0.38 | 0.001294 | 0.012614 |
| K07054 | -1.00 | 0.31 | 0.001297 | 0.012614 |
| K09971 | -0.99 | 0.30 | 0.001293 | 0.012614 |
| K11183 | -1.03 | 0.32 | 0.001285 | 0.012614 |
| K06075 | -1.11 | 0.34 | 0.001301 | 0.012627 |
| K00892 | -1.03 | 0.32 | 0.001321 | 0.012724 |
| K02686 | -1.19 | 0.37 | 0.001331 | 0.012724 |
| K03181 | -1.26 | 0.39 | 0.001321 | 0.012724 |
| K03531 | 0.72 | 0.22 | 0.001336 | 0.012724 |
| K08722 | 1.15 | 0.35 | 0.001334 | 0.012724 |
| K09024 | -1.19 | 0.37 | 0.001337 | 0.012724 |
| K09697 | -1.05 | 0.33 | 0.001339 | 0.012724 |
| K18011 | -0.80 | 0.25 | 0.001339 | 0.012724 |
| K19139 | 0.49 | 0.15 | 0.001337 | 0.012724 |
| K02012 | -1.04 | 0.32 | 0.001362 | 0.01288 |
| K02299 | -1.02 | 0.32 | 0.00136 | 0.01288 |
| K01035 | -0.64 | 0.20 | 0.001376 | 0.012923 |
| K16052 | 1.45 | 0.45 | 0.001375 | 0.012923 |
| K19228 | -1.08 | 0.33 | 0.001374 | 0.012923 |
| K03823 | 0.64 | 0.20 | 0.001395 | 0.013036 |
| K09892 | -0.97 | 0.30 | 0.001394 | 0.013036 |
| K13643 | -1.14 | 0.35 | 0.001401 | 0.013064 |
| K19167 | -0.87 | 0.27 | 0.001405 | 0.013075 |
| K01601 | -1.14 | 0.35 | 0.001413 | 0.013082 |
| K02314 | 0.63 | 0.19 | 0.001412 | 0.013082 |
| K07702 | -1.05 | 0.33 | 0.001416 | 0.013082 |
| K03633 | -1.15 | 0.36 | 0.001431 | 0.013163 |
| K12152 | -0.86 | 0.27 | 0.00143 | 0.013163 |
| K03578 | -1.17 | 0.36 | 0.001442 | 0.013215 |
| K08225 | -1.21 | 0.38 | 0.001443 | 0.013215 |
| K03769 | -1.16 | 0.36 | 0.001464 | 0.0133 |
| K07666 | -1.04 | 0.32 | 0.001471 | 0.0133 |
| K10973 | -1.11 | 0.34 | 0.001461 | 0.0133 |
| K20862 | -1.00 | 0.31 | 0.001471 | 0.0133 |
| K21739 | -1.14 | 0.35 | 0.001472 | 0.0133 |
| K21742 | -1.16 | 0.36 | 0.001459 | 0.0133 |
| K14170 | 0.84 | 0.26 | 0.001482 | 0.013357 |
| K09021 | -1.11 | 0.35 | 0.001491 | 0.013408 |
| K06175 | -0.87 | 0.27 | 0.001505 | 0.013507 |
| K22051 | -1.22 | 0.38 | 0.001511 | 0.013532 |
| K07703 | -1.15 | 0.36 | 0.001515 | 0.013538 |
| K18012 | -0.81 | 0.25 | 0.001536 | 0.013696 |
| K03591 | -1.05 | 0.33 | 0.001549 | 0.013719 |
| K09823 | -1.03 | 0.32 | 0.001546 | 0.013719 |
| K19688 | -0.88 | 0.28 | 0.001545 | 0.013719 |
| K07248 | -0.96 | 0.30 | 0.001564 | 0.013819 |
| K02682 | -1.17 | 0.37 | 0.00157 | 0.013846 |
| K01595 | -1.02 | 0.32 | 0.001597 | 0.01398 |
| K04067 | -0.96 | 0.30 | 0.001591 | 0.01398 |
| K12961 | -0.95 | 0.30 | 0.001596 | 0.01398 |
| K21470 | -0.91 | 0.29 | 0.001599 | 0.01398 |
| K02778 | -1.20 | 0.38 | 0.001619 | 0.01412 |
| K20461 | 1.30 | 0.41 | 0.00164 | 0.014276 |
| K02297 | -1.10 | 0.35 | 0.001653 | 0.014322 |
| K07673 | -0.88 | 0.28 | 0.001651 | 0.014322 |
| K09798 | -1.12 | 0.35 | 0.001657 | 0.014327 |
| K06078 | -0.92 | 0.29 | 0.00167 | 0.01439 |
| K11738 | -1.06 | 0.33 | 0.001671 | 0.01439 |
| K02679 | -0.95 | 0.30 | 0.001676 | 0.014399 |
| K02192 | -1.23 | 0.39 | 0.00169 | 0.014457 |
| K03808 | -1.02 | 0.32 | 0.001687 | 0.014457 |
| K03485 | -1.18 | 0.37 | 0.001694 | 0.014467 |
| K04044 | -1.11 | 0.35 | 0.001701 | 0.014489 |
| K03566 | -1.04 | 0.33 | 0.00171 | 0.014537 |
| K03745 | -0.89 | 0.28 | 0.001719 | 0.014588 |
| K14187 | -1.13 | 0.36 | 0.001727 | 0.01462 |
| K08349 | -0.93 | 0.29 | 0.001731 | 0.014625 |
| K02345 | -1.06 | 0.34 | 0.001739 | 0.014658 |
| K10026 | 0.93 | 0.29 | 0.001746 | 0.014658 |
| K17722 | 1.16 | 0.37 | 0.001744 | 0.014658 |
| K03557 | -1.11 | 0.35 | 0.001752 | 0.014665 |
| K16263 | -1.16 | 0.37 | 0.001754 | 0.014665 |
| K02298 | -0.98 | 0.31 | 0.001785 | 0.014864 |
| K11933 | -0.94 | 0.30 | 0.001785 | 0.014864 |
| K09160 | -1.05 | 0.33 | 0.001796 | 0.014921 |
| K09910 | -1.21 | 0.38 | 0.001802 | 0.014929 |
| K11533 | -0.47 | 0.15 | 0.001804 | 0.014929 |
| K00228 | -0.90 | 0.28 | 0.001811 | 0.014961 |
| K02195 | -0.82 | 0.26 | 0.001827 | 0.015031 |
| K19140 | 0.50 | 0.16 | 0.001826 | 0.015031 |
| K00756 | -1.13 | 0.36 | 0.00185 | 0.015186 |
| K21029 | -1.07 | 0.34 | 0.001886 | 0.015447 |
| K04080 | -1.18 | 0.37 | 0.001922 | 0.015711 |
| K03490 | -0.95 | 0.30 | 0.00193 | 0.015748 |
| K03098 | -1.03 | 0.33 | 0.001964 | 0.015893 |
| K03586 | -1.06 | 0.34 | 0.001967 | 0.015893 |
| K16012 | -1.20 | 0.38 | 0.001964 | 0.015893 |
| K16703 | -0.90 | 0.29 | 0.001963 | 0.015893 |
| K19286 | 1.13 | 0.36 | 0.001956 | 0.015893 |
| K03721 | -0.88 | 0.28 | 0.001983 | 0.01599 |
| K18205 | 1.11 | 0.36 | 0.001989 | 0.016004 |
| K02402 | -1.06 | 0.34 | 0.002005 | 0.016039 |
| K11139 | -0.89 | 0.29 | 0.002005 | 0.016039 |
| K22650 | -0.91 | 0.29 | 0.001998 | 0.016039 |
| K16235 | 1.20 | 0.39 | 0.002015 | 0.016083 |
| K14065 | -1.09 | 0.35 | 0.00202 | 0.016096 |
| K09920 | -1.04 | 0.33 | 0.00203 | 0.016137 |
| K09158 | -1.12 | 0.36 | 0.002035 | 0.016146 |
| K06400 | -0.85 | 0.27 | 0.002039 | 0.016151 |
| K03603 | -0.94 | 0.30 | 0.002053 | 0.016193 |
| K18898 | -0.97 | 0.31 | 0.002052 | 0.016193 |
| K06980 | -1.10 | 0.35 | 0.002092 | 0.016475 |
| K20542 | -0.99 | 0.32 | 0.002099 | 0.016492 |
| K00631 | -1.00 | 0.32 | 0.002112 | 0.016494 |
| K01639 | -0.66 | 0.21 | 0.002114 | 0.016494 |
| K03972 | -0.85 | 0.27 | 0.002114 | 0.016494 |
| K10545 | -0.89 | 0.29 | 0.002115 | 0.016494 |
| K07772 | -0.97 | 0.31 | 0.002123 | 0.016514 |
| K15722 | -1.23 | 0.40 | 0.002126 | 0.016514 |
| K01139 | -1.03 | 0.33 | 0.002155 | 0.016521 |
| K05985 | 1.18 | 0.38 | 0.002138 | 0.016521 |
| K07180 | -1.07 | 0.34 | 0.002145 | 0.016521 |
| K07290 | -1.07 | 0.35 | 0.00215 | 0.016521 |
| K10004 | -1.15 | 0.37 | 0.002152 | 0.016521 |
| K21759 | -0.60 | 0.19 | 0.002135 | 0.016521 |
| K22311 | -0.62 | 0.20 | 0.00214 | 0.016521 |
| K03839 | 1.37 | 0.44 | 0.00216 | 0.016523 |
| K11106 | -1.22 | 0.39 | 0.002174 | 0.016597 |
| K02439 | -0.95 | 0.31 | 0.002182 | 0.016611 |
| K02853 | -0.71 | 0.23 | 0.002184 | 0.016611 |
| K02779 | -1.16 | 0.38 | 0.002193 | 0.016651 |
| K01355 | -0.98 | 0.32 | 0.002234 | 0.016653 |
| K02412 | -0.71 | 0.23 | 0.002229 | 0.016653 |
| K07096 | 1.00 | 0.33 | 0.002229 | 0.016653 |
| K07156 | -0.89 | 0.29 | 0.002218 | 0.016653 |
| K07400 | -1.06 | 0.34 | 0.002234 | 0.016653 |
| K07662 | -0.95 | 0.31 | 0.00221 | 0.016653 |
| K08312 | -1.15 | 0.37 | 0.002219 | 0.016653 |
| K10109 | -1.11 | 0.36 | 0.002213 | 0.016653 |
| K10538 | -1.06 | 0.34 | 0.002223 | 0.016653 |
| K12136 | -0.66 | 0.21 | 0.002205 | 0.016653 |
| K00362 | -1.13 | 0.37 | 0.00228 | 0.016783 |
| K00427 | -0.96 | 0.31 | 0.002281 | 0.016783 |
| K00627 | -0.89 | 0.29 | 0.002278 | 0.016783 |
| K01734 | -0.57 | 0.18 | 0.002289 | 0.016783 |
| K02079 | -1.18 | 0.38 | 0.002264 | 0.016783 |
| K03757 | -1.12 | 0.36 | 0.002282 | 0.016783 |
| K06144 | -1.23 | 0.40 | 0.00228 | 0.016783 |
| K12525 | -1.21 | 0.39 | 0.002287 | 0.016783 |
| K16136 | -0.94 | 0.31 | 0.002273 | 0.016783 |
| K04753 | -0.88 | 0.28 | 0.002297 | 0.01681 |
| K01577 | -0.89 | 0.29 | 0.00233 | 0.017018 |
| K19337 | -0.88 | 0.29 | 0.002334 | 0.017018 |
| K09913 | -0.96 | 0.31 | 0.002356 | 0.017149 |
| K02401 | -0.61 | 0.20 | 0.00238 | 0.017281 |
| K03665 | 0.46 | 0.15 | 0.002399 | 0.017281 |
| K03732 | -0.93 | 0.30 | 0.002388 | 0.017281 |
| K04013 | -0.83 | 0.27 | 0.002401 | 0.017281 |
| K07347 | -1.11 | 0.36 | 0.002392 | 0.017281 |
| K09793 | 1.28 | 0.42 | 0.002388 | 0.017281 |
| K10539 | -1.07 | 0.35 | 0.002404 | 0.017281 |
| K07480 | -1.52 | 0.50 | 0.002427 | 0.017384 |
| K21908 | -0.91 | 0.30 | 0.002425 | 0.017384 |
| K19229 | -0.98 | 0.32 | 0.00244 | 0.01745 |
| K15539 | -1.10 | 0.36 | 0.002447 | 0.017469 |
| K11477 | -1.02 | 0.33 | 0.002464 | 0.017556 |
| K01173 | 1.02 | 0.33 | 0.00249 | 0.017713 |
| K00230 | -0.95 | 0.31 | 0.002503 | 0.017774 |
| K21645 | -1.06 | 0.35 | 0.00251 | 0.017793 |
| K06383 | 1.00 | 0.33 | 0.002535 | 0.017936 |
| K00364 | -1.10 | 0.36 | 0.002575 | 0.017956 |
| K01222 | -0.97 | 0.32 | 0.002586 | 0.017956 |
| K03562 | -1.03 | 0.34 | 0.002577 | 0.017956 |
| K03829 | -0.54 | 0.18 | 0.00256 | 0.017956 |
| K05517 | -0.96 | 0.32 | 0.002574 | 0.017956 |
| K05887 | -0.91 | 0.30 | 0.002578 | 0.017956 |
| K06956 | 0.95 | 0.31 | 0.002587 | 0.017956 |
| K08310 | -1.03 | 0.34 | 0.002552 | 0.017956 |
| K15876 | 1.25 | 0.41 | 0.002583 | 0.017956 |
| K16326 | -0.99 | 0.32 | 0.002584 | 0.017956 |
| K19350 | -0.99 | 0.33 | 0.002555 | 0.017956 |
| K02008 | -1.00 | 0.33 | 0.002592 | 0.017963 |
| K00127 | -0.61 | 0.20 | 0.0026 | 0.017986 |
| K07306 | -0.91 | 0.30 | 0.002624 | 0.01812 |
| K15635 | 1.09 | 0.36 | 0.00263 | 0.01813 |
| K02099 | -0.94 | 0.31 | 0.002648 | 0.018131 |
| K16074 | -0.99 | 0.33 | 0.002644 | 0.018131 |
| K21746 | -0.89 | 0.29 | 0.00264 | 0.018131 |
| K22110 | -1.09 | 0.36 | 0.002638 | 0.018131 |
| K05799 | -0.58 | 0.19 | 0.002662 | 0.018196 |
| K10002 | -0.91 | 0.30 | 0.002674 | 0.018235 |
| K10010 | 1.35 | 0.45 | 0.002676 | 0.018235 |
| K03417 | -0.92 | 0.30 | 0.002692 | 0.018309 |
| K02462 | -0.95 | 0.31 | 0.002705 | 0.018335 |
| K16137 | -0.98 | 0.32 | 0.002704 | 0.018335 |
| K01175 | -1.13 | 0.37 | 0.00271 | 0.01834 |
| K07498 | -1.23 | 0.41 | 0.002731 | 0.018442 |
| K09895 | -1.02 | 0.34 | 0.002743 | 0.018442 |
| K15831 | -1.00 | 0.33 | 0.00274 | 0.018442 |
| K15836 | -0.92 | 0.30 | 0.002736 | 0.018442 |
| K02498 | -0.94 | 0.31 | 0.002761 | 0.018503 |
| K06346 | 0.72 | 0.24 | 0.002766 | 0.018503 |
| K06987 | 0.94 | 0.31 | 0.002765 | 0.018503 |
| K15828 | -0.84 | 0.28 | 0.002779 | 0.018562 |
| K03673 | -1.14 | 0.38 | 0.002823 | 0.01882 |
| K03291 | -1.08 | 0.36 | 0.002838 | 0.018856 |
| K09911 | -1.08 | 0.36 | 0.002847 | 0.018856 |
| K11074 | -0.99 | 0.33 | 0.002843 | 0.018856 |
| K12975 | -0.85 | 0.28 | 0.00284 | 0.018856 |
| K06211 | -1.02 | 0.34 | 0.002852 | 0.018856 |
| K04017 | -0.75 | 0.25 | 0.002922 | 0.019257 |
| K07643 | -1.06 | 0.35 | 0.002919 | 0.019257 |
| K01673 | 0.70 | 0.23 | 0.002947 | 0.019394 |
| K12543 | -0.51 | 0.17 | 0.00297 | 0.019513 |
| K06132 | -0.91 | 0.30 | 0.002978 | 0.019536 |
| K06917 | -0.73 | 0.24 | 0.003001 | 0.019588 |
| K11734 | -1.04 | 0.35 | 0.002994 | 0.019588 |
| K22105 | -1.14 | 0.38 | 0.002999 | 0.019588 |
| K02681 | -1.01 | 0.34 | 0.003009 | 0.019609 |
| K04774 | -0.93 | 0.31 | 0.003039 | 0.019742 |
| K21965 | -0.89 | 0.30 | 0.003036 | 0.019742 |
| K00769 | -0.87 | 0.29 | 0.003078 | 0.019917 |
| K06445 | -1.10 | 0.37 | 0.003077 | 0.019917 |
| K13695 | -0.93 | 0.31 | 0.003081 | 0.019917 |
| K01960 | 0.51 | 0.17 | 0.003123 | 0.020058 |
| K03560 | -1.11 | 0.37 | 0.003131 | 0.020058 |
| K04752 | -1.04 | 0.35 | 0.003134 | 0.020058 |
| K06006 | -1.03 | 0.35 | 0.003142 | 0.020058 |
| K06157 | -0.81 | 0.27 | 0.003146 | 0.020058 |
| K07660 | -0.59 | 0.20 | 0.003127 | 0.020058 |
| K09131 | -0.90 | 0.30 | 0.003122 | 0.020058 |
| K10972 | -0.67 | 0.22 | 0.003147 | 0.020058 |
| K13620 | -1.01 | 0.34 | 0.00312 | 0.020058 |
| K06204 | -0.99 | 0.33 | 0.003161 | 0.020072 |
| K07262 | -1.08 | 0.36 | 0.003164 | 0.020072 |
| K15834 | -1.21 | 0.41 | 0.00316 | 0.020072 |
| K01354 | 1.33 | 0.45 | 0.003216 | 0.020119 |
| K02255 | -0.87 | 0.29 | 0.003184 | 0.020119 |
| K06146 | -0.83 | 0.28 | 0.003203 | 0.020119 |
| K07251 | -0.88 | 0.29 | 0.003216 | 0.020119 |
| K07320 | -1.09 | 0.36 | 0.003203 | 0.020119 |
| K07690 | -0.88 | 0.30 | 0.00319 | 0.020119 |
| K10119 | 0.73 | 0.24 | 0.003184 | 0.020119 |
| K15777 | -0.84 | 0.28 | 0.003207 | 0.020119 |
| K17733 | -0.71 | 0.24 | 0.003195 | 0.020119 |
| K13639 | -0.93 | 0.31 | 0.003225 | 0.020139 |
| K11472 | -0.98 | 0.33 | 0.003247 | 0.020247 |
| K02588 | 1.24 | 0.42 | 0.003254 | 0.020259 |
| K03631 | 0.67 | 0.22 | 0.003272 | 0.020283 |
| K05964 | -0.99 | 0.33 | 0.003278 | 0.020283 |
| K10857 | -0.91 | 0.31 | 0.003266 | 0.020283 |
| K11201 | -1.00 | 0.34 | 0.003277 | 0.020283 |
| K15583 | -0.50 | 0.17 | 0.003284 | 0.020289 |
| K10773 | 0.32 | 0.11 | 0.003324 | 0.020508 |
| K03929 | -0.61 | 0.21 | 0.003337 | 0.020554 |
| K11745 | -1.04 | 0.35 | 0.003346 | 0.020582 |
| K13014 | -0.93 | 0.32 | 0.003366 | 0.020621 |
| K15735 | -0.87 | 0.29 | 0.003368 | 0.020621 |
| K18702 | -0.80 | 0.27 | 0.003362 | 0.020621 |
| K02572 | -0.87 | 0.29 | 0.003374 | 0.020624 |
| K06916 | -0.99 | 0.33 | 0.003388 | 0.020659 |
| K07025 | 0.71 | 0.24 | 0.00339 | 0.020659 |
| K07153 | -0.95 | 0.32 | 0.003408 | 0.020738 |
| K08163 | -1.00 | 0.34 | 0.003414 | 0.020748 |
| K13244 | -1.06 | 0.36 | 0.003422 | 0.020762 |
| K00975 | 0.30 | 0.10 | 0.003432 | 0.020794 |
| K01894 | -1.00 | 0.34 | 0.003447 | 0.020828 |
| K12507 | -0.71 | 0.24 | 0.003448 | 0.020828 |
| K00872 | -0.76 | 0.26 | 0.003493 | 0.021068 |
| K09810 | 1.24 | 0.42 | 0.003504 | 0.021104 |
| K00835 | -0.98 | 0.33 | 0.003523 | 0.021151 |
| K18890 | -0.96 | 0.32 | 0.003519 | 0.021151 |
| K07304 | -0.97 | 0.33 | 0.003535 | 0.021194 |
| K01057 | 1.15 | 0.39 | 0.003575 | 0.021391 |
| K12972 | -0.92 | 0.31 | 0.003578 | 0.021391 |
| K03686 | 0.31 | 0.11 | 0.003586 | 0.021405 |
| K01878 | -0.60 | 0.20 | 0.003594 | 0.021419 |
| K14534 | -0.68 | 0.23 | 0.003602 | 0.021437 |
| K06893 | 1.31 | 0.45 | 0.003608 | 0.021442 |
| K10806 | -0.82 | 0.28 | 0.003618 | 0.021472 |
| K06898 | -0.57 | 0.19 | 0.003632 | 0.021481 |
| K12941 | 1.25 | 0.43 | 0.003626 | 0.021481 |
| K15773 | -1.16 | 0.39 | 0.003641 | 0.021481 |
| K19268 | -0.64 | 0.22 | 0.003638 | 0.021481 |
| K00209 | 0.97 | 0.33 | 0.003668 | 0.021488 |
| K00313 | -1.09 | 0.37 | 0.003655 | 0.021488 |
| K02337 | 0.38 | 0.13 | 0.003656 | 0.021488 |
| K03810 | -0.86 | 0.29 | 0.003684 | 0.021488 |
| K07637 | -0.96 | 0.33 | 0.003679 | 0.021488 |
| K08311 | -1.22 | 0.42 | 0.003678 | 0.021488 |
| K09136 | -0.92 | 0.31 | 0.003687 | 0.021488 |
| K11474 | -0.90 | 0.31 | 0.003672 | 0.021488 |
| K14392 | -0.76 | 0.26 | 0.00369 | 0.021488 |
| K15550 | -1.29 | 0.44 | 0.003697 | 0.021495 |
| K03607 | -0.85 | 0.29 | 0.003708 | 0.02153 |
| K00832 | -0.95 | 0.32 | 0.00372 | 0.021544 |
| K08961 | 1.33 | 0.45 | 0.003721 | 0.021544 |
| K05594 | -0.91 | 0.31 | 0.003746 | 0.02166 |
| K00380 | -0.97 | 0.33 | 0.00377 | 0.021711 |
| K01560 | 1.13 | 0.38 | 0.003774 | 0.021711 |
| K03290 | -0.88 | 0.30 | 0.003777 | 0.021711 |
| K21602 | -1.01 | 0.34 | 0.003765 | 0.021711 |
| K14977 | -0.88 | 0.30 | 0.003786 | 0.021734 |
| K09970 | -0.90 | 0.31 | 0.003804 | 0.021805 |
| K12262 | -0.99 | 0.34 | 0.003833 | 0.02194 |
| K01521 | -1.08 | 0.37 | 0.003864 | 0.022086 |
| K05396 | -1.10 | 0.38 | 0.003973 | 0.022664 |
| K13893 | -0.83 | 0.29 | 0.003976 | 0.022664 |
| K06016 | 1.16 | 0.40 | 0.004002 | 0.022779 |
| K01011 | -0.92 | 0.32 | 0.004028 | 0.022811 |
| K01823 | -1.02 | 0.35 | 0.00403 | 0.022811 |
| K02494 | -0.93 | 0.32 | 0.004018 | 0.022811 |
| K04046 | -0.82 | 0.28 | 0.004021 | 0.022811 |
| K02430 | -0.93 | 0.32 | 0.004049 | 0.022821 |
| K03806 | -0.93 | 0.32 | 0.004046 | 0.022821 |
| K07665 | -0.83 | 0.29 | 0.004038 | 0.022821 |
| K03528 | -0.95 | 0.33 | 0.004067 | 0.022849 |
| K03752 | -0.89 | 0.31 | 0.004071 | 0.022849 |
| K16370 | -0.88 | 0.30 | 0.004069 | 0.022849 |
| K10778 | -0.68 | 0.23 | 0.004121 | 0.023068 |
| K17326 | 1.02 | 0.35 | 0.004118 | 0.023068 |
| K16346 | -1.00 | 0.35 | 0.004134 | 0.023106 |
| K07349 | -0.92 | 0.32 | 0.004147 | 0.023149 |
| K12500 | -1.08 | 0.37 | 0.004165 | 0.023219 |
| K07184 | -1.06 | 0.37 | 0.0042 | 0.02338 |
| K03289 | -0.97 | 0.33 | 0.004209 | 0.023386 |
| K03670 | -0.66 | 0.23 | 0.004213 | 0.023386 |
| K00373 | -0.93 | 0.32 | 0.004222 | 0.023409 |
| K12410 | 0.64 | 0.22 | 0.004247 | 0.023514 |
| K11750 | -1.17 | 0.40 | 0.004299 | 0.023769 |
| K19803 | -0.67 | 0.23 | 0.004306 | 0.023773 |
| K00113 | -0.96 | 0.33 | 0.004321 | 0.023795 |
| K09712 | -0.75 | 0.26 | 0.004318 | 0.023795 |
| K03643 | -1.06 | 0.37 | 0.004338 | 0.023855 |
| K02336 | -0.78 | 0.27 | 0.00436 | 0.02393 |
| K08314 | -0.84 | 0.29 | 0.004364 | 0.02393 |
| K03618 | -0.95 | 0.33 | 0.004388 | 0.024015 |
| K08309 | -1.06 | 0.37 | 0.004397 | 0.024015 |
| K13636 | -0.89 | 0.31 | 0.004397 | 0.024015 |
| K01941 | -0.99 | 0.34 | 0.004432 | 0.024062 |
| K03478 | -0.84 | 0.29 | 0.004447 | 0.024062 |
| K06222 | -1.02 | 0.35 | 0.004439 | 0.024062 |
| K07457 | 1.42 | 0.49 | 0.004444 | 0.024062 |
| K07709 | -0.92 | 0.32 | 0.004445 | 0.024062 |
| K16090 | -0.71 | 0.25 | 0.004446 | 0.024062 |
| K19131 | -0.52 | 0.18 | 0.004427 | 0.024062 |
| K02300 | -0.86 | 0.30 | 0.004471 | 0.024092 |
| K03077 | 0.43 | 0.15 | 0.004469 | 0.024092 |
| K11104 | -1.25 | 0.43 | 0.004469 | 0.024092 |
| K07149 | 0.98 | 0.34 | 0.004526 | 0.024357 |
| K01146 | -0.67 | 0.23 | 0.004532 | 0.024359 |
| K01716 | -1.09 | 0.38 | 0.004555 | 0.02445 |
| K14061 | -0.86 | 0.30 | 0.004566 | 0.024477 |
| K08276 | -0.80 | 0.28 | 0.00458 | 0.024506 |
| K09777 | -0.76 | 0.27 | 0.004589 | 0.024506 |
| K13821 | -0.90 | 0.32 | 0.00459 | 0.024506 |
| K01816 | -0.96 | 0.34 | 0.004597 | 0.024511 |
| K02403 | -0.95 | 0.33 | 0.004615 | 0.02454 |
| K06938 | -0.80 | 0.28 | 0.004613 | 0.02454 |
| K02744 | 1.02 | 0.36 | 0.004625 | 0.024565 |
| K04061 | -0.97 | 0.34 | 0.00466 | 0.024719 |
| K02425 | -0.79 | 0.28 | 0.004675 | 0.024731 |
| K07659 | -1.12 | 0.39 | 0.004672 | 0.024731 |
| K11741 | -0.98 | 0.34 | 0.004729 | 0.024977 |
| K11934 | -1.01 | 0.35 | 0.004734 | 0.024977 |
| K14348 | -0.84 | 0.30 | 0.004775 | 0.025163 |
| K07310 | -0.77 | 0.27 | 0.004792 | 0.025202 |
| K21901 | -0.85 | 0.30 | 0.004795 | 0.025202 |
| K19052 | 1.19 | 0.42 | 0.004802 | 0.025208 |
| K18997 | -0.88 | 0.31 | 0.004812 | 0.025228 |
| K01708 | -1.10 | 0.39 | 0.004828 | 0.025249 |
| K03706 | -0.77 | 0.27 | 0.004829 | 0.025249 |
| K01690 | -0.88 | 0.31 | 0.004884 | 0.025307 |
| K01766 | -0.75 | 0.26 | 0.00489 | 0.025307 |
| K02199 | -0.79 | 0.28 | 0.004854 | 0.025307 |
| K08304 | -1.00 | 0.35 | 0.004864 | 0.025307 |
| K09471 | -0.62 | 0.22 | 0.004851 | 0.025307 |
| K10038 | -1.04 | 0.37 | 0.004872 | 0.025307 |
| K18013 | -0.90 | 0.32 | 0.004888 | 0.025307 |
| K19777 | -0.87 | 0.31 | 0.004879 | 0.025307 |
| K06878 | -0.92 | 0.33 | 0.004918 | 0.02541 |
| K08277 | -1.00 | 0.35 | 0.004923 | 0.02541 |
| K01423 | -0.78 | 0.27 | 0.004931 | 0.025424 |
| K12296 | -1.04 | 0.37 | 0.004952 | 0.025498 |
| K14054 | -1.06 | 0.37 | 0.005008 | 0.02575 |
| K07289 | -0.70 | 0.25 | 0.005029 | 0.02576 |
| K10011 | -0.78 | 0.28 | 0.005036 | 0.02576 |
| K10110 | -1.01 | 0.36 | 0.005037 | 0.02576 |
| K11203 | -0.94 | 0.33 | 0.005032 | 0.02576 |
| K11747 | -1.21 | 0.43 | 0.005041 | 0.02576 |
| K05834 | -1.01 | 0.36 | 0.005055 | 0.025796 |
| K11938 | -0.91 | 0.32 | 0.005077 | 0.025844 |
| K15547 | -0.88 | 0.31 | 0.005072 | 0.025844 |
| K01192 | 1.05 | 0.37 | 0.005153 | 0.025889 |
| K03076 | 0.45 | 0.16 | 0.005104 | 0.025889 |
| K05878 | 0.89 | 0.31 | 0.005125 | 0.025889 |
| K07481 | -1.30 | 0.46 | 0.005152 | 0.025889 |
| K07676 | -1.00 | 0.35 | 0.005156 | 0.025889 |
| K10192 | 1.10 | 0.39 | 0.005113 | 0.025889 |
| K10555 | -0.78 | 0.28 | 0.00514 | 0.025889 |
| K11258 | -0.93 | 0.33 | 0.005133 | 0.025889 |
| K11737 | -0.85 | 0.30 | 0.005117 | 0.025889 |
| K12371 | -1.01 | 0.36 | 0.005099 | 0.025889 |
| K19235 | -0.80 | 0.28 | 0.00513 | 0.025889 |
| K05839 | -0.87 | 0.31 | 0.005166 | 0.025907 |
| K06281 | -0.89 | 0.32 | 0.005189 | 0.025926 |
| K07674 | -0.69 | 0.25 | 0.005195 | 0.025926 |
| K08679 | 1.06 | 0.37 | 0.005192 | 0.025926 |
| K12660 | -1.01 | 0.36 | 0.005186 | 0.025926 |
| K00246 | -0.93 | 0.33 | 0.005218 | 0.025977 |
| K02535 | -1.16 | 0.41 | 0.005215 | 0.025977 |
| K05803 | -0.95 | 0.34 | 0.005225 | 0.025977 |
| K00428 | -0.80 | 0.29 | 0.005263 | 0.026135 |
| K00266 | 0.43 | 0.15 | 0.005277 | 0.026172 |
| K06149 | -0.98 | 0.35 | 0.005287 | 0.026188 |
| K14731 | -0.69 | 0.25 | 0.005311 | 0.026275 |
| K02415 | -0.99 | 0.35 | 0.005341 | 0.026292 |
| K05368 | -1.02 | 0.36 | 0.005347 | 0.026292 |
| K07246 | -0.63 | 0.22 | 0.005334 | 0.026292 |
| K16088 | -0.79 | 0.28 | 0.005341 | 0.026292 |
| K20541 | -0.81 | 0.29 | 0.005323 | 0.026292 |
| K02790 | -1.12 | 0.40 | 0.005395 | 0.026338 |
| K02791 | -1.12 | 0.40 | 0.005395 | 0.026338 |
| K03119 | -1.10 | 0.39 | 0.005371 | 0.026338 |
| K03971 | -0.89 | 0.32 | 0.005394 | 0.026338 |
| K09933 | -1.03 | 0.37 | 0.005367 | 0.026338 |
| K15531 | 1.21 | 0.43 | 0.00539 | 0.026338 |
| K00855 | -0.95 | 0.34 | 0.005437 | 0.026508 |
| K08172 | -0.80 | 0.29 | 0.005447 | 0.026525 |
| K03813 | -0.92 | 0.33 | 0.005458 | 0.026546 |
| K05337 | -0.84 | 0.30 | 0.005467 | 0.026559 |
| K07386 | 0.99 | 0.35 | 0.005496 | 0.026607 |
| K08319 | -0.79 | 0.28 | 0.005488 | 0.026607 |
| K11735 | -0.71 | 0.25 | 0.005496 | 0.026607 |
| K07679 | -0.84 | 0.30 | 0.005512 | 0.02665 |
| K00528 | 0.74 | 0.26 | 0.00554 | 0.026754 |
| K07738 | 0.39 | 0.14 | 0.005572 | 0.026879 |
| K02564 | 0.32 | 0.11 | 0.005581 | 0.026888 |
| K03563 | -0.60 | 0.21 | 0.005625 | 0.027047 |
| K06221 | -1.01 | 0.36 | 0.005634 | 0.027047 |
| K11209 | -0.87 | 0.31 | 0.005634 | 0.027047 |
| K07269 | -0.86 | 0.31 | 0.005665 | 0.027163 |
| K07000 | -1.10 | 0.39 | 0.005674 | 0.027177 |
| K01571 | -0.74 | 0.27 | 0.005709 | 0.027245 |
| K07798 | -1.03 | 0.37 | 0.005705 | 0.027245 |
| K11103 | -1.14 | 0.41 | 0.005703 | 0.027245 |
| K02441 | -0.85 | 0.30 | 0.005736 | 0.027344 |
| K01457 | -1.03 | 0.37 | 0.005751 | 0.027348 |
| K03775 | 0.87 | 0.31 | 0.005748 | 0.027348 |
| K12151 | -1.05 | 0.38 | 0.005759 | 0.027354 |
| K00370 | -1.09 | 0.39 | 0.005776 | 0.027406 |
| K07345 | -0.59 | 0.21 | 0.005796 | 0.027465 |
| K21572 | 1.93 | 0.69 | 0.005804 | 0.027474 |
| K01154 | 0.64 | 0.23 | 0.005821 | 0.027521 |
| K10017 | -0.89 | 0.32 | 0.005906 | 0.027889 |
| K02387 | -0.97 | 0.35 | 0.005948 | 0.027992 |
| K02570 | -0.93 | 0.34 | 0.005942 | 0.027992 |
| K03184 | -0.98 | 0.35 | 0.005948 | 0.027992 |
| K02385 | -0.67 | 0.24 | 0.005957 | 0.027999 |
| K02781 | 0.87 | 0.31 | 0.005984 | 0.028095 |
| K17716 | 1.09 | 0.39 | 0.005994 | 0.028109 |
| K08162 | -0.87 | 0.31 | 0.006014 | 0.028171 |
| K11932 | -0.94 | 0.34 | 0.006036 | 0.02824 |
| K03477 | -0.81 | 0.29 | 0.006095 | 0.028482 |
| K00245 | -0.97 | 0.35 | 0.006231 | 0.028518 |
| K00998 | -0.88 | 0.32 | 0.006178 | 0.028518 |
| K01042 | -1.06 | 0.38 | 0.006237 | 0.028518 |
| K01208 | -0.93 | 0.34 | 0.006216 | 0.028518 |
| K01990 | 0.27 | 0.10 | 0.006242 | 0.028518 |
| K02761 | -1.10 | 0.40 | 0.006229 | 0.028518 |
| K03071 | -0.94 | 0.34 | 0.006182 | 0.028518 |
| K03554 | -0.93 | 0.34 | 0.006138 | 0.028518 |
| K03746 | -0.66 | 0.24 | 0.006175 | 0.028518 |
| K03836 | -0.78 | 0.28 | 0.006249 | 0.028518 |
| K04016 | -0.98 | 0.35 | 0.006209 | 0.028518 |
| K05776 | -0.75 | 0.27 | 0.006154 | 0.028518 |
| K06867 | -1.03 | 0.37 | 0.006199 | 0.028518 |
| K07300 | -0.87 | 0.32 | 0.00623 | 0.028518 |
| K07443 | -0.64 | 0.23 | 0.006236 | 0.028518 |
| K07782 | -0.83 | 0.30 | 0.006168 | 0.028518 |
| K10551 | -0.94 | 0.34 | 0.006241 | 0.028518 |
| K13919 | -0.65 | 0.24 | 0.006226 | 0.028518 |
| K14064 | -0.71 | 0.26 | 0.00625 | 0.028518 |
| K19230 | -1.07 | 0.39 | 0.006197 | 0.028518 |
| K21966 | -0.72 | 0.26 | 0.006166 | 0.028518 |
| K04081 | -1.00 | 0.36 | 0.006276 | 0.028603 |
| K01588 | 0.41 | 0.15 | 0.006346 | 0.02889 |
| K02248 | -0.85 | 0.31 | 0.006361 | 0.028894 |
| K07346 | -0.70 | 0.25 | 0.006357 | 0.028894 |
| K20861 | -0.75 | 0.27 | 0.006404 | 0.029053 |
| K02521 | -0.86 | 0.31 | 0.006445 | 0.029161 |
| K07039 | -0.83 | 0.30 | 0.006449 | 0.029161 |
| K08996 | -0.94 | 0.34 | 0.006447 | 0.029161 |
| K09994 | -0.74 | 0.27 | 0.006495 | 0.029301 |
| K18922 | -1.12 | 0.41 | 0.006494 | 0.029301 |
| K02062 | -0.97 | 0.35 | 0.006502 | 0.029304 |
| K02784 | -0.99 | 0.36 | 0.00651 | 0.029304 |
| K15125 | -0.65 | 0.24 | 0.006517 | 0.029304 |
| K02182 | -0.72 | 0.26 | 0.00654 | 0.029361 |
| K04020 | -0.74 | 0.27 | 0.006544 | 0.029361 |
| K17992 | -0.68 | 0.25 | 0.006599 | 0.029573 |
| K09729 | -0.56 | 0.21 | 0.006619 | 0.029632 |
| K07236 | -1.01 | 0.37 | 0.006648 | 0.029694 |
| K07459 | -0.93 | 0.34 | 0.006644 | 0.029694 |
| K01715 | 0.95 | 0.35 | 0.006672 | 0.029759 |
| K03789 | 0.58 | 0.21 | 0.006677 | 0.029759 |
| K22304 | -0.39 | 0.14 | 0.006705 | 0.029819 |
| K23003 | 1.22 | 0.44 | 0.006705 | 0.029819 |
| K09017 | -0.77 | 0.28 | 0.006724 | 0.029838 |
| K09803 | 1.38 | 0.50 | 0.00672 | 0.029838 |
| K12678 | -0.87 | 0.32 | 0.00674 | 0.029875 |
| K08315 | -0.96 | 0.35 | 0.006759 | 0.029927 |
| K07722 | -0.79 | 0.29 | 0.006803 | 0.030088 |
| K11537 | -0.85 | 0.31 | 0.006861 | 0.030315 |
| K15524 | -0.64 | 0.23 | 0.006878 | 0.030353 |
| K02821 | 0.83 | 0.30 | 0.00691 | 0.030431 |
| K08350 | -0.69 | 0.25 | 0.006903 | 0.030431 |
| K05540 | -0.85 | 0.31 | 0.006949 | 0.03051 |
| K18840 | -0.80 | 0.30 | 0.00695 | 0.03051 |
| K22010 | -0.65 | 0.24 | 0.006951 | 0.03051 |
| K00112 | -0.67 | 0.25 | 0.006976 | 0.030589 |
| K07147 | -0.80 | 0.29 | 0.00706 | 0.030924 |
| K01150 | -0.83 | 0.31 | 0.007079 | 0.030972 |
| K03804 | -1.00 | 0.37 | 0.007105 | 0.030983 |
| K05880 | -0.70 | 0.26 | 0.007092 | 0.030983 |
| K19294 | -0.72 | 0.26 | 0.0071 | 0.030983 |
| K00101 | -0.83 | 0.31 | 0.007172 | 0.031156 |
| K06140 | -0.85 | 0.31 | 0.007166 | 0.031156 |
| K09696 | -0.73 | 0.27 | 0.007181 | 0.031156 |
| K15549 | -0.63 | 0.23 | 0.007164 | 0.031156 |
| K21088 | -0.70 | 0.26 | 0.007183 | 0.031156 |
| K00087 | 1.34 | 0.49 | 0.007222 | 0.031184 |
| K06039 | -0.92 | 0.34 | 0.007212 | 0.031184 |
| K08256 | -0.41 | 0.15 | 0.007217 | 0.031184 |
| K10108 | -1.35 | 0.50 | 0.007235 | 0.031184 |
| K10544 | -0.85 | 0.31 | 0.007234 | 0.031184 |
| K15552 | -1.10 | 0.41 | 0.007206 | 0.031184 |
| K02485 | -0.74 | 0.27 | 0.007247 | 0.0312 |
| K06994 | -0.38 | 0.14 | 0.007268 | 0.031256 |
| K18800 | -0.96 | 0.35 | 0.007279 | 0.031271 |
| K09857 | -0.69 | 0.25 | 0.007306 | 0.031357 |
| K02419 | -0.71 | 0.26 | 0.007368 | 0.031454 |
| K04015 | -1.02 | 0.38 | 0.007354 | 0.031454 |
| K05596 | -1.09 | 0.40 | 0.007362 | 0.031454 |
| K07757 | -0.90 | 0.33 | 0.007368 | 0.031454 |
| K21963 | -0.94 | 0.35 | 0.007366 | 0.031454 |
| K09909 | -0.81 | 0.30 | 0.007377 | 0.031458 |
| K00641 | 0.42 | 0.15 | 0.00742 | 0.031608 |
| K00138 | -0.93 | 0.35 | 0.007478 | 0.031757 |
| K02503 | 0.64 | 0.24 | 0.007481 | 0.031757 |
| K04025 | -1.02 | 0.38 | 0.007493 | 0.031757 |
| K04775 | -0.67 | 0.25 | 0.007516 | 0.031757 |
| K05516 | -0.81 | 0.30 | 0.007522 | 0.031757 |
| K06080 | -0.87 | 0.32 | 0.007522 | 0.031757 |
| K06899 | -0.73 | 0.27 | 0.007505 | 0.031757 |
| K07800 | 0.81 | 0.30 | 0.007525 | 0.031757 |
| K21964 | -0.88 | 0.33 | 0.007479 | 0.031757 |
| K15829 | -0.96 | 0.36 | 0.007541 | 0.03179 |
| K05526 | -0.92 | 0.34 | 0.007572 | 0.031886 |
| K03747 | -1.01 | 0.37 | 0.007646 | 0.032167 |
| K04754 | -0.86 | 0.32 | 0.007725 | 0.032396 |
| K07648 | -0.83 | 0.31 | 0.007724 | 0.032396 |
| K08322 | -0.98 | 0.36 | 0.007711 | 0.032396 |
| K00879 | -1.00 | 0.37 | 0.007734 | 0.0324 |
| K01667 | 1.05 | 0.39 | 0.007776 | 0.032545 |
| K07661 | -0.83 | 0.31 | 0.007808 | 0.032644 |
| K06159 | -0.83 | 0.31 | 0.007855 | 0.032808 |
| K07277 | 1.13 | 0.42 | 0.007889 | 0.032915 |
| K01581 | -1.02 | 0.38 | 0.00796 | 0.033141 |
| K07708 | -1.01 | 0.38 | 0.00796 | 0.033141 |
| K04652 | -0.62 | 0.23 | 0.007973 | 0.033162 |
| K02363 | -0.83 | 0.31 | 0.008036 | 0.033389 |
| K13938 | -0.88 | 0.33 | 0.008045 | 0.033394 |
| K16148 | 1.09 | 0.41 | 0.008066 | 0.033447 |
| K02841 | -0.89 | 0.33 | 0.008104 | 0.033567 |
| K03893 | -0.74 | 0.28 | 0.008129 | 0.03364 |
| K07309 | -0.99 | 0.37 | 0.0082 | 0.033896 |
| K01665 | 0.99 | 0.37 | 0.008219 | 0.03394 |
| K00527 | -0.66 | 0.25 | 0.008258 | 0.03399 |
| K03782 | -0.89 | 0.33 | 0.008273 | 0.03399 |
| K07713 | 1.11 | 0.42 | 0.008246 | 0.03399 |
| K16907 | -0.76 | 0.29 | 0.008266 | 0.03399 |
| K21573 | 1.28 | 0.48 | 0.008268 | 0.03399 |
| K07812 | -0.92 | 0.35 | 0.008331 | 0.034196 |
| K02404 | -0.99 | 0.37 | 0.008359 | 0.034276 |
| K00194 | 0.94 | 0.35 | 0.008405 | 0.034358 |
| K02504 | -0.77 | 0.29 | 0.008398 | 0.034358 |
| K18446 | -0.85 | 0.32 | 0.008403 | 0.034358 |
| K01659 | -0.79 | 0.30 | 0.008427 | 0.034414 |
| K01953 | -0.53 | 0.20 | 0.008482 | 0.034604 |
| K03837 | -0.82 | 0.31 | 0.00851 | 0.034681 |
| K04066 | 0.67 | 0.25 | 0.008537 | 0.034759 |
| K01727 | -0.61 | 0.23 | 0.008569 | 0.034853 |
| K03592 | -1.05 | 0.39 | 0.00858 | 0.034864 |
| K08227 | -0.85 | 0.32 | 0.008608 | 0.034941 |
| K08484 | -0.85 | 0.32 | 0.00863 | 0.034996 |
| K10189 | -0.83 | 0.31 | 0.008656 | 0.035065 |
| K17865 | -0.61 | 0.23 | 0.00868 | 0.035127 |
| K12582 | -0.76 | 0.29 | 0.008713 | 0.035227 |
| K00096 | 1.06 | 0.40 | 0.008736 | 0.035278 |
| K08297 | -1.08 | 0.41 | 0.008743 | 0.035278 |
| K21695 | -0.82 | 0.31 | 0.008771 | 0.035335 |
| K21741 | -0.85 | 0.32 | 0.008775 | 0.035335 |
| K03753 | -0.90 | 0.34 | 0.008789 | 0.035357 |
| K13256 | -0.95 | 0.36 | 0.008839 | 0.035524 |
| K09791 | -1.07 | 0.40 | 0.008853 | 0.035545 |
| K03765 | -0.70 | 0.26 | 0.008867 | 0.035565 |
| K00548 | -0.65 | 0.25 | 0.008894 | 0.035637 |
| K13628 | -1.02 | 0.39 | 0.008936 | 0.035771 |
| K03329 | -0.90 | 0.34 | 0.00895 | 0.03579 |
| K00322 | -0.80 | 0.31 | 0.008966 | 0.03582 |
| K02244 | -0.78 | 0.30 | 0.009019 | 0.035927 |
| K02461 | -0.79 | 0.30 | 0.009011 | 0.035927 |
| K07638 | -1.00 | 0.38 | 0.009006 | 0.035927 |
| K09780 | -0.80 | 0.31 | 0.009051 | 0.036018 |
| K04085 | -0.84 | 0.32 | 0.009081 | 0.0361 |
| K07644 | -0.65 | 0.25 | 0.009115 | 0.036199 |
| K01567 | 0.89 | 0.34 | 0.009135 | 0.036243 |
| K09801 | -0.80 | 0.31 | 0.009192 | 0.036436 |
| K05777 | -0.89 | 0.34 | 0.009246 | 0.036613 |
| K02846 | -0.97 | 0.37 | 0.009273 | 0.036657 |
| K21399 | -0.70 | 0.27 | 0.009275 | 0.036657 |
| K01085 | -0.76 | 0.29 | 0.009304 | 0.036736 |
| K01295 | 0.84 | 0.32 | 0.009318 | 0.036756 |
| K03717 | -0.83 | 0.32 | 0.00935 | 0.036772 |
| K03981 | -0.87 | 0.33 | 0.00934 | 0.036772 |
| K09978 | -0.75 | 0.28 | 0.009349 | 0.036772 |
| K03311 | -0.82 | 0.31 | 0.009406 | 0.036958 |
| K19267 | -0.85 | 0.32 | 0.009461 | 0.037136 |
| K02173 | 0.87 | 0.33 | 0.00948 | 0.037174 |
| K07458 | 0.92 | 0.35 | 0.009509 | 0.037229 |
| K07657 | -0.88 | 0.33 | 0.009512 | 0.037229 |
| K15548 | -0.79 | 0.30 | 0.009533 | 0.037276 |
| K01768 | 0.56 | 0.21 | 0.009583 | 0.037435 |
| K03767 | 0.58 | 0.22 | 0.009634 | 0.037597 |
| K12661 | -0.85 | 0.33 | 0.009643 | 0.037597 |
| K01224 | 0.67 | 0.26 | 0.009674 | 0.037681 |
| K09018 | -0.69 | 0.27 | 0.009692 | 0.037715 |
| K01637 | -0.86 | 0.33 | 0.009788 | 0.037761 |
| K02688 | -0.54 | 0.21 | 0.009765 | 0.037761 |
| K03516 | -0.66 | 0.25 | 0.009727 | 0.037761 |
| K07341 | -0.81 | 0.31 | 0.009777 | 0.037761 |
| K08154 | -0.91 | 0.35 | 0.009762 | 0.037761 |
| K08485 | -1.01 | 0.39 | 0.009772 | 0.037761 |
| K10118 | 0.55 | 0.21 | 0.009786 | 0.037761 |
| K11179 | -0.72 | 0.28 | 0.009766 | 0.037761 |
| K14393 | -0.75 | 0.29 | 0.00974 | 0.037761 |
| K03803 | -0.70 | 0.27 | 0.00988 | 0.03808 |
| K02852 | -0.89 | 0.34 | 0.009919 | 0.038129 |
| K05785 | -0.93 | 0.36 | 0.009921 | 0.038129 |
| K08723 | -0.79 | 0.30 | 0.009904 | 0.038129 |
| K18334 | 1.19 | 0.46 | 0.009942 | 0.038174 |
| K09914 | -0.81 | 0.31 | 0.009994 | 0.038339 |
| K06179 | -0.73 | 0.28 | 0.010013 | 0.038353 |
| K12372 | -0.98 | 0.38 | 0.010017 | 0.038353 |
| K10975 | -0.72 | 0.28 | 0.010066 | 0.038503 |
| K03762 | 0.74 | 0.28 | 0.010089 | 0.038557 |
| K03410 | -0.63 | 0.24 | 0.010129 | 0.038672 |
| K04062 | -0.89 | 0.34 | 0.010141 | 0.038681 |
| K01093 | -0.74 | 0.28 | 0.010172 | 0.038727 |
| K05365 | -1.03 | 0.40 | 0.010165 | 0.038727 |
| K00164 | -0.88 | 0.34 | 0.010193 | 0.038733 |
| K01630 | -0.84 | 0.33 | 0.010192 | 0.038733 |
| K02575 | -0.83 | 0.32 | 0.010209 | 0.038757 |
| K14260 | 0.72 | 0.28 | 0.010228 | 0.038793 |
| K06143 | 1.32 | 0.51 | 0.010243 | 0.038815 |
| K06896 | 0.95 | 0.37 | 0.010266 | 0.038856 |
| K19789 | -0.77 | 0.30 | 0.010273 | 0.038856 |
| K00885 | -0.89 | 0.35 | 0.010319 | 0.038962 |
| K03667 | -0.80 | 0.31 | 0.010321 | 0.038962 |
| K11191 | -0.86 | 0.33 | 0.010367 | 0.039066 |
| K11192 | -0.86 | 0.33 | 0.010367 | 0.039066 |
| K21574 | 1.35 | 0.52 | 0.010378 | 0.039068 |
| K02549 | -0.76 | 0.29 | 0.010421 | 0.039161 |
| K03304 | -0.66 | 0.25 | 0.010422 | 0.039161 |
| K12960 | 0.94 | 0.36 | 0.010447 | 0.03922 |
| K07796 | -0.77 | 0.30 | 0.010491 | 0.03931 |
| K12290 | -0.87 | 0.34 | 0.01049 | 0.03931 |
| K00278 | 0.72 | 0.28 | 0.010729 | 0.040129 |
| K07355 | -0.52 | 0.20 | 0.010727 | 0.040129 |
| K13926 | -0.60 | 0.23 | 0.010755 | 0.04019 |
| K11991 | 0.58 | 0.23 | 0.010766 | 0.040195 |
| K01464 | 0.93 | 0.36 | 0.010831 | 0.040334 |
| K05782 | -0.88 | 0.34 | 0.010843 | 0.040334 |
| K07356 | -0.65 | 0.25 | 0.010842 | 0.040334 |
| K07402 | -0.47 | 0.18 | 0.010834 | 0.040334 |
| K09999 | -0.73 | 0.28 | 0.01087 | 0.040397 |
| K01218 | 1.05 | 0.41 | 0.010929 | 0.040577 |
| K03273 | -1.04 | 0.41 | 0.010948 | 0.040586 |
| K07216 | -0.73 | 0.28 | 0.010961 | 0.040586 |
| K13069 | -0.90 | 0.35 | 0.010954 | 0.040586 |
| K07264 | -0.80 | 0.31 | 0.010996 | 0.040678 |
| K10556 | -0.62 | 0.24 | 0.01101 | 0.04069 |
| K02421 | -0.59 | 0.23 | 0.011063 | 0.040851 |
| K10536 | -0.70 | 0.27 | 0.011073 | 0.040852 |
| K00425 | -0.85 | 0.33 | 0.011104 | 0.040894 |
| K07724 | -0.78 | 0.30 | 0.011105 | 0.040894 |
| K00053 | 0.37 | 0.15 | 0.011163 | 0.040958 |
| K00350 | 1.20 | 0.47 | 0.011139 | 0.040958 |
| K02466 | -0.75 | 0.29 | 0.011161 | 0.040958 |
| K19303 | -0.84 | 0.33 | 0.011145 | 0.040958 |
| K04014 | -0.93 | 0.36 | 0.011189 | 0.041015 |
| K11926 | -0.69 | 0.27 | 0.011227 | 0.041119 |
| K07592 | -0.71 | 0.28 | 0.011242 | 0.041135 |
| K02560 | -0.87 | 0.34 | 0.011293 | 0.041281 |
| K03472 | -0.88 | 0.34 | 0.011302 | 0.041281 |
| K05374 | -0.72 | 0.28 | 0.011346 | 0.041341 |
| K06192 | -0.66 | 0.26 | 0.011339 | 0.041341 |
| K08308 | -0.91 | 0.36 | 0.011359 | 0.041341 |
| K16140 | -0.64 | 0.25 | 0.011351 | 0.041341 |
| K07150 | -0.69 | 0.27 | 0.011458 | 0.041663 |
| K07285 | -0.98 | 0.39 | 0.011479 | 0.0417 |
| K16925 | 1.34 | 0.53 | 0.011502 | 0.041747 |
| K03722 | -0.71 | 0.28 | 0.011515 | 0.041758 |
| K06410 | -0.68 | 0.27 | 0.011556 | 0.041869 |
| K08992 | -0.61 | 0.24 | 0.011584 | 0.041934 |
| K09167 | -0.48 | 0.19 | 0.011598 | 0.041947 |
| K00219 | -0.67 | 0.26 | 0.011696 | 0.042085 |
| K01699 | -0.77 | 0.30 | 0.011707 | 0.042085 |
| K09927 | -0.74 | 0.29 | 0.011704 | 0.042085 |
| K09967 | -1.08 | 0.43 | 0.011682 | 0.042085 |
| K13243 | -1.04 | 0.41 | 0.011664 | 0.042085 |
| K14287 | -0.69 | 0.27 | 0.011709 | 0.042085 |
| K17948 | -0.66 | 0.26 | 0.011682 | 0.042085 |
| K06074 | -0.73 | 0.29 | 0.011807 | 0.042398 |
| K00124 | -0.79 | 0.31 | 0.011915 | 0.042648 |
| K01497 | -0.85 | 0.33 | 0.011892 | 0.042648 |
| K05812 | -0.83 | 0.33 | 0.011907 | 0.042648 |
| K06880 | -0.76 | 0.30 | 0.011919 | 0.042648 |
| K00640 | -0.60 | 0.24 | 0.01199 | 0.042866 |
| K07787 | -0.66 | 0.26 | 0.012053 | 0.043051 |
| K02427 | -0.90 | 0.36 | 0.012072 | 0.043074 |
| K06918 | -0.64 | 0.25 | 0.01208 | 0.043074 |
| K00135 | -0.91 | 0.36 | 0.012124 | 0.043156 |
| K02063 | -0.98 | 0.39 | 0.012129 | 0.043156 |
| K07127 | -0.67 | 0.26 | 0.012138 | 0.043156 |
| K09774 | -0.76 | 0.30 | 0.012146 | 0.043156 |
| K03529 | 0.51 | 0.20 | 0.01221 | 0.043306 |
| K03835 | -0.82 | 0.32 | 0.012201 | 0.043306 |
| K19955 | 0.79 | 0.31 | 0.012221 | 0.043309 |
| K07710 | -0.93 | 0.37 | 0.012268 | 0.043438 |
| K19802 | 1.10 | 0.44 | 0.012283 | 0.043451 |
| K19778 | -1.02 | 0.40 | 0.012299 | 0.043471 |
| K02972 | -0.83 | 0.33 | 0.012312 | 0.04348 |
| K01271 | -0.80 | 0.32 | 0.012329 | 0.043502 |
| K07136 | -0.64 | 0.26 | 0.012355 | 0.043556 |
| K00703 | -0.55 | 0.22 | 0.012425 | 0.043732 |
| K09475 | -0.68 | 0.27 | 0.012434 | 0.043732 |
| K12137 | 0.93 | 0.37 | 0.012438 | 0.043732 |
| K00797 | -0.57 | 0.23 | 0.012547 | 0.044077 |
| K02197 | -0.78 | 0.31 | 0.012628 | 0.044175 |
| K02362 | -0.81 | 0.32 | 0.012623 | 0.044175 |
| K02474 | 0.66 | 0.26 | 0.012636 | 0.044175 |
| K04750 | -0.82 | 0.33 | 0.012623 | 0.044175 |
| K07323 | -0.73 | 0.29 | 0.01264 | 0.044175 |
| K21698 | -0.82 | 0.33 | 0.012595 | 0.044175 |
| K16013 | -0.80 | 0.32 | 0.012671 | 0.044244 |
| K21028 | -0.61 | 0.24 | 0.012686 | 0.044261 |
| K11736 | -1.05 | 0.42 | 0.012715 | 0.044323 |
| K14347 | -0.68 | 0.27 | 0.012765 | 0.044459 |
| K22720 | -0.88 | 0.35 | 0.012783 | 0.044483 |
| K00795 | -0.71 | 0.28 | 0.012799 | 0.044498 |
| K02806 | -0.90 | 0.36 | 0.012951 | 0.044952 |
| K07799 | -0.92 | 0.37 | 0.012941 | 0.044952 |
| K01950 | 0.52 | 0.21 | 0.012984 | 0.044988 |
| K20487 | -0.66 | 0.26 | 0.012982 | 0.044988 |
| K07641 | -0.81 | 0.32 | 0.01301 | 0.04504 |
| K11931 | -0.78 | 0.31 | 0.013026 | 0.045057 |
| K00880 | 1.30 | 0.52 | 0.013064 | 0.045069 |
| K03774 | -0.77 | 0.31 | 0.013063 | 0.045069 |
| K06970 | 1.11 | 0.44 | 0.013074 | 0.045069 |
| K07777 | -0.82 | 0.33 | 0.013042 | 0.045069 |
| K19354 | -0.73 | 0.29 | 0.013091 | 0.04509 |
| K05311 | -0.89 | 0.36 | 0.013171 | 0.045326 |
| K06203 | -0.80 | 0.32 | 0.013297 | 0.045679 |
| K07308 | -0.60 | 0.24 | 0.013307 | 0.045679 |
| K08092 | -0.85 | 0.34 | 0.013308 | 0.045679 |
| K01226 | -0.99 | 0.40 | 0.013328 | 0.04571 |
| K13043 | 1.16 | 0.46 | 0.013372 | 0.045822 |
| K03394 | 0.85 | 0.34 | 0.013425 | 0.045965 |
| K16209 | 1.35 | 0.54 | 0.01344 | 0.045977 |
| K00104 | 0.84 | 0.34 | 0.013504 | 0.046083 |
| K00823 | -0.54 | 0.22 | 0.013505 | 0.046083 |
| K12297 | -0.68 | 0.27 | 0.013502 | 0.046083 |
| K07019 | -0.78 | 0.31 | 0.013528 | 0.046124 |
| K00031 | -0.53 | 0.21 | 0.013602 | 0.046258 |
| K07074 | 0.40 | 0.16 | 0.0136 | 0.046258 |
| K07821 | -0.58 | 0.24 | 0.013598 | 0.046258 |
| K09473 | -0.63 | 0.25 | 0.013627 | 0.046305 |
| K02468 | -0.78 | 0.31 | 0.013664 | 0.046391 |
| K07387 | -0.88 | 0.35 | 0.013677 | 0.046398 |
| K16348 | -0.63 | 0.25 | 0.013725 | 0.04652 |
| K01096 | -0.72 | 0.29 | 0.013761 | 0.046526 |
| K06012 | 0.85 | 0.34 | 0.013749 | 0.046526 |
| K19309 | 1.01 | 0.41 | 0.013756 | 0.046526 |
| K14051 | -0.55 | 0.22 | 0.013804 | 0.046633 |
| K13888 | -0.75 | 0.30 | 0.013886 | 0.04687 |
| K00575 | -0.77 | 0.31 | 0.013945 | 0.047028 |
| K01138 | -0.83 | 0.34 | 0.014043 | 0.047321 |
| K09972 | -0.65 | 0.26 | 0.014055 | 0.047321 |
| K13979 | -0.73 | 0.30 | 0.01409 | 0.0474 |
| K00098 | -0.62 | 0.25 | 0.014146 | 0.047551 |
| K13629 | -0.81 | 0.33 | 0.014174 | 0.047602 |
| K06181 | -0.80 | 0.32 | 0.014223 | 0.047728 |
| K01916 | -0.68 | 0.28 | 0.014289 | 0.047911 |
| K02200 | -0.81 | 0.33 | 0.0144 | 0.048243 |
| K04334 | -0.72 | 0.29 | 0.014468 | 0.04839 |
| K06858 | -0.72 | 0.29 | 0.014461 | 0.04839 |
| K03336 | 0.60 | 0.25 | 0.014559 | 0.048653 |
| K18471 | 0.83 | 0.34 | 0.014598 | 0.048745 |
| K09824 | -0.85 | 0.34 | 0.01462 | 0.048777 |
| K00010 | 1.03 | 0.42 | 0.014645 | 0.048806 |
| K19506 | 0.95 | 0.38 | 0.014653 | 0.048806 |
| K01919 | -0.50 | 0.20 | 0.014755 | 0.049073 |
| K17723 | 0.81 | 0.33 | 0.014757 | 0.049073 |
| K07175 | 0.91 | 0.37 | 0.014807 | 0.049199 |
| K02523 | -0.96 | 0.39 | 0.014945 | 0.049455 |
| K03779 | -0.65 | 0.27 | 0.014937 | 0.049455 |
| K07160 | -0.59 | 0.24 | 0.014936 | 0.049455 |
| K08682 | -0.64 | 0.26 | 0.014919 | 0.049455 |
| K20534 | 0.90 | 0.37 | 0.014904 | 0.049455 |
| K07784 | -0.98 | 0.40 | 0.014958 | 0.049456 |
| K00130 | -0.69 | 0.28 | 0.014997 | 0.049544 |
| K15984 | -0.90 | 0.37 | 0.015095 | 0.049789 |
| K18657 | -0.73 | 0.30 | 0.015088 | 0.049789 |
| K16927 | 0.80 | 0.33 | 0.015208 | 0.050119 |
| K06610 | 1.14 | 0.47 | 0.015287 | 0.050298 |
| K09937 | -0.95 | 0.39 | 0.015278 | 0.050298 |
| K03974 | -0.88 | 0.36 | 0.01533 | 0.050398 |
| K12148 | -0.97 | 0.40 | 0.015358 | 0.050449 |
| K12957 | -0.67 | 0.27 | 0.015402 | 0.050536 |
| K22486 | -0.44 | 0.18 | 0.015409 | 0.050536 |
| K03640 | -0.84 | 0.35 | 0.015501 | 0.050797 |
| K01761 | 1.00 | 0.41 | 0.015552 | 0.05092 |
| K01880 | 0.39 | 0.16 | 0.015579 | 0.050968 |
| K01635 | -0.80 | 0.33 | 0.015608 | 0.051023 |
| K08984 | -0.70 | 0.29 | 0.015677 | 0.051205 |
| K07084 | -0.70 | 0.29 | 0.01574 | 0.051331 |
| K07588 | 1.44 | 0.59 | 0.015741 | 0.051331 |
| K01911 | -0.96 | 0.39 | 0.015807 | 0.051505 |
| K01627 | -0.93 | 0.38 | 0.01584 | 0.051572 |
| K02423 | -0.81 | 0.33 | 0.015884 | 0.051672 |
| K09181 | -0.76 | 0.31 | 0.015976 | 0.051933 |
| K02400 | -0.60 | 0.25 | 0.016112 | 0.052291 |
| K05708 | -0.61 | 0.25 | 0.016105 | 0.052291 |
| K13632 | -0.59 | 0.24 | 0.01613 | 0.052305 |
| K03325 | 0.83 | 0.34 | 0.016202 | 0.05247 |
| K18324 | -0.74 | 0.31 | 0.016207 | 0.05247 |
| K05790 | -0.96 | 0.40 | 0.016271 | 0.052638 |
| K09157 | 0.28 | 0.11 | 0.016355 | 0.052825 |
| K11748 | -0.96 | 0.40 | 0.016345 | 0.052825 |
| K02408 | -0.58 | 0.24 | 0.016413 | 0.052971 |
| K05814 | -1.01 | 0.42 | 0.01644 | 0.053013 |
| K11742 | -0.73 | 0.30 | 0.016456 | 0.053024 |
| K02573 | -0.67 | 0.28 | 0.016524 | 0.053201 |
| K03577 | -0.78 | 0.32 | 0.016565 | 0.05329 |
| K02855 | -0.78 | 0.32 | 0.016635 | 0.053389 |
| K05364 | 1.11 | 0.46 | 0.016624 | 0.053389 |
| K14205 | 0.61 | 0.25 | 0.016625 | 0.053389 |
| K10001 | -0.55 | 0.23 | 0.016674 | 0.05347 |
| K21974 | -0.49 | 0.20 | 0.016692 | 0.053485 |
| K09998 | -0.94 | 0.39 | 0.016767 | 0.053685 |
| K18142 | -0.98 | 0.41 | 0.016898 | 0.054061 |
| K08348 | -0.78 | 0.32 | 0.016929 | 0.054115 |
| K10124 | -0.93 | 0.39 | 0.016992 | 0.054274 |
| K13053 | -0.47 | 0.19 | 0.017052 | 0.054384 |
| K18478 | -0.67 | 0.28 | 0.017053 | 0.054384 |
| K03635 | -0.88 | 0.36 | 0.017115 | 0.054538 |
| K03634 | -0.63 | 0.26 | 0.01718 | 0.054659 |
| K15256 | -0.72 | 0.30 | 0.017176 | 0.054659 |
| K16704 | -0.62 | 0.26 | 0.017209 | 0.054677 |
| K18120 | -0.60 | 0.25 | 0.017212 | 0.054677 |
| K07237 | -0.70 | 0.29 | 0.017274 | 0.054787 |
| K15830 | -0.56 | 0.24 | 0.017261 | 0.054787 |
| K13301 | -0.76 | 0.32 | 0.017294 | 0.054807 |
| K01709 | 0.67 | 0.28 | 0.017371 | 0.055007 |
| K02843 | -0.88 | 0.37 | 0.017429 | 0.055075 |
| K03186 | 0.72 | 0.30 | 0.017408 | 0.055075 |
| K07639 | -0.77 | 0.32 | 0.017433 | 0.055075 |
| K02438 | -0.72 | 0.30 | 0.017502 | 0.055251 |
| K05606 | -0.64 | 0.27 | 0.017536 | 0.055313 |
| K02569 | -0.71 | 0.30 | 0.017761 | 0.055982 |
| K19810 | -0.61 | 0.26 | 0.017864 | 0.056219 |
| K21021 | -0.62 | 0.26 | 0.017864 | 0.056219 |
| K21637 | -0.79 | 0.33 | 0.018012 | 0.056639 |
| K08313 | -0.64 | 0.27 | 0.018049 | 0.05671 |
| K03112 | -0.57 | 0.24 | 0.018143 | 0.056961 |
| K04047 | -0.85 | 0.36 | 0.018157 | 0.056961 |
| K06895 | -0.99 | 0.41 | 0.018226 | 0.057133 |
| K08681 | 0.60 | 0.25 | 0.01832 | 0.057385 |
| K00285 | -0.75 | 0.32 | 0.018381 | 0.057451 |
| K07454 | 0.98 | 0.41 | 0.018384 | 0.057451 |
| K10254 | 0.60 | 0.25 | 0.018372 | 0.057451 |
| K03641 | -0.88 | 0.37 | 0.018423 | 0.057531 |
| K08219 | -0.70 | 0.29 | 0.018449 | 0.057566 |
| K12144 | -0.51 | 0.21 | 0.018524 | 0.057756 |
| K01421 | 0.57 | 0.24 | 0.018582 | 0.057802 |
| K02550 | -0.67 | 0.28 | 0.018581 | 0.057802 |
| K19973 | -0.70 | 0.29 | 0.018555 | 0.057802 |
| K03750 | -0.64 | 0.27 | 0.018611 | 0.057813 |
| K21948 | -0.82 | 0.35 | 0.018614 | 0.057813 |
| K01058 | -0.77 | 0.32 | 0.018683 | 0.057913 |
| K08641 | 1.21 | 0.51 | 0.018689 | 0.057913 |
| K21575 | 1.07 | 0.45 | 0.018688 | 0.057913 |
| K00116 | -0.77 | 0.32 | 0.018755 | 0.057963 |
| K01460 | -0.61 | 0.26 | 0.018757 | 0.057963 |
| K03690 | -0.74 | 0.31 | 0.018756 | 0.057963 |
| K10009 | -0.77 | 0.33 | 0.018762 | 0.057963 |
| K00690 | 0.63 | 0.27 | 0.018902 | 0.058221 |
| K02946 | 0.19 | 0.08 | 0.018932 | 0.058221 |
| K03718 | -0.82 | 0.35 | 0.018912 | 0.058221 |
| K04337 | -0.83 | 0.35 | 0.018926 | 0.058221 |
| K07263 | 1.19 | 0.50 | 0.018868 | 0.058221 |
| K18862 | -0.96 | 0.41 | 0.018906 | 0.058221 |
| K03179 | -0.83 | 0.35 | 0.01896 | 0.058263 |
| K19155 | -0.67 | 0.29 | 0.01901 | 0.058371 |
| K13891 | -0.69 | 0.29 | 0.019146 | 0.058743 |
| K09800 | -0.86 | 0.36 | 0.019202 | 0.05887 |
| K03482 | -0.50 | 0.21 | 0.019248 | 0.058928 |
| K07451 | -0.73 | 0.31 | 0.019264 | 0.058928 |
| K08301 | 0.42 | 0.18 | 0.019263 | 0.058928 |
| K16321 | -0.73 | 0.31 | 0.019286 | 0.058949 |
| K00121 | -0.80 | 0.34 | 0.019409 | 0.059239 |
| K11618 | -0.72 | 0.31 | 0.01941 | 0.059239 |
| K05835 | -0.71 | 0.30 | 0.019428 | 0.05925 |
| K09794 | -0.87 | 0.37 | 0.019515 | 0.059426 |
| K18968 | -0.78 | 0.33 | 0.019506 | 0.059426 |
| K16693 | -0.73 | 0.31 | 0.019633 | 0.059739 |
| K02196 | -0.80 | 0.34 | 0.019651 | 0.059749 |
| K05984 | -0.55 | 0.23 | 0.019721 | 0.059872 |
| K12369 | -0.81 | 0.34 | 0.019715 | 0.059872 |
| K07311 | -0.70 | 0.30 | 0.019885 | 0.060326 |
| K13953 | -0.80 | 0.34 | 0.019914 | 0.060368 |
| K19338 | -0.74 | 0.32 | 0.019989 | 0.06055 |
| K04844 | -0.98 | 0.42 | 0.020013 | 0.060577 |
| K03546 | -0.59 | 0.25 | 0.020141 | 0.060883 |
| K10985 | -0.56 | 0.24 | 0.020144 | 0.060883 |
| K01608 | -0.89 | 0.38 | 0.020231 | 0.06093 |
| K02892 | 0.17 | 0.07 | 0.020207 | 0.06093 |
| K04336 | -0.76 | 0.33 | 0.020262 | 0.06093 |
| K05710 | -0.70 | 0.30 | 0.020265 | 0.06093 |
| K07351 | -0.69 | 0.30 | 0.02024 | 0.06093 |
| K09781 | -0.66 | 0.28 | 0.020229 | 0.06093 |
| K10008 | 0.71 | 0.31 | 0.020236 | 0.06093 |
| K02805 | -0.66 | 0.28 | 0.020319 | 0.061043 |
| K03445 | -0.83 | 0.35 | 0.020333 | 0.061043 |
| K02824 | -0.69 | 0.30 | 0.020376 | 0.061129 |
| K01079 | 0.65 | 0.28 | 0.02052 | 0.061343 |
| K04064 | -0.70 | 0.30 | 0.02052 | 0.061343 |
| K09918 | -0.73 | 0.31 | 0.020523 | 0.061343 |
| K19236 | -0.67 | 0.29 | 0.020471 | 0.061343 |
| K22443 | -0.41 | 0.18 | 0.020481 | 0.061343 |
| K00867 | -0.72 | 0.31 | 0.02063 | 0.061586 |
| K03897 | -0.55 | 0.24 | 0.020666 | 0.061586 |
| K07319 | -0.67 | 0.29 | 0.020658 | 0.061586 |
| K09979 | -0.94 | 0.40 | 0.020644 | 0.061586 |
| K03113 | 0.85 | 0.37 | 0.020689 | 0.061611 |
| K05786 | -0.82 | 0.35 | 0.020714 | 0.061638 |
| K02189 | -0.57 | 0.25 | 0.020812 | 0.061841 |
| K05875 | -0.65 | 0.28 | 0.020809 | 0.061841 |
| K07714 | -0.79 | 0.34 | 0.020859 | 0.061904 |
| K14415 | 1.11 | 0.48 | 0.020864 | 0.061904 |
| K03522 | 0.85 | 0.37 | 0.021058 | 0.062387 |
| K03676 | -0.67 | 0.29 | 0.021057 | 0.062387 |
| K11076 | -0.95 | 0.41 | 0.021073 | 0.062387 |
| K01048 | -0.76 | 0.33 | 0.021143 | 0.062547 |
| K02411 | -0.62 | 0.27 | 0.021164 | 0.062564 |
| K02114 | -0.33 | 0.14 | 0.021245 | 0.06272 |
| K19611 | -0.71 | 0.30 | 0.021248 | 0.06272 |
| K07014 | -0.70 | 0.30 | 0.021302 | 0.062789 |
| K11936 | -0.84 | 0.36 | 0.021293 | 0.062789 |
| K05499 | -0.90 | 0.39 | 0.021616 | 0.063668 |
| K01089 | 0.89 | 0.39 | 0.021649 | 0.06372 |
| K12974 | -0.60 | 0.26 | 0.021678 | 0.063757 |
| K00058 | 0.37 | 0.16 | 0.021709 | 0.063802 |
| K02394 | -0.74 | 0.32 | 0.021779 | 0.063963 |
| K00948 | 0.29 | 0.12 | 0.021853 | 0.064125 |
| K16695 | -0.63 | 0.27 | 0.021866 | 0.064125 |
| K07275 | -0.63 | 0.27 | 0.021883 | 0.064127 |
| K03611 | -0.72 | 0.31 | 0.021949 | 0.064275 |
| K01612 | -0.68 | 0.29 | 0.022046 | 0.064383 |
| K01995 | -0.58 | 0.25 | 0.022039 | 0.064383 |
| K06968 | -0.80 | 0.35 | 0.022066 | 0.064383 |
| K07336 | -0.83 | 0.36 | 0.022065 | 0.064383 |
| K13012 | 0.95 | 0.41 | 0.022037 | 0.064383 |
| K07099 | -0.59 | 0.26 | 0.022148 | 0.064531 |
| K18140 | -0.71 | 0.31 | 0.022146 | 0.064531 |
| K01620 | 0.51 | 0.22 | 0.022165 | 0.064533 |
| K03819 | -0.64 | 0.28 | 0.022265 | 0.064779 |
| K18479 | -0.74 | 0.32 | 0.0223 | 0.064835 |
| K05838 | -0.77 | 0.34 | 0.022476 | 0.065299 |
| K05910 | -0.69 | 0.30 | 0.022511 | 0.065354 |
| K06190 | -0.70 | 0.30 | 0.022559 | 0.065446 |
| K09891 | -0.59 | 0.26 | 0.022615 | 0.065561 |
| K03182 | -0.90 | 0.39 | 0.022635 | 0.065564 |
| K05997 | -0.77 | 0.33 | 0.022648 | 0.065564 |
| K20480 | -0.72 | 0.32 | 0.022761 | 0.065844 |
| K03824 | -0.76 | 0.33 | 0.022814 | 0.065949 |
| K02457 | -0.75 | 0.33 | 0.022988 | 0.066406 |
| K03749 | -0.83 | 0.36 | 0.023011 | 0.066422 |
| K02554 | -0.62 | 0.27 | 0.023039 | 0.066424 |
| K22373 | 0.67 | 0.29 | 0.023044 | 0.066424 |
| K11527 | 0.71 | 0.31 | 0.023216 | 0.066873 |
| K18928 | 1.03 | 0.45 | 0.023233 | 0.066874 |
| K10543 | -0.84 | 0.37 | 0.0233 | 0.067019 |
| K00215 | 0.30 | 0.13 | 0.023545 | 0.067579 |
| K01772 | -0.66 | 0.29 | 0.023515 | 0.067579 |
| K03148 | -0.79 | 0.35 | 0.023542 | 0.067579 |
| K00108 | -0.66 | 0.29 | 0.023605 | 0.067657 |
| K15461 | -0.80 | 0.35 | 0.023602 | 0.067657 |
| K07023 | -0.58 | 0.25 | 0.02365 | 0.067737 |
| K09163 | -0.52 | 0.23 | 0.023695 | 0.067816 |
| K21975 | -0.67 | 0.30 | 0.023813 | 0.068107 |
| K13630 | -0.72 | 0.32 | 0.023872 | 0.068227 |
| K11930 | -0.80 | 0.35 | 0.023976 | 0.068476 |
| K05590 | -0.81 | 0.36 | 0.024133 | 0.068877 |
| K03335 | 0.76 | 0.34 | 0.024365 | 0.069491 |
| K16710 | -0.60 | 0.27 | 0.024513 | 0.069863 |
| K01725 | -0.52 | 0.23 | 0.024678 | 0.070282 |
| K03780 | -0.78 | 0.34 | 0.024753 | 0.070445 |
| K03530 | 0.25 | 0.11 | 0.024967 | 0.071006 |
| K05844 | -0.77 | 0.34 | 0.025026 | 0.071122 |
| K03700 | -0.51 | 0.23 | 0.025074 | 0.071209 |
| K22719 | -0.68 | 0.30 | 0.025111 | 0.071266 |
| K01817 | 0.69 | 0.31 | 0.025174 | 0.071293 |
| K12288 | -0.43 | 0.19 | 0.025162 | 0.071293 |
| K21613 | -0.76 | 0.34 | 0.025144 | 0.071293 |
| K05539 | -0.73 | 0.32 | 0.025305 | 0.071599 |
| K10831 | -0.78 | 0.35 | 0.025318 | 0.071599 |
| K11924 | -0.76 | 0.34 | 0.025335 | 0.071599 |
| K07146 | -0.56 | 0.25 | 0.025409 | 0.071757 |
| K02911 | 0.18 | 0.08 | 0.025427 | 0.071759 |
| K06905 | 0.49 | 0.22 | 0.025524 | 0.071984 |
| K02240 | -0.61 | 0.27 | 0.025607 | 0.072166 |
| K19405 | 0.57 | 0.25 | 0.025715 | 0.072421 |
| K09809 | -0.52 | 0.23 | 0.025763 | 0.072456 |
| K15545 | -0.66 | 0.29 | 0.025761 | 0.072456 |
| K04021 | -0.63 | 0.28 | 0.025881 | 0.072735 |
| K00259 | 0.89 | 0.40 | 0.02594 | 0.072774 |
| K01465 | -0.39 | 0.17 | 0.025942 | 0.072774 |
| K04784 | -0.71 | 0.32 | 0.025966 | 0.072774 |
| K11940 | -0.65 | 0.29 | 0.025964 | 0.072774 |
| K11391 | -0.82 | 0.37 | 0.025992 | 0.072796 |
| K05946 | -0.60 | 0.27 | 0.026089 | 0.073017 |
| K01255 | -0.84 | 0.38 | 0.026407 | 0.073783 |
| K02453 | -0.84 | 0.38 | 0.026418 | 0.073783 |
| K07479 | -0.67 | 0.30 | 0.026402 | 0.073783 |
| K13574 | -0.73 | 0.33 | 0.026525 | 0.074031 |
| K02011 | -0.76 | 0.34 | 0.026569 | 0.074052 |
| K11529 | 0.83 | 0.37 | 0.02656 | 0.074052 |
| K07675 | -0.78 | 0.35 | 0.026595 | 0.074069 |
| K08357 | -0.39 | 0.17 | 0.026611 | 0.074069 |
| K02064 | -0.82 | 0.37 | 0.026671 | 0.074184 |
| K03074 | -0.79 | 0.35 | 0.026697 | 0.074206 |
| K05527 | -0.80 | 0.36 | 0.02673 | 0.074246 |
| K06048 | -0.64 | 0.29 | 0.026851 | 0.07453 |
| K01201 | 0.87 | 0.39 | 0.026997 | 0.074885 |
| K01974 | -0.42 | 0.19 | 0.027017 | 0.07489 |
| K00598 | 1.14 | 0.51 | 0.027088 | 0.075034 |
| K17247 | -0.69 | 0.31 | 0.027192 | 0.075272 |
| K00111 | -0.50 | 0.23 | 0.027305 | 0.075429 |
| K01483 | -0.58 | 0.26 | 0.027285 | 0.075429 |
| K07791 | -0.84 | 0.38 | 0.0273 | 0.075429 |
| K00001 | 0.75 | 0.34 | 0.02738 | 0.075556 |
| K03072 | -0.83 | 0.38 | 0.027444 | 0.075556 |
| K04063 | -0.62 | 0.28 | 0.027436 | 0.075556 |
| K08963 | -0.76 | 0.34 | 0.027434 | 0.075556 |
| K21571 | 0.89 | 0.40 | 0.027397 | 0.075556 |
| K03674 | -1.03 | 0.46 | 0.027463 | 0.075557 |
| K07740 | -0.60 | 0.27 | 0.027743 | 0.076274 |
| K05774 | -0.75 | 0.34 | 0.027985 | 0.076887 |
| K01046 | 0.65 | 0.29 | 0.028007 | 0.076895 |
| K09118 | 0.65 | 0.30 | 0.028061 | 0.076992 |
| K08987 | -0.38 | 0.17 | 0.028106 | 0.077062 |
| K06907 | 0.74 | 0.33 | 0.028266 | 0.07745 |
| K07712 | -0.89 | 0.40 | 0.028335 | 0.077535 |
| K14055 | -0.74 | 0.33 | 0.028324 | 0.077535 |
| K05591 | -0.65 | 0.29 | 0.028389 | 0.077629 |
| K03759 | -0.68 | 0.31 | 0.028436 | 0.077704 |
| K01782 | -0.82 | 0.37 | 0.028491 | 0.077803 |
| K06929 | -0.72 | 0.33 | 0.028574 | 0.077977 |
| K02418 | -0.58 | 0.26 | 0.028702 | 0.078273 |
| K04654 | 0.82 | 0.37 | 0.028928 | 0.078837 |
| K02397 | -0.74 | 0.34 | 0.029018 | 0.078943 |
| K02854 | -0.71 | 0.32 | 0.029104 | 0.078943 |
| K06223 | 0.86 | 0.39 | 0.02906 | 0.078943 |
| K10015 | -0.87 | 0.40 | 0.02901 | 0.078943 |
| K12962 | -0.59 | 0.27 | 0.029082 | 0.078943 |
| K17899 | -0.56 | 0.26 | 0.02909 | 0.078943 |
| K18349 | 0.73 | 0.33 | 0.029098 | 0.078943 |
| K09771 | -0.65 | 0.30 | 0.029162 | 0.079048 |
| K14588 | -0.53 | 0.24 | 0.029182 | 0.079048 |
| K09900 | -0.90 | 0.41 | 0.029212 | 0.079078 |
| K09476 | -0.57 | 0.26 | 0.029505 | 0.079817 |
| K02499 | -0.54 | 0.25 | 0.029576 | 0.079941 |
| K09934 | -0.87 | 0.40 | 0.02959 | 0.079941 |
| K07507 | -0.51 | 0.24 | 0.029775 | 0.080386 |
| K06166 | -0.55 | 0.25 | 0.029815 | 0.080442 |
| K09896 | -0.74 | 0.34 | 0.02988 | 0.080508 |
| K14056 | -0.63 | 0.29 | 0.029863 | 0.080508 |
| K02856 | -0.66 | 0.30 | 0.030127 | 0.081012 |
| K13635 | -0.66 | 0.30 | 0.030125 | 0.081012 |
| K17810 | -0.64 | 0.29 | 0.030098 | 0.081012 |
| K12944 | -0.68 | 0.31 | 0.030175 | 0.081088 |
| K00884 | -0.85 | 0.39 | 0.030278 | 0.081094 |
| K01975 | -0.75 | 0.34 | 0.030263 | 0.081094 |
| K02452 | -0.80 | 0.37 | 0.030278 | 0.081094 |
| K07715 | -0.63 | 0.29 | 0.030262 | 0.081094 |
| K12149 | -0.81 | 0.37 | 0.030222 | 0.081094 |
| K00346 | 0.92 | 0.42 | 0.030424 | 0.081312 |
| K01209 | -0.43 | 0.20 | 0.030406 | 0.081312 |
| K08173 | -0.49 | 0.23 | 0.030439 | 0.081312 |
| K08300 | 0.52 | 0.24 | 0.030434 | 0.081312 |
| K02283 | 0.67 | 0.31 | 0.030864 | 0.08203 |
| K02483 | -0.88 | 0.40 | 0.030766 | 0.08203 |
| K03975 | 0.54 | 0.25 | 0.030835 | 0.08203 |
| K06926 | 0.83 | 0.38 | 0.030755 | 0.08203 |
| K07016 | -0.61 | 0.28 | 0.030856 | 0.08203 |
| K09685 | -0.61 | 0.28 | 0.030851 | 0.08203 |
| K11102 | -0.58 | 0.27 | 0.030894 | 0.08203 |
| K13747 | 0.72 | 0.33 | 0.030794 | 0.08203 |
| K15986 | 0.54 | 0.25 | 0.030911 | 0.08203 |
| K16089 | 1.03 | 0.47 | 0.030888 | 0.08203 |
| K02386 | -0.66 | 0.30 | 0.031019 | 0.082263 |
| K00068 | 0.85 | 0.39 | 0.031041 | 0.082267 |
| K03739 | -0.58 | 0.27 | 0.031134 | 0.08246 |
| K09016 | -0.38 | 0.18 | 0.0313 | 0.082845 |
| K01838 | -0.95 | 0.44 | 0.031387 | 0.083022 |
| K21973 | -0.78 | 0.36 | 0.031427 | 0.083074 |
| K09789 | 0.84 | 0.39 | 0.03151 | 0.083239 |
| K11145 | 0.65 | 0.30 | 0.031651 | 0.083557 |
| K02014 | 0.94 | 0.43 | 0.031703 | 0.083639 |
| K09915 | -0.70 | 0.32 | 0.031953 | 0.084243 |
| K03583 | -0.79 | 0.37 | 0.032088 | 0.084544 |
| K00813 | -0.69 | 0.32 | 0.032236 | 0.084879 |
| K01897 | 0.40 | 0.18 | 0.032291 | 0.084968 |
| K07124 | 0.61 | 0.29 | 0.032435 | 0.085292 |
| K06608 | -0.41 | 0.19 | 0.032487 | 0.085372 |
| K01869 | 0.20 | 0.09 | 0.032534 | 0.085385 |
| K11075 | -0.80 | 0.37 | 0.032525 | 0.085385 |
| K22605 | -0.79 | 0.37 | 0.032562 | 0.085404 |
| K00763 | 0.44 | 0.20 | 0.032634 | 0.085537 |
| K10213 | -0.78 | 0.36 | 0.032703 | 0.085663 |
| K08352 | -0.28 | 0.13 | 0.032758 | 0.085724 |
| K23016 | -0.58 | 0.27 | 0.032769 | 0.085724 |
| K06201 | 0.53 | 0.25 | 0.032844 | 0.085845 |
| K06282 | -0.52 | 0.24 | 0.032857 | 0.085845 |
| K02532 | -1.06 | 0.49 | 0.032889 | 0.085872 |
| K03926 | -0.69 | 0.32 | 0.03327 | 0.086792 |
| K04517 | 0.60 | 0.28 | 0.033284 | 0.086792 |
| K07337 | -0.67 | 0.31 | 0.033459 | 0.087192 |
| K01494 | 0.50 | 0.23 | 0.033525 | 0.087254 |
| K19000 | -0.54 | 0.25 | 0.033532 | 0.087254 |
| K19335 | -0.44 | 0.20 | 0.033548 | 0.087254 |
| K02455 | -0.72 | 0.34 | 0.033575 | 0.087268 |
| K04092 | 0.88 | 0.41 | 0.03366 | 0.087434 |
| K00912 | 0.94 | 0.44 | 0.033721 | 0.087537 |
| K00090 | -0.82 | 0.38 | 0.033814 | 0.087721 |
| K11935 | -0.64 | 0.30 | 0.033924 | 0.08795 |
| K02377 | 0.93 | 0.44 | 0.034071 | 0.08822 |
| K04018 | -0.81 | 0.38 | 0.03405 | 0.08822 |
| K06194 | -0.75 | 0.35 | 0.03412 | 0.08829 |
| K09472 | -0.46 | 0.22 | 0.034298 | 0.088692 |
| K01910 | -0.68 | 0.32 | 0.034337 | 0.088738 |
| K07121 | -0.68 | 0.32 | 0.034405 | 0.088856 |
| K00287 | 0.37 | 0.18 | 0.034565 | 0.089211 |
| K03414 | -0.71 | 0.34 | 0.034632 | 0.089327 |
| K02742 | -0.71 | 0.34 | 0.034877 | 0.089902 |
| K01879 | -0.61 | 0.29 | 0.034914 | 0.089942 |
| K02844 | -0.33 | 0.16 | 0.035017 | 0.090091 |
| K09921 | -0.77 | 0.36 | 0.034997 | 0.090091 |
| K06904 | 0.96 | 0.45 | 0.035114 | 0.090227 |
| K07781 | -0.69 | 0.32 | 0.035097 | 0.090227 |
| K14059 | -0.52 | 0.25 | 0.035188 | 0.090359 |
| K03644 | 0.72 | 0.34 | 0.035234 | 0.090395 |
| K15737 | -0.59 | 0.28 | 0.035247 | 0.090395 |
| K01937 | 0.24 | 0.11 | 0.035281 | 0.090426 |
| K07810 | -0.55 | 0.26 | 0.035637 | 0.091279 |
| K07354 | -0.53 | 0.25 | 0.035736 | 0.091476 |
| K02798 | 0.67 | 0.32 | 0.035866 | 0.091693 |
| K11941 | -0.57 | 0.27 | 0.035847 | 0.091693 |
| K02194 | -0.50 | 0.24 | 0.035893 | 0.091704 |
| K13735 | -0.50 | 0.24 | 0.036157 | 0.092321 |
| K11177 | -0.35 | 0.17 | 0.036297 | 0.09262 |
| K07001 | 0.78 | 0.37 | 0.036323 | 0.092627 |
| K07350 | -0.60 | 0.28 | 0.036407 | 0.092725 |
| K07686 | -0.75 | 0.36 | 0.036406 | 0.092725 |
| K03841 | -0.68 | 0.32 | 0.036522 | 0.092901 |
| K04760 | -0.82 | 0.39 | 0.036512 | 0.092901 |
| K20543 | -0.51 | 0.24 | 0.036605 | 0.093053 |
| K03764 | -0.79 | 0.38 | 0.036777 | 0.093432 |
| K02529 | 0.25 | 0.12 | 0.036919 | 0.093733 |
| K17234 | -0.77 | 0.37 | 0.037006 | 0.093895 |
| K02167 | -0.61 | 0.29 | 0.037274 | 0.094515 |
| K06894 | 0.89 | 0.42 | 0.037308 | 0.094543 |
| K11929 | -0.44 | 0.21 | 0.037332 | 0.094545 |
| K07776 | -0.51 | 0.25 | 0.037367 | 0.094573 |
| K11927 | 0.95 | 0.46 | 0.037595 | 0.095093 |
| K10540 | -0.81 | 0.39 | 0.037627 | 0.095113 |
| K07788 | -0.59 | 0.28 | 0.037676 | 0.095118 |
| K20483 | 0.50 | 0.24 | 0.03767 | 0.095118 |
| K03741 | 0.78 | 0.37 | 0.03782 | 0.095423 |
| K06333 | 0.71 | 0.34 | 0.037869 | 0.095487 |
| K02398 | -0.65 | 0.31 | 0.037952 | 0.095635 |
| K21556 | 0.70 | 0.33 | 0.03798 | 0.095647 |
| K04769 | 0.53 | 0.25 | 0.03809 | 0.095866 |
| K03281 | 0.88 | 0.42 | 0.038127 | 0.095899 |
| K00348 | 0.98 | 0.47 | 0.03825 | 0.096145 |
| K19119 | 0.65 | 0.31 | 0.038273 | 0.096145 |
| K01462 | 0.22 | 0.11 | 0.038344 | 0.096264 |
| K01626 | 0.36 | 0.17 | 0.038395 | 0.096305 |
| K10117 | 0.46 | 0.22 | 0.038408 | 0.096305 |
| K15396 | -0.70 | 0.34 | 0.038448 | 0.096345 |
| K19162 | -0.53 | 0.26 | 0.038499 | 0.096415 |
| K09013 | 0.32 | 0.16 | 0.038739 | 0.096956 |
| K12373 | 0.88 | 0.42 | 0.038918 | 0.097342 |
| K03425 | -0.81 | 0.39 | 0.039009 | 0.097389 |
| K03437 | 0.43 | 0.21 | 0.03898 | 0.097389 |
| K07447 | 0.23 | 0.11 | 0.038998 | 0.097389 |
| K00383 | -0.51 | 0.25 | 0.039153 | 0.09763 |
| K11473 | -0.53 | 0.25 | 0.039132 | 0.09763 |
| K09862 | -0.75 | 0.36 | 0.039239 | 0.097784 |
| K04761 | -0.67 | 0.33 | 0.039319 | 0.097791 |
| K07273 | -0.55 | 0.27 | 0.039326 | 0.097791 |
| K07490 | -0.70 | 0.34 | 0.039339 | 0.097791 |
| K11065 | -0.85 | 0.41 | 0.039335 | 0.097791 |
| K00351 | 0.93 | 0.45 | 0.039609 | 0.098271 |
| K03737 | 0.59 | 0.28 | 0.039588 | 0.098271 |
| K05709 | -0.52 | 0.25 | 0.039619 | 0.098271 |
| K07393 | -0.68 | 0.33 | 0.039629 | 0.098271 |
| K15922 | 0.96 | 0.47 | 0.0398 | 0.098634 |
| K10013 | -0.72 | 0.35 | 0.039855 | 0.09871 |
| K01470 | 0.98 | 0.48 | 0.040058 | 0.099152 |
| K17236 | -0.79 | 0.38 | 0.040305 | 0.099702 |
| K01095 | -0.79 | 0.39 | 0.040394 | 0.099863 |
| K06861 | -0.64 | 0.31 | 0.040488 | 0.100033 |
| K22432 | -0.50 | 0.24 | 0.040573 | 0.100183 |
| K09698 | -0.56 | 0.27 | 0.040708 | 0.100454 |
| K06408 | 0.70 | 0.34 | 0.040747 | 0.100489 |
| K19355 | 0.74 | 0.36 | 0.040805 | 0.100571 |
| K02858 | -0.70 | 0.34 | 0.04083 | 0.100572 |
| K07035 | 0.57 | 0.28 | 0.040873 | 0.100617 |
| K05816 | -0.67 | 0.33 | 0.040992 | 0.100843 |
| K11685 | -0.38 | 0.18 | 0.041015 | 0.100843 |
| K01169 | -0.60 | 0.29 | 0.04115 | 0.101017 |
| K01698 | 0.53 | 0.26 | 0.041132 | 0.101017 |
| K08997 | -0.70 | 0.34 | 0.041161 | 0.101017 |
| K01858 | 0.86 | 0.42 | 0.041194 | 0.101039 |
| K03342 | -0.52 | 0.25 | 0.041221 | 0.101042 |
| K00239 | 0.85 | 0.41 | 0.041311 | 0.101104 |
| K02493 | -0.27 | 0.13 | 0.041321 | 0.101104 |
| K03533 | -0.62 | 0.30 | 0.041321 | 0.101104 |
| K00847 | 0.38 | 0.19 | 0.041355 | 0.101124 |
| K01525 | -0.69 | 0.34 | 0.041379 | 0.101124 |
| K02168 | -0.69 | 0.34 | 0.041565 | 0.101516 |
| K02803 | 0.89 | 0.44 | 0.041776 | 0.101971 |
| K04518 | 0.46 | 0.23 | 0.041866 | 0.102067 |
| K07684 | -0.63 | 0.31 | 0.04185 | 0.102067 |
| K03185 | -0.55 | 0.27 | 0.041985 | 0.102235 |
| K21430 | -0.48 | 0.24 | 0.041977 | 0.102235 |
| K07979 | 0.40 | 0.19 | 0.042044 | 0.102315 |
| K07576 | 0.70 | 0.34 | 0.042206 | 0.102598 |
| K10041 | -0.54 | 0.26 | 0.042211 | 0.102598 |
| K19226 | -0.68 | 0.33 | 0.042291 | 0.102733 |
| K03919 | -0.66 | 0.32 | 0.042363 | 0.102845 |
| K03980 | -0.65 | 0.32 | 0.042525 | 0.10317 |
| K15599 | 0.58 | 0.29 | 0.042548 | 0.10317 |
| K15551 | -0.72 | 0.35 | 0.042628 | 0.103304 |
| K03580 | -0.42 | 0.21 | 0.042883 | 0.103796 |
| K07663 | -0.57 | 0.28 | 0.042908 | 0.103796 |
| K11535 | -0.65 | 0.32 | 0.042902 | 0.103796 |
| K14682 | -0.49 | 0.24 | 0.043019 | 0.104002 |
| K15724 | -0.71 | 0.35 | 0.043076 | 0.104078 |
| K01666 | -0.78 | 0.38 | 0.043134 | 0.104156 |
| K19271 | 0.89 | 0.44 | 0.043258 | 0.104391 |
| K15770 | -0.68 | 0.34 | 0.043329 | 0.104502 |
| K00337 | 0.84 | 0.41 | 0.043425 | 0.104671 |
| K00344 | -0.74 | 0.36 | 0.043544 | 0.104895 |
| K12973 | -0.72 | 0.35 | 0.043706 | 0.105211 |
| K21471 | 0.50 | 0.25 | 0.043727 | 0.105211 |
| K05809 | -0.33 | 0.16 | 0.043759 | 0.105225 |
| K11392 | -0.73 | 0.36 | 0.043888 | 0.105471 |
| K11752 | 0.55 | 0.27 | 0.043997 | 0.105671 |
| K02464 | -0.70 | 0.35 | 0.044231 | 0.106165 |
| K06295 | 0.70 | 0.35 | 0.044255 | 0.106165 |
| K01704 | 0.26 | 0.13 | 0.044342 | 0.106311 |
| K07803 | -0.70 | 0.34 | 0.044486 | 0.106592 |
| K02065 | 0.76 | 0.38 | 0.04498 | 0.107714 |
| K06298 | 0.70 | 0.35 | 0.045009 | 0.107718 |
| K12143 | -0.45 | 0.22 | 0.045124 | 0.10793 |
| K01808 | -0.41 | 0.20 | 0.045225 | 0.108108 |
| K13059 | 0.64 | 0.32 | 0.045363 | 0.108373 |
| K01908 | -0.70 | 0.35 | 0.045539 | 0.108647 |
| K16509 | -0.63 | 0.31 | 0.045558 | 0.108647 |
| K16692 | -0.58 | 0.29 | 0.045523 | 0.108647 |
| K03770 | 0.86 | 0.43 | 0.045606 | 0.108697 |
| K01624 | 0.30 | 0.15 | 0.045896 | 0.109325 |
| K21907 | -0.57 | 0.28 | 0.045947 | 0.109382 |
| K21990 | -0.52 | 0.26 | 0.045991 | 0.109422 |
| K18838 | -0.59 | 0.29 | 0.046037 | 0.109467 |
| K07334 | -0.60 | 0.30 | 0.046071 | 0.109483 |
| K07250 | -0.83 | 0.41 | 0.046109 | 0.10951 |
| K05818 | -0.43 | 0.22 | 0.046204 | 0.109669 |
| K11249 | -0.59 | 0.29 | 0.046273 | 0.109705 |
| K16301 | -0.50 | 0.25 | 0.046265 | 0.109705 |
| K03632 | -0.54 | 0.27 | 0.046485 | 0.110144 |
| K07806 | -0.50 | 0.25 | 0.046656 | 0.110484 |
| K03491 | 0.54 | 0.27 | 0.046831 | 0.110833 |
| K03815 | -0.50 | 0.25 | 0.046917 | 0.110971 |
| K05541 | -0.58 | 0.29 | 0.047045 | 0.111145 |
| K06176 | -0.73 | 0.36 | 0.047022 | 0.111145 |
| K09861 | -0.31 | 0.16 | 0.047255 | 0.111538 |
| K21030 | 0.68 | 0.34 | 0.047266 | 0.111538 |
| K03805 | -0.72 | 0.36 | 0.047324 | 0.111608 |
| K07120 | -0.62 | 0.31 | 0.047496 | 0.11195 |
| K19701 | 0.42 | 0.21 | 0.047662 | 0.112274 |
| K02836 | 0.24 | 0.12 | 0.047712 | 0.112328 |
| K09015 | 0.78 | 0.39 | 0.047744 | 0.112337 |
| K07717 | 0.41 | 0.21 | 0.047875 | 0.112579 |
| K01174 | -0.20 | 0.10 | 0.047983 | 0.112769 |
| K08168 | 0.79 | 0.40 | 0.048023 | 0.112796 |
| K01684 | -0.83 | 0.42 | 0.048089 | 0.112886 |
| K08306 | -0.59 | 0.30 | 0.048315 | 0.113351 |
| K07104 | -0.39 | 0.20 | 0.048465 | 0.11357 |
| K07586 | -0.66 | 0.33 | 0.04844 | 0.11357 |
| K03809 | -0.61 | 0.31 | 0.048593 | 0.113805 |
| K07566 | -0.32 | 0.16 | 0.048783 | 0.114184 |
| K02804 | 0.87 | 0.44 | 0.04894 | 0.114486 |
| K00697 | -0.53 | 0.27 | 0.049014 | 0.114593 |
| K02769 | 0.50 | 0.25 | 0.049132 | 0.114605 |
| K07407 | -0.45 | 0.23 | 0.049129 | 0.114605 |
| K12308 | 0.46 | 0.23 | 0.049103 | 0.114605 |
| K20626 | 0.28 | 0.14 | 0.049133 | 0.114605 |
| K18841 | -0.36 | 0.18 | 0.049223 | 0.114749 |
| K12555 | -0.59 | 0.30 | 0.049274 | 0.114802 |
| K11530 | -0.45 | 0.23 | 0.049397 | 0.115022 |
| K02825 | -0.53 | 0.27 | 0.049597 | 0.115421 |
| K13650 | -0.56 | 0.28 | 0.049635 | 0.115445 |
| K02015 | -0.30 | 0.15 | 0.049686 | 0.115495 |
| K11250 | -0.66 | 0.33 | 0.049742 | 0.11556 |
| K10986 | -0.49 | 0.25 | 0.049951 | 0.115979 |
| K22104 | -0.46 | 0.23 | 0.050004 | 0.116035 |
| K00645 | -0.46 | 0.23 | 0.050057 | 0.116063 |
| K08354 | -0.44 | 0.23 | 0.050073 | 0.116063 |
| K01846 | -0.56 | 0.28 | 0.05026 | 0.11643 |
| K01845 | 0.51 | 0.26 | 0.050593 | 0.117134 |

Supplementary Table S5. GBM modules at 6 months.

| **Module** | **Enzyme** | **Coefficent** | **SD** | **P raw** | **P FDR adjusted** |
| --- | --- | --- | --- | --- | --- |
| MGB009_Histamine synthesis_K01590 | Histidine decarboxylase | 0.53 | 0.17 | 0.003 | 0.189 |
| MGB010_Histamine degradation_K00276 | Amine oxidase | 0.31 | 0.13 | 0.019 | 0.211 |
| MGB005_Tryptophan synthesis_K01696 | Tryptophan synthase beta chain | 0.14 | 0.05 | 0.011 | 0.211 |
| MGB043_Acetate synthesisI_K00656 | Formate C-acetyltransferase | 0.11 | 0.05 | 0.022 | 0.211 |
| MGB045_Acetate synthesisIII_K00656 | Formate C-acetyltransferase | 0.11 | 0.05 | 0.022 | 0.211 |
| MGB044_Acetate synthesisII_K00656 | Formate C-acetyltransferase | 0.11 | 0.05 | 0.022 | 0.211 |

|  |  |  |  |  |
| --- | --- | --- | --- | --- |
|  |  |  |  |  |

Supplementary Table S6. GBM modules at 12 months.

| **Module** | **Enzyme** | **Coefficent** | **SD** | **P raw** | **P FDR adjusted** |
| --- | --- | --- | --- | --- | --- |
| MGB005_Tryptophan synthesis_K01817 | Phosphoribosylanthranilate isomerase | 0.37 | 0.13 | 0.003 | 0.011 |
| MGB005_Tryptophan synthesis_K13498 | Indole-3-glycerol phosphate synthase | -0.31 | 0.14 | 0.027 | 0.062 |
| MGB049_Tryptophan degradation_K01667 | Tryptophanase | 0.52 | 0.17 | 0.002 | 0.010 |
| MGB043_Acetate synthesisI_K00171 | Pyruvate ferredoxin oxidoreductase | 0.54 | 0.15 | 0.0004 | 0.002 |
| MGB043_Acetate synthesisI_K00656 | Formate C-acetyltransferase | 0.12 | 0.06 | 0.028 | 0.062 |
| MGB043_Acetate synthesisI_K03737 | Ferredoxin/flavodoxin oxidoreductase | 0.28 | 0.12 | 0.024 | 0.062 |
| MGB044_Acetate synthesisII_K00171 | Pyruvate ferredoxin oxidoreductase | 0.54 | 0.15 | 0.0004 | 0.002 |
| MGB044_Acetate synthesisII_K00656 | Formate C-acetyltransferase | 0.12 | 0.06 | 0.028 | 0.062 |
| MGB044_Acetate synthesisII_K01905 | CoA ligase | 0.31 | 0.15 | 0.043 | 0.086 |
| MGB045_Acetate synthesisIII_K00171 | Pyruvate ferredoxin oxidoreductase | 0.54 | 0.15 | 0.0004 | 0.002 |
| MGB045_Acetate synthesisIII_K00656 | Formate C-acetyltransferase | 0.12 | 0.06 | 0.028 | 0.062 |
| MGB045_Acetate synthesisIII_K03737 | Ferredoxin/flavodoxin oxidoreductase | 0.28 | 0.12 | 0.024 | 0.062 |
| MGB045_Acetate synthesisIII_K18118 | Succinyl-CoA:acetate CoA-transferase | -0.68 | 0.14 | 1.59E-06 | 3.03E-05 |
| MGB052_Butyrate synthesisI_K00248 | Butyryl-CoA dehydrogenase | 0.28 | 0.14 | 0.043 | 0.086 |
| MGB052_Butyrate synthesisI_K00929 | Butyrate kinase | 0.48 | 0.21 | 0.020 | 0.057 |
| MGB053_Butyrate synthesisII_K00248 | Butyryl-CoA dehydrogenase | 0.28 | 0.14 | 0.043 | 0.086 |
| MGB053_Butyrate synthesisII_K01034 | Acetate CoA/acetoacetate CoA-transferase alpha subunit | -0.43 | 0.12 | 0.0006 | 0.002 |
| MGB053_Butyrate synthesisII_K01035 | Acetate CoA/acetoacetate CoA-transferase beta subunit | -0.36 | 0.09 | 0.0001 | 0.0007 |
| MGB053_Butyrate synthesisII_K19709 | Acetate CoA-transferase | -0.69 | 0.14 | 8.3E-07 | 3.03E-05 |
| MGB048_Propionate synthesisI_K00932 | Propionate kinase | -0.69 | 0.14 | 1.29E-06 | 3.03E-05 |
| MGB054_Propionate synthesisII_K01026 | Propionate CoA-transferase | 0.79 | 0.17 | 3.14E-06 | 4.48E-05 |
| MGB055_Propionate synthesisII_K01848 | Methylmalonyl-CoA mutase, N-terminal domain | -0.44 | 0.11 | 6.68E-05 | 0.0004 |
| MGB055_Propionate synthesisII_K01849 | Methylmalonyl-CoA mutase, C-terminal domain | -0.57 | 0.13 | 1.13E-05 | 0.0001 |
| MGB055_Propionate synthesisII_K05606 | Methylmalonyl-CoA/ethylmalonyl-CoA epimerase | -0.27 | 0.12 | 0.019 | 0.057 |
| MGB056_Propionate degradationI_K01659 | 2-methylcitrate synthase | -0.40 | 0.14 | 0.003 | 0.010 |
| MGB056_Propionate degradationI_K01682 | Aconitate hydratase 2 / 2-methylisocitrate dehydratase | -0.66 | 0.14 | 4.73E-06 | 5.39E-05 |
| MGB056_Propionate degradationI_K01720 | 2-methylcitrate dehydratase | -0.65 | 0.15 | 1.43E-05 | 0.0001 |
| MGB056_Propionate degradationI_K01908 | Propionyl-CoA synthetase | -0.45 | 0.15 | 0.002 | 0.010 |
| MGB056_Propionate degradationI_K03417 | Methylisocitrate lyase | -0.45 | 0.13 | 0.0006 | 0.0028 |

|  |  |  |  |  |
| --- | --- | --- | --- | --- |

Supplementary Table S7. Machine learning model metrics for prediction of food sensitisation by 12 months of age.

|  | L1 Lasso Logistic Regression | L2 Ridge Logistic Regression | Elastic Net | XGBOOST | Random Forest | SVM | Light Gradient Boosting Machines |
| --- | --- | --- | --- | --- | --- | --- | --- |
| Accuracy | 0.6 | 0.7 | 0.7 | 0.6 | 0.8 | 0.7 | 0.8 |
| ROC | 0.76 | 0.76 | 0.84 | 0.68 | 0.84 | 0.12 | 0.96 |
| Log Loss | 0.69 | 0.75 | 0.59 | 0.79 | 0.51 | 0.85 | 0.38 |
| Confusion Matrix | (3,2),(2,3) | (4,1),(2,3) | (3,2),(1,4) | (4,1),(3,2) | (4,1),(1,4) | (4,1),(2,3) | (5,0),(2,3) |
| Precision class 0 | 0.6 | 0.67 | 0.75 | 0.57 | 0.8 | 0.67 | 0.71 |
| Precision class 1 | 0.6 | 0.75 | 0.67 | 0.67 | 0.8 | 0.75 | 1 |
| Recall for class 0 | 0.6 | 0.8 | 0.6 | 0.8 | 0.8 | 0.8 | 1 |
| Recall for class 1 | 0.6 | 0.6 | 0.8 | 0.4 | 0.8 | 0.6 | 0.6 |
| F1 score - class 0 | 0.6 | 0.73 | 0.67 | 0.67 | 0.8 | 0.73 | 0.83 |
| F1 score - class 1 | 0.6 | 0.67 | 0.73 | 0.5 | 0.8 | 0.67 | 0.75 |
